# Near-Infrared Afterglow ONOO^–^-Triggered Nanoparticles for Real-Time Monitoring and Treatment of Early Ischemic Stroke

**DOI:** 10.1021/acsami.3c08033

**Published:** 2023-09-20

**Authors:** Liping Zhang, Ya-chao Wang, Yuqi Liao, Qian Zhang, Xia Liu, Dongxia Zhu, Haixing Feng, Martin R. Bryce, Lijie Ren

**Affiliations:** † Department of Neurology, Inst Translat Med, The First Affiliated Hospital of Shenzhen University, Shenzhen Second People’s Hospital, Shenzhen 518035, P. R. China; ‡ Key Laboratory of Nanobiosensing and Nanobioanalysis at Universities of Jilin Province, Department of Chemistry, Northeast Normal University, 5268 Renmin Street, Changchun, Jilin 130024, P. R. China; § Department of Chemistry Durham, University Durham, Durham DH1 3LE, U.K.

**Keywords:** ischemic stroke, peroxynitrite, near-infrared afterglow, aggregation-induced emission, real-time monitoring, theranostic, BODIPY

## Abstract

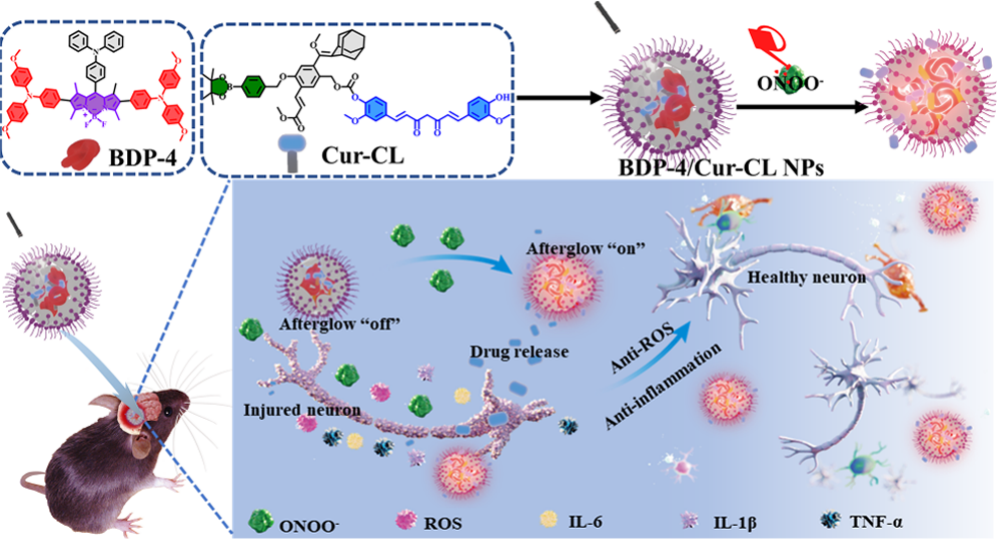

Early detection and drug intervention with the appropriate timing and dosage are the main clinical challenges for ischemic stroke (IS) treatment. The conventional therapeutic agents relay fluorescent signals, which require real-time external light excitation, thereby leading to inevitable autofluorescence and poor tissue penetration. Herein, we report endogenous peroxynitrite (ONOO^–^)-activated **BDP-4/Cur-CL** NPs that release NIR afterglow signals (λ_max_ 697 nm) for real-time monitoring of the progression of ischemia reperfusion (I/R) brain injury while releasing curcumin for the safe treatment of IS. The **BDP-4/Cur-CL** NPs exhibited bright NIR afterglow luminescence (maximum 732-fold increase), superb sensitivity (LOD = 82.67 nM), high energy-transfer efficiency (94.6%), deep tissue penetration (20 mm), outstanding antiapoptosis, and anti-inflammatory effects. The activated NIR afterglow signal obtained in mice with middle cerebral artery occlusion (MCAO) showed three functions: (i) the **BDP-4/Cur-CL** NPs are rapidly activated by endogenous ONOO^–^, instantly illuminating the lesion area, distinguishing I/R damage from normal areas, which can be successfully used for endogenous ONOO^–^ detection in the early stage of IS; (ii) real-time reporting of *in situ* generation and dynamic fluctuations of endogenous ONOO^–^ levels in the lesion area, which is of great value in monitoring the evolutionary mechanisms of IS; and (iii) dynamic monitoring of the release of curcumin drug for safe treatment. Indeed, the released curcumin effectively decreased apoptosis, enhanced survival, alleviated neuroinflammation, reduced brain tissue loss, and improved the cognition of MCAO stroke mice. This work is the first example of afterglow luminescence for early diagnosis, real-time reporting, drug tracing, and treatment for IS.

## Introduction

Ischemic stroke (IS), caused by inadequate oxygen and blood supply to the brain, is a leading cause of death and severe long-term disability worldwide.^1−3^ Currently, thrombolysis within the effective time window has been considered as the gold standard for clinical IS treatment.^4,5^ Unfortunately, oxidative stress will occur suddenly after thrombolysis, leading to the release of toxic reactive oxygen and nitrogen species (RONS), thereby worsening secondary ischemia reperfusion (I/R) brain injury, leaving nearly 40% of survivors disabled.^6−8^ Notably, as a distinct component of oxidative stress, peroxynitrite (ONOO^–^) shows ultrahigh toxicity compared to other RONS due to its destructive nitrification damage to lipid, mitochondria, and DNA.^9,10^ Also, the microenvironmental concentration of ONOO^–^ in the lesion area correlates positively with neuroinflammation and neurotoxicity after the IS.^11,12^ It follows that the byproducts (notably ONOO^–^) have a negative impact on the treatment of IS. Hence, how to fully utilize and consume ONOO^–^ are crucial clinical factors.

Optical imaging, with high sensitivity and excellent spatiotemporal resolution to reveal pathological processes at the molecular and microscopic levels, has become an irreplaceable technique in diagnostic biology.^13−16^ For example, aminophenol fluorescein (APF) and hydroxyphenyl fluorescein (HPF) are widely employed for the detection of ONOO^–^.^11^ A series of ONOO^–^-triggered NIR fluorescence (FL) probes constructed by Feng and co-workers have been exploited in biological applications.^17,18^ However, these reagents are far from ideal, and they possess significant drawbacks: (i) the required real-time light excitation results in inevitable autofluorescence and poor tissue penetration;^16,19−24^ (ii) poor selectivity reduces the specific detection of ONOO^–^.^25^ In contrast, afterglow luminescent materials generally exhibit deeper tissue penetration and higher selectivity compared with traditional fluorescent reagents due to the luminescent signals being acquired without external light excitation.^13,26−28^ Notably, a series of ONOO^–^-triggered probes based on Schaap’s adamantylidene-1,2-dioxetane show great potential for diagnosis because their afterglow signal can be “turned on” only in the presence of disease biomarkers/microenvironments.^14,27,29,30^ Despite these exciting pioneer studies, near-infrared (NIR) afterglow luminescent (≥650 nm) materials are still scarce and are desirable for brain imaging with increased tissue penetration depth. Studies on the real-time visualization of ONOO^–^-targeted activatable NIR afterglow for I/R brain damage in IS are rare.

The appropriate timing and dosage of drugs, accompanied by early detection, are the main challenges in effective clinical treatment for IS.^31−34^ Curcumin (Cur) is a naturally occurring substance that exhibits positive neuroprotective effects for IS because of its antioxidation, antiapoptosis, and anti-inflammatory properties.^35,36^ However, curcumin possesses poor water solubility, non-negligible cellular toxicity, and fast metabolism, causing suboptimal treatment efficacy for IS.^37^ Importantly, the release of conventional potential therapeutic agents cannot be controlled according to the degree of disease progression, resulting in inaccurate dosage of drugs and unsafe stroke treatment.^38−40^ Therefore, exploring endogenous ONOO^–^ as a trigger to obtain an NIR afterglow image-guided system that integrates real-time evaluation of ONOO^–^ levels in the I/R brain injury area with dynamic monitoring of the release status of curcumin is of great significance for safe stroke treatment.

**Figure 1:**
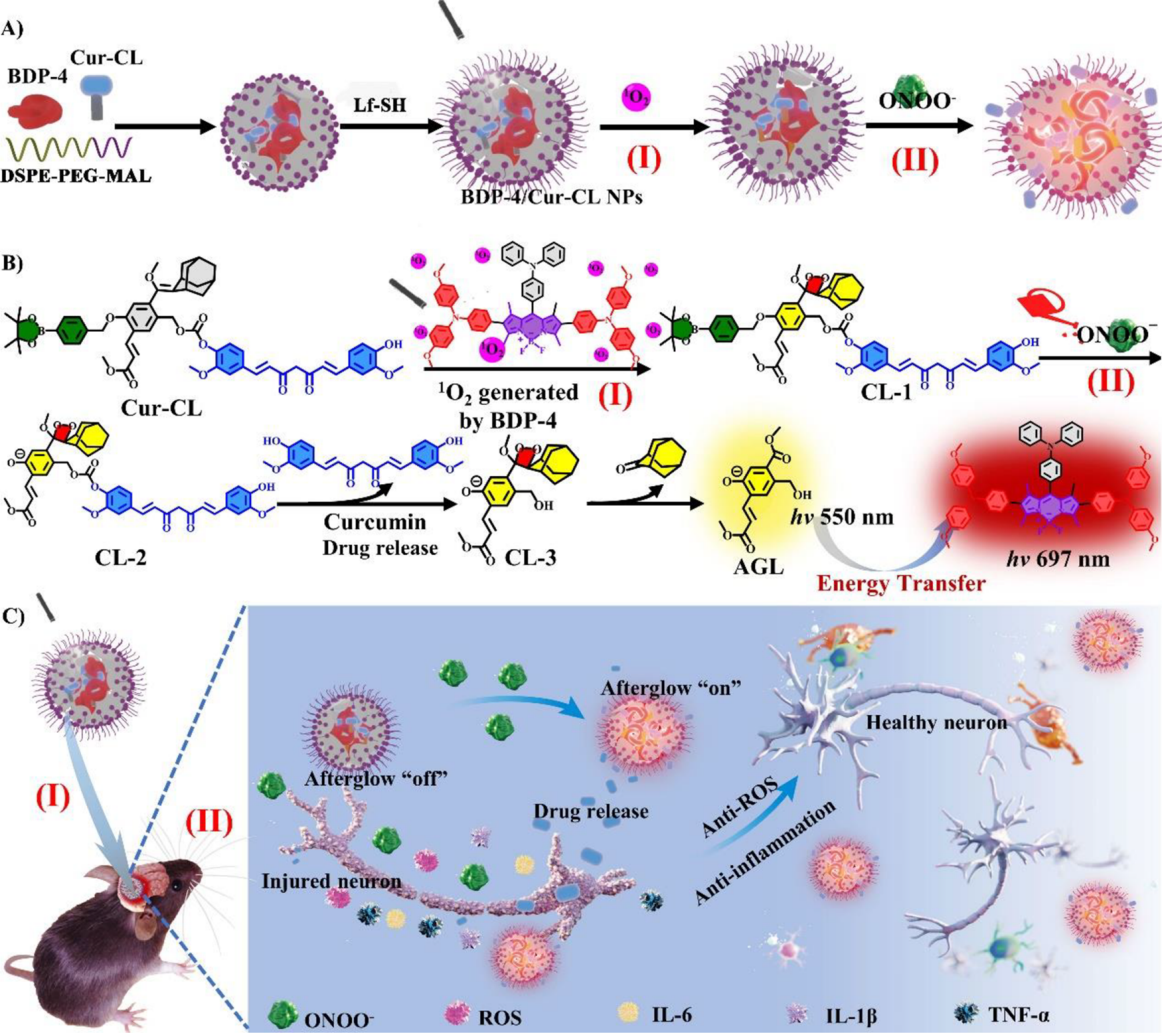
(A) Schematic showing the **BDP-4/Cur-CL** NP fabrication, drug release, and NIR afterglow luminescence. (B) The molecular structure conversion protocols. (C) Schematic illustration of **BDP-4/Cur-CL** NPs for IS.

In this work, we turn an enemy into a friend and design ONOO^–^-activated NIR afterglow image-guided nanoparticles (NPs) (named **BDP-4/Cur-CL** NPs) consisting of **BDP-4** and **Cur-CL** units (Figure 1A). The system can specifically and sensitively evaluate endogenous ONOO^–^ levels to reveal the progression of I/R brain injury as well as dynamically monitor curcumin release for safe IS treatment. Upon the irradiation of **BDP-4/Cur-CL** NPs, **Cur-CL** was converted to **CL-1** by generating ^1^O_2_ from **BDP-4** (Figure 1B(I)). Subsequently, preirradiated **BDP-4/Cur-CL** NPs were cleaved by the endogenous ONOO^–^ (at I/R brain injury areas) accompanying the simultaneous in situ-activated NIR afterglow luminescence (λ_max_ 697 nm) and curcumin drug release (Figure 1B(II),C). The **BDP-4/Cur-CL** NPs exhibited bright NIR afterglow luminescence (maximum 732-fold increase), high sensitivity (limit of detection, LOD = 82.67 nM), high energy-transfer efficiency (94.6%), deep tissue penetration (20 mm), and outstanding antiapoptosis and anti-inflammatory effects. By using this intricate design, **BDP-4/Cur-CL** NPs activate NIR afterglow signals and offer triple functions during I/R brain injury onset and progression *in vivo* in mice. These functions are (i) accurately distinguishing between I/R injury and normal areas; (ii) real-time reporting of endogenous ONOO^–^ levels in the lesion area; and (iii) dynamic monitoring of the release status of curcumin. In addition, the **BDP-4/Cur-CL** NPs effectively decreased apoptosis, enhanced survival rates, alleviated neuroinflammation, reduced infarct volume, and enhanced stroke outcomes. To the best of our knowledge, this is the first report of a theranostic prodrug with NIR afterglow turn-on and activation for synergistic IS therapy.

## Results and Discussion

### Synthesis and Theoretical Calculations

In recent years, BODIPY-based theranostics have achieved significant success because their optical properties can be systematically tuned by chemical modifications.^41^ The introduction of strong electron donors (D) into the methylated BODIPY acceptor (A) core can significantly improve the photophysical properties and the ^1^O_2_ generation capacity of the compound, thereby optimizing its afterglow luminescence performance. The synthetic routes for **Cur-CL** and AIEgens (named **BDP-1**, **BDP-3**, and **BDP-4**) are shown in the Supporting Information (Schemes S1 and S2), and their chemical structures were validated by ^1^H and ^13^C NMR spectroscopy and mass spectrometry (Figures S2–S24). **BDP-2** was synthesized based on the previously reported literature.^42^ The NPs were obtained by amphiphilic polymer (DSPE-PEG-MAL) encapsulation.^16^ Sulfhydrylated Lactoferrin (Lf-SH) is an iron-binding glycoprotein, which can effectively transport NPs into brain cells.^43^ Time-dependent density functional theory (TD-DFT) calculations gave insight into the electronic properties of **BDP-1**, **BDP-2**, **BDP-3**, and **BDP-4** (Figure S25). The highest occupied molecular orbitals (HOMOs) are mainly localized on the triphenylamine (TPA) units, whereas the lowest unoccupied molecular orbitals (LUMOs) are located on the methylated BODIPY core, with significant HOMO–LUMO separation. The energy gap between the HOMO and the LUMO of **BDP-1**, **BDP-2**, **BDP-3**, and **BDP-4** is 2.603, 2.416, 2.325, and 2.202 eV, respectively. The Δ*E*_ST_ (the energy gap between the S1 and the T1 states) of **BDP-1**, **BDP-2**, **BDP-3**, and **BDP-4** is 1.46, 1.37, 1.35, and 1.33 eV, respectively (Figure S25). The smaller Δ*E*_ST_ value in **BDP-4**, compared with that in **BDP-1**, **BDP-2**, and **BDP-3**, promotes the generation of the triplet photoexcited state through intersystem crossing (ISC) and further enhances the generation of ^1^O_2_.^15,44^

### Optical Properties of AIEgens

**Figure 2:**
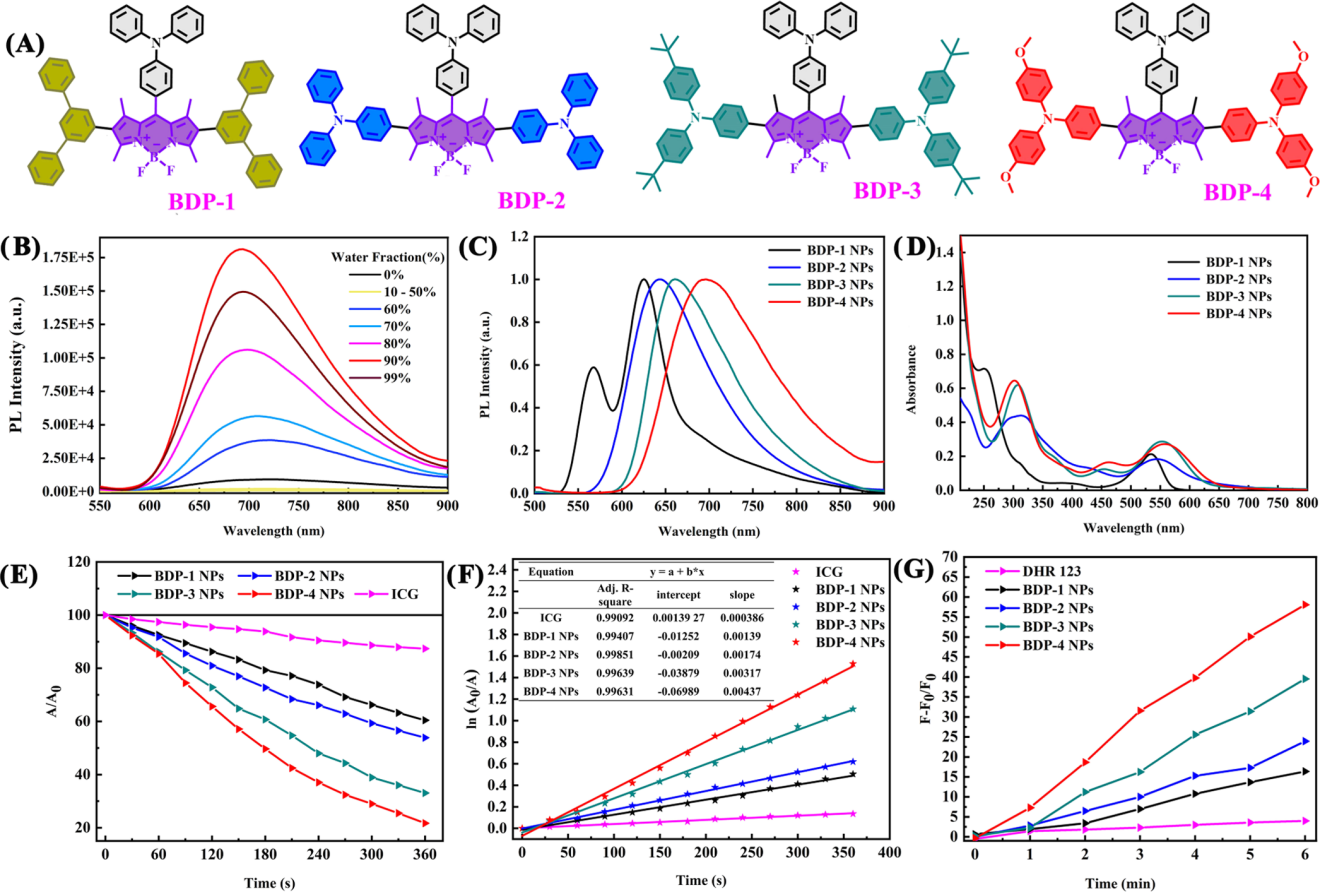
(A) Structural formulas of **BDP-1**, **BDP-2**, **BDP-3**, and **BDP-4**. (B) Emission spectra of **BDP-4** (10^–5^ M) in THF–water mixtures with different water fractions (0–99% v/v) at room temperature. (C) Photoluminescence (PL) and (D) UV–vis absorption spectra of the NPs. (E) Comparison of the decomposition rates of the NPs under white-light irradiation (20 mW cm^–2^). (F) Time-dependent kinetics of ^1^O_2_ generation. *A*_0_ is the absorption of ICG without irradiation. *A* = the real-time absorption of ICG with different irradiation times. (G) Plot of the relative emission intensity (*F* – *F*_0_/*F*_0_) of a DHR123 (10^–5^ M) solution containing different NPs (10^–5^ M) under white-light irradiation (20 mW cm^–2^). *F*_0_ is the emission of DHR123 (526 nm) without irradiation. *F* = real-time emission of DHR123 (526 nm) with different irradiation times.

To investigate the aggregation-induced emission (AIE) features of **BDP-1**, **BDP-2**, **BDP-3**, and **BDP-4**, the emission spectra were recorded in water–tetrahydrofuran (THF) mixtures with different water fractions (Figures 2B and S26). Upon gradual addition of water into THF (water fraction: fw ≤ 30%), all of the compounds show weakened fluorescence. However, photoluminescence (PL) intensities of **BDP-1**, **BDP-2**, **BDP-3**, and **BDP-4** were then significantly enhanced (fw ≥ 30–90%), indicating a typical AIE effect. However, the fluorescence intensity decreased when the water fraction was higher than 90%, which can be interpreted as the formation of amorphous particles.^45^ The dual emission peak of **BDP-1** (Figure S26A) can be attributed to the combined effect of the locally excited (LE) state (569 nm) and twisted intramolecular charge-transfer (TICT) state (625 nm).^46,47^ Compared with the BODIPY complexes, the corresponding NPs exhibit similar emission peaks but with much brighter emission in water (Figures 2C and S27). The PL maxima of **BDP-1** NPs, **BDP-2** NPs, **BDP-3** NPs, and **BDP-4** NPs are 625, 645, 662, and 697 nm, respectively, demonstrating a beneficial red shift with the increasing electron-donating ability of the BODIPY core. The fluorescence quantum yields (QYs) of **BDP-1** NPs (625 nm), **BDP-2** NPs (645 nm), **BDP-3** NPs (662 nm), and **BDP-4** NPs (697 nm) in water are 39, 27, 23, and 22%, respectively. In Figure 2D, the shorter wavelength absorption bands at around 200–400 nm are attributed to the π–π* and n–π* transitions of the donors in the conjugated backbone, whereas the longer absorption bands at around 450–650 nm are ascribed to the π–π* transition of the BODIPY unit, which is beneficial for matching the chemiluminescence (CL) spectra of **Cur-CL** to facilitate efficient energy transfer within NPs. The UV–vis absorption and emission spectra of **Cur-CL** NPs were obtained. In Figure S28, the strong absorption of **Cur-CL** NPs in the 400–500 nm range is ascribed to the n–π* transition in the conjugated backbone. The **Cur-CL** NPs exhibited fluorescence around 510 nm, which is significantly different from their afterglow emission (550 nm). Furthermore, the **BDP-1** NPs, **BDP-2** NPs, **BDP-3** NPs, **BDP-4** NPs, and **Cur-CL** NPs exhibited emission and absorption spectra in PBS at different pH values (6.0–8.8) similar to those in water (Figures S28–S32).

Furthermore, the ^1^O_2_ generation ability of the AIEgens NPs was monitored by using the standard dye indocyanine green (ICG) as an indicator.^48^ Upon irradiation of ICG solutions (5 μg mL^–1^) in the presence of **BDP-1** NPs, **BDP-2** NPs, **BDP-3** NPs, and **BDP-4** NPs (30 μg mL^–1^), the absorption peak of ICG at 790 nm gradually decreased in intensity (Figures 2E and S35), confirming the efficient ^1^O_2_ generation by these NPs. As shown in Figure 2F, ^1^O_2_ generation follows first-order kinetics, with the slope in the order: **BDP-1** NPs (1.39 × 10^–3^) < **BDP-2** NPs (1.74 × 10^–3^) < **BDP-3** NPs (3.17× 10^–3^) < **BDP-4** NPs (4.37 × 10^–3^). A steeper slope represents a quicker decay rate of ICG and higher ability to generate ^1^O_2_.^49^ To evaluate the type I ROS generation ability of the NPs, dihydrorhodamine 123 (DHR123) and HPF were used as an indicator for O_2_^•–^ and ^•^OH.^50^ As expected, upon irradiation of DHR123 (10^–5^ M) solutions in the presence of **BDP-1** NPs, **BDP-2** NPs, **BDP-3** NPs, and **BDP-4** NPs (10^–5^ M), the fluorescence intensity of DHR123 at 526 nm gradually increased (Figures 2G and S37), indicating that the NPs possess an O_2_^•–^ generation ability. Upon irradiation of HPF (10^–5^ M) solutions in the presence of **BDP-1** NPs, **BDP-2** NPs, **BDP-3** NPs, and **BDP-4** NPs (10^–5^ M), the fluorescence intensity of HPF at 516 nm was almost unchanged (Figure S38), indicating that these NPs do not possess ^•^OH generation ability. Overall, the **BDP-4** NPs possess the most favorable photophysical properties of the series.

### Characterization of ONOO^–^-Activatable NIR Afterglow Luminescent Nanoprobes

**Figure 3:**
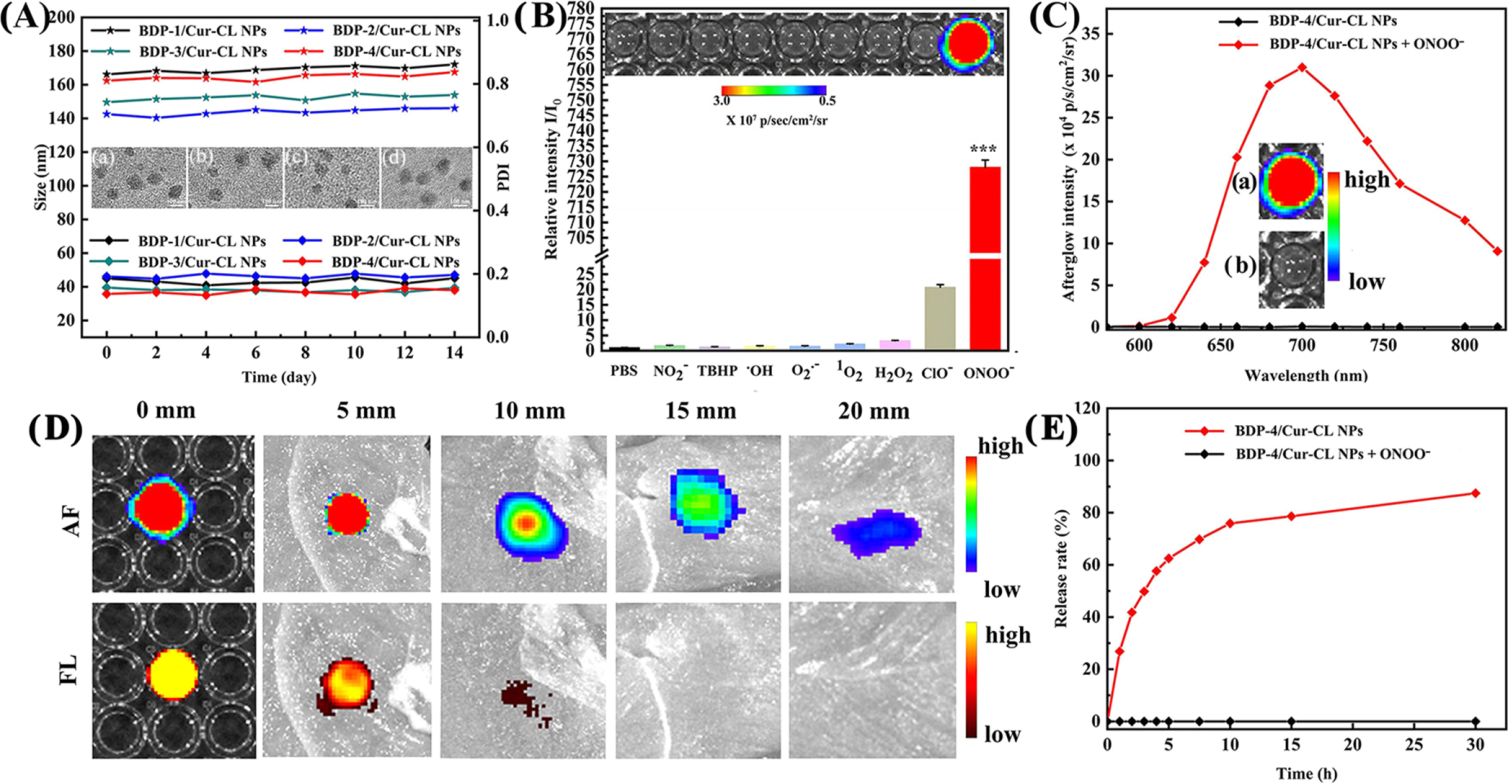
(A) Size changes of different NPs during 14 days: the inset is the TEM images of (a) **BDP-1/Cur-CL** NPs, (b) **BDP-2/Cur-CL** NPs, (c) **BDP-3/Cur-CL** NPs, and (d) **BDP-4/Cur-CL** NPs. (B) Selectivity of the preirradiated **BDP-4/Cur-CL** NPs toward different RONS (200 μM) treatments. Inset: the afterglow luminescence images acquired by an IVIS imaging system under bioluminescence mode with an open filter. *p < 0.05, **p < 0.01, and ***p < 0.001 vs PBS-treated group. (C) Afterglow luminescence spectra of **BDP-4/Cur-CL** NPs in the absence or presence of ONOO^–^ (200 μM) in PBS solution (pH 7.4). Insets: the corresponding afterglow luminescence acquired on an IVIS imaging system. (D) Tissue penetration depths of the activated afterglow luminescence (AF) of preirradiated and ONOO^–^-added NPs and the NIR fluorescence (FL) from **BDP-4/Cur-CL** NPs excited at 535 nm (in PBS at pH 7.4) with a coverage of chicken breast tissues with different thicknesses. (E) Kinetics of curcumin release from **BDP-4/Cur-CL** NPs with or without the addition of ONOO^–^ (200 μM). The released curcumin was determined by UPLC.

The morphology, size, and stability of the NPs were evaluated. Transmission electron microscopy (TEM) images demonstrated that **BDP-1/Cur-CL** NPs, **BDP-2/Cur-CL** NPs, **BDP-3/Cur-CL** NPs, **BDP-4/Cur-CL** NPs, curcumin NPs, and **Cur-CL** NPs were all spherical with uniform sizes of 106, 92, 110, 98, 58, and 69 nm, respectively (Figures 3A and S40, inset). Dynamic light scattering (DLS) showed that their hydrodynamic sizes are 167, 145, 152, 162, 92, and 103 nm, respectively (Figures S39 and S40). The sizes observed by DLS are larger due to a hydrated surface layer on the NPs. The diameters of all of the NPs remain almost unchanged after 14 days in water (Figure 3A), indicating robust colloidal stability. The spherical morphology, appropriate size, and outstanding stability suggest that these NPs are suitable for transcellular transport to neurons.

The **BDP-4/Cur-CL** NPs were selected to construct an NIR afterglow image-guided drug delivery system because they have the most favorable NIR AIE emission and highest ^1^O_2_ generation ability. A series of reactive oxygen and nitrogen species (RONS), namely, NO_2_^–^, *t*-Bu-hydroperoxide (TBHP), OH^–^, O_2_^•–^, ^1^O_2_, H_2_O_2_, ClO^–^, and ONOO^–^, were used to study the selectivity of **BDP-4/Cur-CL** NPs (Figure 3B). Intriguingly, the bright afterglow signal was captured in the presence of ONOO^–^, verifying the very selective recognition with ONOO^–^. The signal intensity enhanced ≥732-fold compared to phosphate-buffered saline (PBS). Under physiological conditions (pH range of 6.0–7.4), only the afterglow signal of ONOO^–^ activation was observed (Figure S42), while the H_2_O_2_-activated afterglow signal was observed only at pH 8.8 (outside the pathological range), proving the high specificity of the **BDP-4/Cur-CL** NPs toward ONOO^–^. The ONOO^–^-activated afterglow signal is pH-dependent (Figure S43), with a decreased intensity accompanying a decrease in the pH value. The **BDP-4/Cur-CL** NPs exhibited afterglow spectra similar to their fluorescence. The afterglow peaked at λ_max_ 697 nm (Figure 3C), and no luminescence peak was observed from **AGL** (structure shown in Figure 1B). The energy-transfer efficiency between **AGL** and **BDP-4** was calculated to be 94.6% by comparing the afterglow intensity in the presence and absence of **BDP-4** (Figure S44). A negligible change in the fluorescence spectrum of the **BDP-4/Cur-CL** NPs was observed in the presence of ONOO^–^, confirming the high tolerance of the NPs to ONOO^–^ (Figure S45). As shown in Figure S46A, the activated afterglow signal of **BDP-4/Cur-CL** NPs is higher than those of **BDP-1/Cur-CL** NPs, **BDP-2/Cur-CL** NPs, and **BDP-3/Cur-CL** NPs, which can be attributed to the more enhanced NIR AIE emission and higher ^1^O_2_ generation ability of **BDP-4**. The optimized NPs with a **BDP-4/Cur-CL** molar ratio of 1:2 and a white-light (0.25 W cm^–2^) preirradiation time of 10 min produced the best afterglow signal (Figures S47 and S48).

The kinetics of chemiluminescence (CL) of the **BDP-4/Cur-CL** NPs was further studied. After the addition of ONOO^–^ (200 μM), the afterglow intensity initially increased, reaching a maximum within 35 min, and then gradually decreased (Figure S46B). The half-life of the afterglow luminescence was estimated to be 98.6 min, which provides sufficient time to monitor the drug release. Most importantly, the afterglow intensity of the **BDP-4/Cur-CL** NPs showed a linear response with increasing ONOO^–^ concentration, and the limit of detection (LOD) was calculated to be 82.67 nM (Figure S46C). The low LOD predicts high sensitivity and excellent specificity for ONOO^–^, facilitating clear visualization of ONOO^–^ levels at the infarct site *in vivo*.

It is well-known that deep tissue penetration is essential to ensuring the application of NPs for imaging in the brain. As shown in Figure 3D, the NIR luminescence intensity obviously decreased with increasing thickness of chicken tissue. It is noteworthy that the afterglow intensity is still clearly observable in tissues up to 20 mm thick, while the NIR fluorescence signal is barely detectable in tissues up to 10 mm thick, which is attributed to the increased tissue penetration of the afterglow imaging modality without external light excitation. The diameters of preirradiated **BDP-4/Cur-CL** NPs in the PBS solution remained almost unchanged after 7 days (Figure S49A), indicating excellent colloidal stability. There were no obvious changes of the fluorescence intensity and afterglow luminescence for these preirradiated **BDP-4/Cur-CL** NPs during 7 days (Figures S49B and S50). These results indicate that the preirradiated **BDP-4/Cur-CL** NPs have high optical stability, which is beneficial for future commercial applications.

To analyze the drug release kinetics of **BDP-4/Cur-CL NPs**, liquid chromatograph–mass spectrometry (LC–MS) data were obtained. As shown in Figure S51, preirradiation caused a slight shift in the retention time due to the conversion of the **BDP-4/Cur-CL** NPs to **CL-1** (Figure 1B).^26^ After ONOO^–^ treatment, the peak at 7.5 min was replaced by a new peak at 4.9 min, signaling the release of curcumin. The **BDP-4/Cur-CL** NPs showed a progressive curcumin release after ONOO^–^ treatment and reached a plateau at 29.5 h with a release rate of 86% (Figure 3E). As expected, there was negligible curcumin release in the absence of ONOO^–^, demonstrating that drug release is triggered at an infarct site only when ONOO^–^ is abundant.

### Cellular Evaluation of Nanoparticles

**Figure 4:**
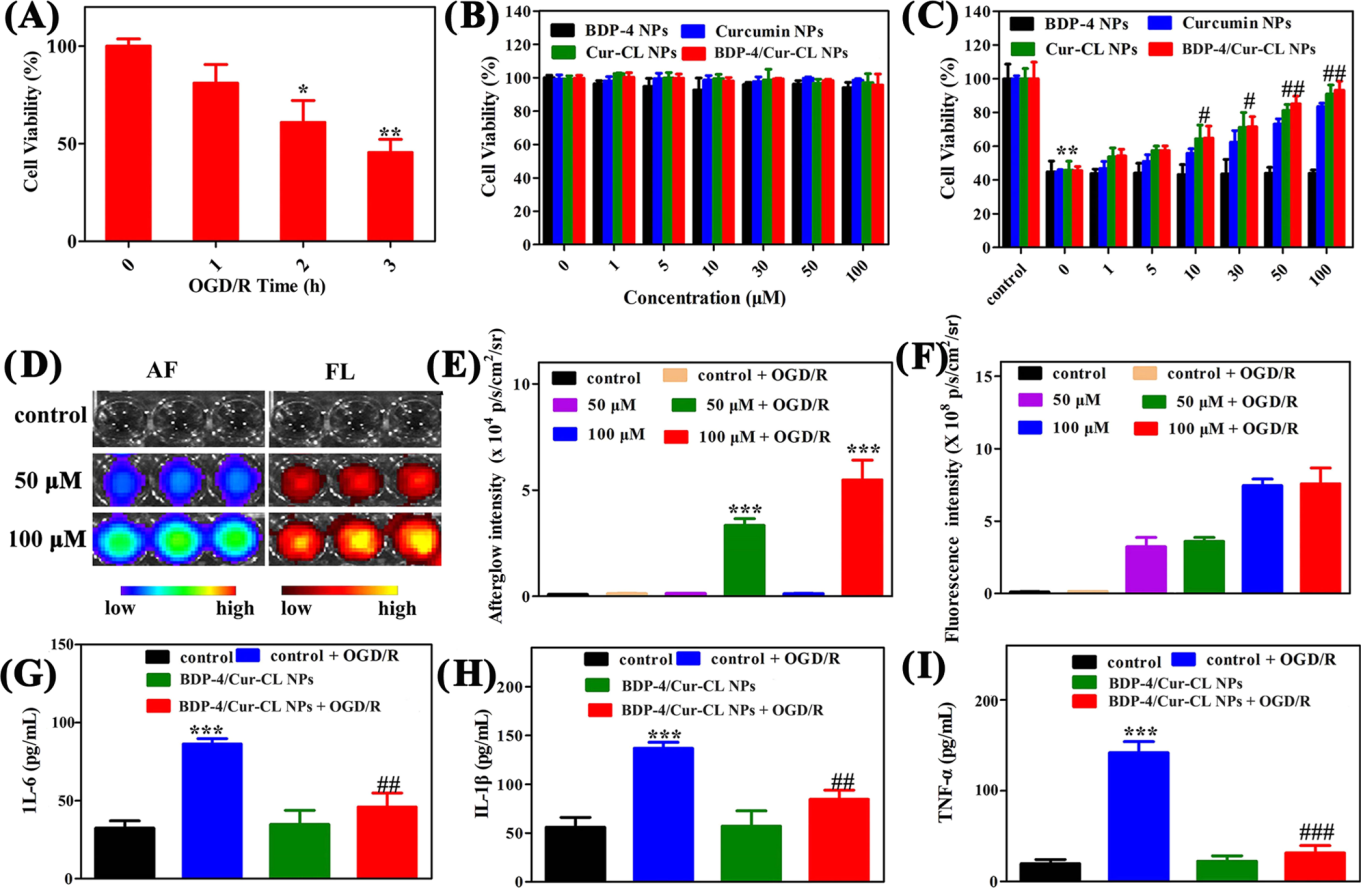
(A) Cell viability was measured after mice HT22 cells were exposed to the OGD at different times. (B) Injury in HT22 cells of different NPs in normal conditions. (C) Injury in HT22 cells of different NPs exposed to OGD/R conditions. (D) Afterglow and fluorescence cell imaging of **BDP-4/Cur-CL** NPs when present in culture with the corresponding OGD/R conditions. (E) Afterglow and (F) fluorescence intensities of the preirradiated **BDP-4/Cur-CL** NPs in different conditions. **BDP-4/Cur-CL** NPs decrease the level of expression of inflammatory factors induced by the OGD/R conditions. Concentrations of (G) IL-6, (H) IL-1β, and (I) TNF-α were determined by an enzyme-linked immunosorbent assay (ELISA). *p < 0.05, **p < 0.01, and ***p < 0.001 vs control group. ^#^*p* < 0.05, ^##^*p* < 0.01, and ^###^*p* < 0.001 vs OGD/R-treated group.

To assess the neuroprotection *in vitro*, the cytotoxicity of **BDP-4** NPs, curcumin NPs, **Cur-CL** NPs, and **BDP-4/Cur-CL** NPs were measured toward mice HT22 cells by CCK-8 analysis.^51^ After 3 h of oxygen-glucose deprivation (OGD) plus 24 h of reoxygenation (OGD/R) treatment, the OGD/R treatment caused approximately 40% cell death (Figure 4A). The cell viability remained ≥95% after incubation of healthy HT22 cells with **BDP-4** NPs, curcumin NPs, **Cur-CL** NPs, and **BDP-4/Cur-CL** NPs (0–100 μM) for 24 h (Figure 4B), indicating good cytocompatibility. Correspondingly, the pretreatment with curcumin NPs, **Cur-CL** NPs, and **BDP-4/Cur-CL** NPs significantly increased the OGD/R-induced cell viability, implying effective dose-dependent protection (Figure 4C). Obviously, the **Cur-CL** NPs and **BDP-4/Cur-CL** NPs exhibit better protection than the curcumin NPs. As illustrated in Figures 4D–F and S52, in the presence of **BDP-4/Cur-CL** NPs, bright afterglow luminescence of the OGD/R-induced cell was observed, in contrast to negligible emission in healthy HT22 cells, confirming the outstanding sensitivity induced by the **BDP-4/Cur-CL** NPs.

The cellular uptake of **BDP-4** and **BDP-4/Cur-CL** NPs in HT22 cells was examined by confocal laser scanning microscopy (CLSM) (Figure S53). Bright fluorescence was observed, suggesting that **BDP-4** and **BDP-4/Cur-CL** NPs can be internalized by living cells. Cerebral I/R injury progression is accompanied by a robust inflammatory response;^44^ therefore, treatment with the **BDP-4/Cur-CL** NPs was performed to verify the effect on the key inflammatory factors *in vitro*. First, the expression of inflammatory factors IL-1β, IL-6, and TNFα was studied after the injection of **BDP-4/Cur-CL** NPs (50 μg mL^–1^) into cells for 24 h. The results showed that a strong inflammatory response was induced by OGD/R. In addition, **BDP-4/Cur-CL** NPs exhibit an almost negligible effect on the expression of the levels of IL-1β, IL-6, and TNFα, while, after OGD/R stimulation, the levels of IL-1β, IL-6, and TNFα were obviously reduced with the preincubation of **BDP-4/Cur-CL** NPs (Figure 4G–I), indicating that the **BDP-4/Cur-CL** NPs can significantly decrease the inflammatory responses of OGD/R-induced cells *in vitro*. In consideration of the RONS, ^•^NO, ONOO^–^, and O_2_^•–^ are key pathogenic species in IS. Therefore, the intracellular levels of O_2_^•–^ were evaluated by dihydroethidium (DHE), ^•^NO was measured by 4-amino-5-methylamino-2′,7′-fluorescein diacetate (DAF-FM DA) reagent, and ONOO^–^ was detected by hydroxyphenyl fluorescin (HPF). As expected, compared to the control group, the **BDP-4/Cur-CL** NPs significantly decreased the intracellular levels of ^•^NO, O_2_^•–^, and ONOO^–^*in vitro* in a dose-dependent manner (Figure S54). Hence, the **BDP-4/Cur-CL** NPs show excellent catalytic activity to scavenge the RONS, ^•^NO, ONOO^–^, and O_2_^•–^, which are believed to mediate I/R injuries.

### Real-Time Monitoring of Early Ischemic Stroke Process *In Vivo*

**Figure 5:**
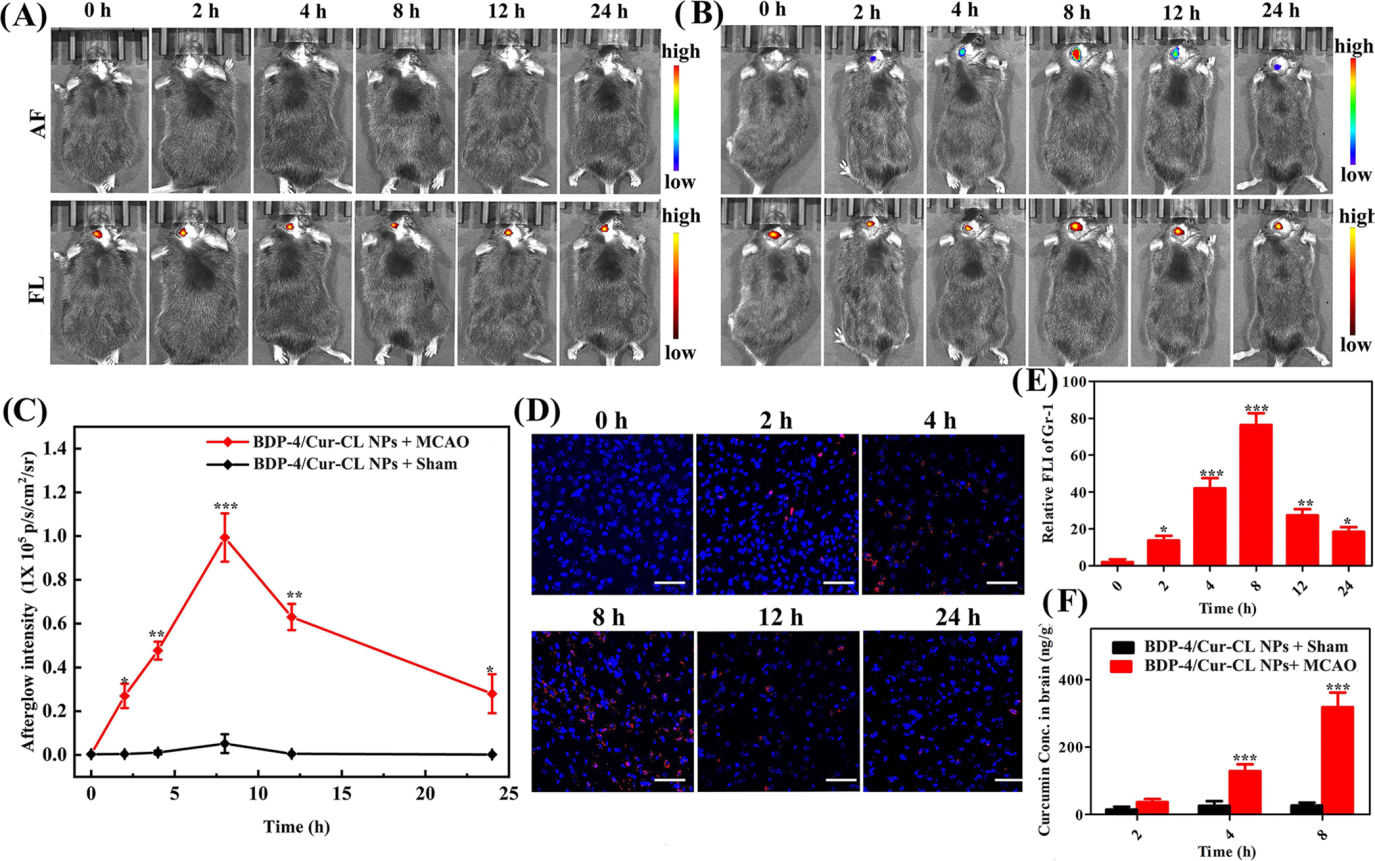
Representative time-dependent afterglow and fluorescence images for (A) sham and (B) IS models. (C) Quantitative average afterglow intensities for different groups of mice after injection of the preirradiated **BDP-4/Cur-CL** NPs. (D) Confocal images showing neutrophil infiltration in MCAO brain tissues at various times post light irradiation. The red signal represents neutrophils (Gr-1 staining), and the blue signal represents cell nuclei stained by 4,6-diamidino-2-phenylindole (DAPI). Scale bars, 50 μm. (E) Quantitative analysis of red fluorescence intensity (FLI) based on these images in panel (D). (F) The released curcumin amounts determined by UPLC-MS in brain tissues for different groups. Error bars, mean ± SD (*n* = 6). *p < 0.05, **p < 0.01, and ***p < 0.001 vs control group. ^#^*p* < 0.05, ^##^*p* < 0.01, and ^###^*p* < 0.001 vs PBS-treated group.

Motivated by the superior performance *in vitro*, **BDP-4/Cur-CL** NPs were further evaluated *in vivo* with an intraluminal MCAO mice model (an ischemic model).^52^ To monitor the cerebral blood flow for determining the stability of the model, laser speckle contrast imaging was performed (Figure S55). Subsequently, **BDP-4/Cur-CL** NPs were used to study the evolution process of IS and the underlying operational principle. The mice were first subjected to cerebral ischemia for 30 min followed by reperfusion for 30 min and then treated with preirradiated **BDP-4/Cur-CL** NPs (5 mg mL^–1^, 10 μL). As a control, the sham group was treated with preirradiated **BDP-4/Cur-CL** NPs (Figure 5A). As shown in Figure 5B, the turn-on afterglow signal at the ischemia reperfusion injury site was significantly observed, and negligible afterglow was observed in the control sham groups, indicating that the **BDP-4/Cur-CL** NPs can successfully detect the signal for the ONOO^–^ output in the mouse brain during I/R injury. As expected, the real-time activated afterglow luminescence intensity increased to a peak at 8 h post injection, followed by a steady decrease from 8 to 24 h, reflecting the intense evolution of ONOO^–^ during the process of IS (Figure 5C). However, with the “always-on” fluorescent nanoprobe technique, it was very difficult to distinguish the negligible increase in fluorescent signals for both the control sham and the I/R injury groups with the same injection of **BDP-4/Cur-CL** NPs (Figure S56). These results suggest that the targeted and activatable afterglow luminescence process has high sensitivity for detecting the IS, rather than the “always-on” fluorescent nanoprobe with an indistinguishable signal between the normal and injured regions.

Neutrophils are a type of white blood cell that plays a key role as a marker to judge the degree of inflammation for injury following IS.^53^ As shown in Figure 5D,E, the increase in the level of infiltrated neutrophils was in line with the afterglow luminescence intensity, which also reached a peak at 8 h, indicating that the ONOO^–^-activated NIR afterglow signals of **BDP-4/Cur-CL** NPs could serve to monitor the level of neutrophils. As anticipated, the increase in curcumin release was consistent with the time of the activated NIR afterglow signal. Also, the amount of curcumin for the MCAO model (318.3 ng g^–1^) is much higher than for the sham model (29.1 ng g^–1^) at 8 h post light irradiation (Figure 5F). The data suggest that **BDP-4/Cur-CL** NPs can effectively probe the curcumin release status in IS *in vivo*. These results strongly validate that the **BDP-4/Cur-CL** NPs can be successfully used for ONOO^–^ detection in the early phase of IS, showing great value for monitoring the progress of IS.

### BDP-4/Cur-CL NPs Provide Cerebral Protective Effect *In Vivo*

**Figure 6:**
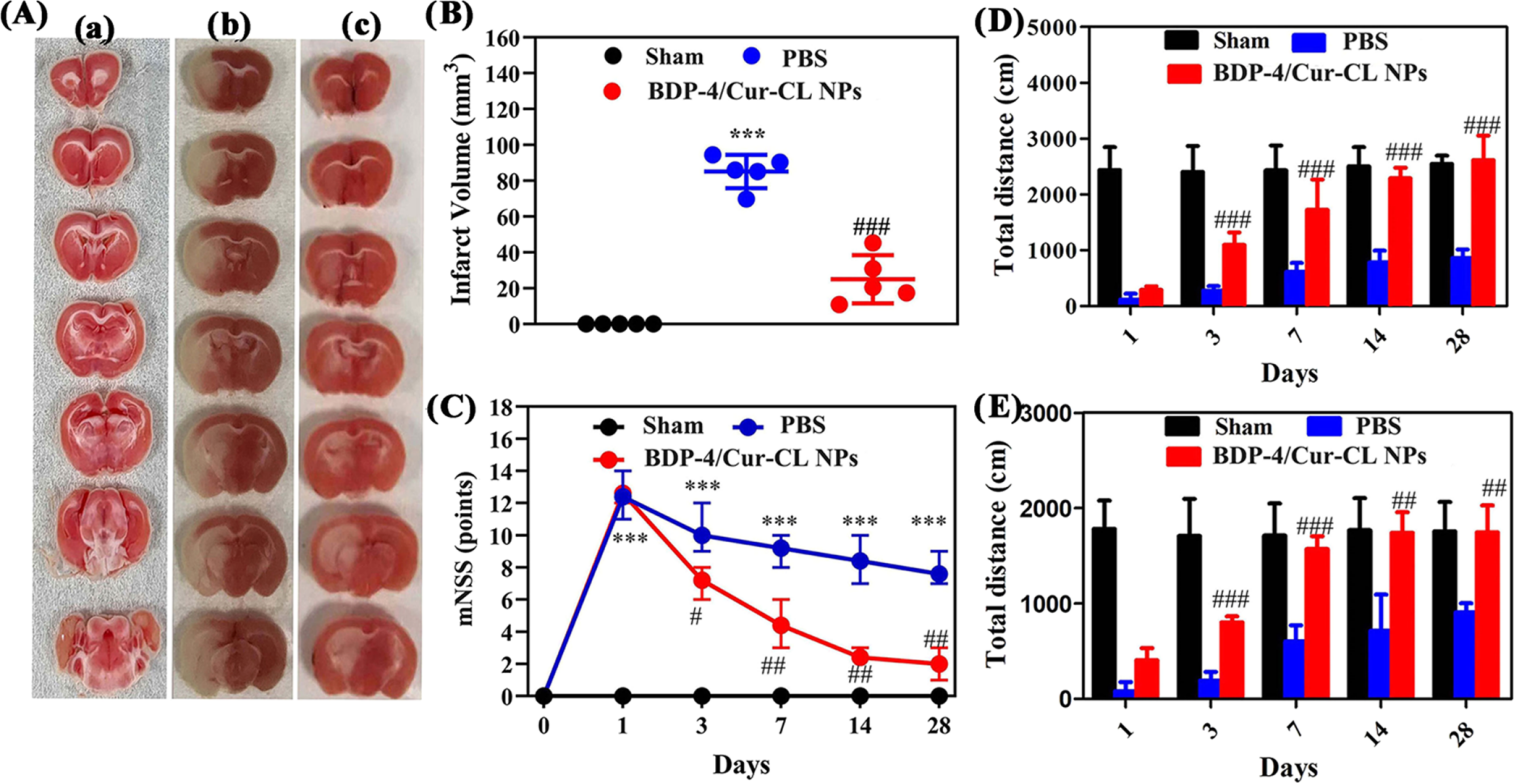
(A) 2,3,5-Triphenyltetrazolium chloride (TTC) staining for sham and IS groups. (B) Infarct volume for sham and IS groups. (C) Neurological scores for sham and IS groups (*N* = 10–12). The total distance of (D) open field and (E) elevated plus-maize test for different groups at 1, 3, 7, 14, and 28 days. *p < 0.05, **p < 0.01, and ***p < 0.001 vs control group. ^#^*p* < 0.05, ^##^*p* < 0.01, and ^###^*p* < 0.001 vs PBS-treated groups.

Furthermore, the cerebral protective effect of **BDP-4/Cur-CL** NPs was evaluated *in vivo*. The infarct volumes were observed at 72 h post stroke after treatment. As indicated in Figure 6A,B, compared with the group treated with PBS (90.49 mm^3^), the infarct volumes were significantly reduced for the group treated with **BDP-4/Cur-CL** NPs (12.97 mm^3^), implying significant efficacy in decreasing brain injury. To evaluate the effect of **BDP-4/Cur-CL** NPs on neurological recovery at the indicated time points, the modified neurological severity score (mNSS) with a range of 0–18 is shown (Figure 6C). Low mNSS represents a milder neurological deficit and better effective neuroprotective function. Importantly, the score of the **BDP-4/Cur-CL** NP-treated group was significantly lower than that of the PBS-treated group at all time points, revealing that the **BDP-4/Cur-CL** NPs possess strong protection against ischemic injury *in vivo*. The behavioral changes were evaluated by the open field (Figures 6D and S58) and the elevated plus-maize tests (Figures 6E and S59). Compared to the sham groups, the PBS-treated group exhibits lesser total distance and slower moving speed, indicating severely impaired motion due to the stroke.^8^ As expected, compared to the PBS-treated group, larger total distances and faster moving speeds were achieved by the **BDP-4/Cur-CL** NP group, showing a significant neuroprotective effect. The survival rate of the **BDP-4/Cur-CL** NP group was higher than that of the PBS-treated group (Figure S60A). Moreover, the **BDP-4/Cur-CL** NP treatment for 4 weeks had no effect on the mice body weight (Figure S60B).

Brain sections were collected after 72 h of MCAO. Notably, the **BDP-4/Cur-CL** NPs were found in neurons, indicating an active endocytosis of the **BDP-4/Cur-CL** NPs (Figure S61). The **BDP-4/Cur-CL** NP group had a significantly better functional outcome than the other groups, proving that the **BDP-4/Cur-CL** NPs protected against ischemic injury *in vivo*. As shown in Figure S62, the fluorescence intensity of **BDP-4/Cur-CL** NPs from the brain in MCAO stroke mice gradually decreased within 14 days, indicating that NPs can be metabolized in the brain.

### Safety Evaluations of BDP-4/Cur-CL NPs

To investigate the cytotoxicity and biosafety aspects, the **BDP-4/Cur-CL** NPs were further evaluated *in vivo* by serum biochemical detection and hematoxylin and eosin (H&E) staining of major organs at 3 and 28 days. No significant difference was observed between the untreated mice and the mice injected with **BDP-4/Cur-CL** NPs regarding the biochemical analysis of blood (Figures S63 and S64), demonstrating the favorable biocompatibility of the **BDP-4/Cur-CL** NPs. As presented in Figure S65, no pathological changes were found in the major organs of the six groups of mice (namely, with sham for 3 days; with MCAO for 3 days; with MCAO + **BDP-4/Cur-CL** NPs for 3 days; with sham for 28 days; with MCAO for 28 days; with MCAO + **BDP-4/Cur-CL** NPs for 28 days of sham). Also, negligible cell necrosis was observed in the brains treated with **BDP-4/Cur-CL** NPs, further indicating a positive therapeutic outcome.

## Conclusions

In summary, we have successfully constructed ONOO^–^-activated **BDP-4/Cur-CL** NPs with early diagnosis, real-time reporting, drug tracing, and treatment functions for IS. These outputs do not occur in the absence of ONOO^–^, whereas there is significant brightness (maximum 732-fold increase) and curcumin release rate (86%) enhancement with ONOO^–^ stimulation and dependent upon the concentration of endogenous ONOO^–^. Notably, the **BDP-4/Cur-CL** NPs possess bright NIR emission at 697 nm, superb sensitivity (LOD = 82.67 nM), deep tissue penetration (20 mm), high energy-transfer efficiency (94.6%), and outstanding antiapoptosis and anti-inflammation effects. The activated NIR afterglow signal obtained in *in vivo* experiments showed three functions: (i) instantly and accurately distinguishing between I/R injury and normal areas for endogenous ONOO^–^ detection in the early stage of IS; (ii) real-time reporting of endogenous ONOO^–^ levels in the lesion area for monitoring the evolutionary mechanisms of IS; and (iii) dynamic monitoring of the release status of curcumin. The released curcumin effectively decreased apoptosis, enhanced survival rates, alleviated neuroinflammation, reduced brain tissue loss, and improved the cognition of MCAO stroke mice.

The special highlights of the **BDP-4/Cur-CL** NPs are as follows: (1) this work is the first example of NIR afterglow NPs applied to IS. The NPs successfully overcome the inherent limitation of conventional fluorescent materials that rely on real-time excitation;^16,19−24^ they possess higher tissue penetration (20 mm), which ensures excellent imaging performance in the brain. (2) This work represents one of the very few examples of optical agents that distinguish I/R damage from normal areas, enabling ONOO^–^ detection in the early stage of IS.^31−34^ (3) More importantly, the ability to visualize the dynamic fluctuations of endogenous ONOO^–^ levels in the lesion area is valuable for real-time monitoring of the development and evolution of the mechanisms of IS. (4) Compared to current activatable image-guided drug delivery systems,^11,17,18^ the **BDP-4/Cur-CL** NPs enable dynamic monitoring of curcumin release for safe treatment. (5) Overall, the **BDP-4/Cur-CL** NPs were successfully applied to IS with the combined desirable features of early diagnosis, real-time reporting, drug tracking, therapeutic functions, and monitoring of drug release. Thus, this work provides new opportunities for the development of therapeutic agents for stroke treatment based on NIR afterglow luminescence for personalized IS therapy.

## Experimental Section

### Materials

All of the chemicals were obtained from Sigma-Aldrich unless otherwise specified. The solvents for chemical reactions were distilled before use. 1,2-Distearoyl-*sn*-glycero-3-phosphoethanolamine-*N*-[maleimide(poly(ethylene glycol))_−2000_] (DSPE-PEG-MAL) and sulfhydrylated lactoferrin (Lf-SH) were purchased from Laysan Bio, Inc. (Arab, AL). Milli-Q water was supplied by a Milli-Q Plus System (Millipore Corporation, Bedford). RPMI 1640 culture medium, penicillin–streptomycin, and fetal bovine serum (FBS) were purchased from Gibco-BRL (Grand Island, NY). Primary antibodies and fluorescent secondary antibodies for CLSM imaging were provided by Abcam and Invitrogen, respectively. Enzyme-linked immunosorbent assay (ELISA) kits were provided by Biolegend.

### Statistical Analysis

All data are expressed as mean ± standard deviation (SD). GraphPad Prism 8 (GraphPad Software Inc., San Diego, CA) was used for statistical analysis. The Brown–Forsythe test was used to test the homogeneity of the variance. *P* < 0.05 was considered statistically significant. The specific characterization and experimental methods are reported in the Supporting Information.

## Supporting Information Available

Additional experimental details and data, including synthetic procedures (Schemes S1 and S2), ^1^H NMR (Figures S1–S16),^13^C NMR spectroscopy (Figures S17–S20), mass spectrometry (Figures S21–S24), optimized molecular geometry (Figure S25), photophysical data (Figures S26–S32), ROS generation ability of the AIEgens NPs (Figures S33–S38), DLS results (Figures S39 and S40), ζ-potential results (Figure S41), characterization of ONOO^–^-activatable NIR afterglow luminescent nanoprobes (Figures S42–S51), afterglow and fluorescence luminescence cell imaging (Figures S52–S54), laser speckle contrast images for CBF in MCAO stroke (Figure S55), fluorescent intensities of mice (Figures S56 and S57), open field test results (Figure S58), elevated plus-maize test results (Figure S59), survival rate and body weights (Figure S60), targeting of **BDP-4/Cur-CL** NPs in ischemic stroke results (Figure S61), the fluorescence imaging of mouse organs (Figure S62), blood test (Figures S63 and S64), and H&E staining (Figure S65) (PDF)
