# Supplementary material for: Near-Infrared Afterglow ONOO–-Triggered Nanoparticles for Real-Time Monitoring and Treatment of Early Ischemic Stroke
Source: ACS Appl Mater Interfaces. 2023 Sep 20;15(39):45574–84. doi: 10.1021/acsami.3c08033 (PMC10561133; doi:10.1021/acsami.3c08033)
Supplement: Supplementary file 1 — am3c08033_si_001.pdf [file am3c08033_si_001.pdf]

Supporting Information

Near-infrared Afterglow $ONOO^-$ Triggered Nanoparticles for Real-time Monitoring and Treatment of Early Ischemic Stroke

Liping Zhang[a§], Ya-chao Wang[a§], Yuqi Liao[a§], Qian Zhang[a], Xia Liu[a], Dongxia Zhu[b], Haixing Feng[a], Martin R. Bryce[c*], and Lijie Ren[a*]*

[a] Dr. L. Zhang, Dr. Y. Wang, Dr. Y. Liao, Dr. Q. Zhang, Dr. X. Liu, Dr. H. Feng, Prof. L. Ren, Department of Neurology, Inst Translat Med, The First Affiliated Hospital of Shenzhen University, Shenzhen Second People's Hospital, Shenzhen 518035.

[b] Prof. D. Zhu, Key Laboratory of Nanobiosensing and Nanobioanalysis at Universities of Jilin Province, Department of Chemistry Northeast Normal University, 5268 Renmin Street, Changchun, Jilin Province 130024, P. R. China.

[c] Prof. M. R. Bryce, Department of Chemistry Durham University Durham, DH1 3LE, UK.

E-mails: renlijie72@126.com; zhudx047@nenu.edu.cn; m.r.bryce@durham.ac.uk

§ L. Z., Y. W. and Y. L. contributed equally to this work.

Experimental Procedures

Materials. All the chemicals were obtained from Sigma-Aldrich unless otherwise specified. The solvents for chemical reactions were distilled before use. 1,2-distearoyl-*sn*-glycero-3-phosphoethanolamine-*N*-[maleimide(poly(ethyleneglycol))$_{-2000}$] (DSPE-PEG-MAL) and sulfhydrylated lactoferrin (Lf-SH) were purchased from Laysan Bio, Inc. (Arab, AL). Milli-Q water was supplied by Milli-Q Plus System (Millipore Corporation, Breford, USA). RPMI 1640 culture medium, penicillin-streptomycin, and fetal bovine serum (FBS) were purchased from Gibco-BRL (Grand Island, NY, USA). Primary antibodies and fluorescent secondary antibodies for CLSM imaging were provided by Abcam and Invitrogen, respectively. Enzyme-linked immunosorbent assay (ELISA) kit was provided by Biolegend.

Characterization. ^{1}H and ^{13}C NMR spectra were recorded on a Bruker-DPX 400 spectrometer. Chemical shifts are reported in ppm relative to tetramethylsilane (TMS) with the solvent resonance as the internal standard (δ 0.00 ppm). High-resolution mass spectra (HRMS) were recorded on a Varian 7.0T FTMS Mass Spectrometer System operating in Matrix-Assisted Laser Desorption/Ionization Time of Flight (MALDI-TOF) mode. The HPLC-MS analysis was conducted on ACQUITY HPLC I-Class/UPCC/M Class/SYNAPT G2-SI instruments. UV-vis absorption spectra were recorded on a Thermo GENESYS 50 spectrometer. Photoluminescence was recorded on a Perkin-Elmer LS 55 spectrofluorometer. The nanoparticles' morphology was investigated using transmission electron microscopy (TEM, JEM-2010FJEOL, Japan). Size distribution of the NPs was measured using dynamic light scattering (DLS) with a Malvern Nano-Zetasizer instrument.

Computational Details. All density functional theory (DFT) calculations were performed using Gaussian 09.[1] The geometries and frequency calculations were performed using the B3LYP

density functional in conjunction with the 6-31G(d) basis set. Frequency calculations confirmed that the optimized structures are energy minima (no imaginary frequencies). The molecular orbital information including energy levels and distribution was obtained at the same theoretical level.

Preparation of Nanoparticles. Synthesis of **BDP-1** NPs, **BDP-2** NPs, **BDP-3** NPs, **BDP-4** NPs, **Cur-CL** NPs and Curcumin NPs: A mixture of **BDP-1**, **BDP-2**, **BDP-3**, **BDP-4**, **Cur-CL** or Curcumin (1 mg) and DSPE-PEG-MAL (2 mg) in THF (1 ml) was sonicated for 1 min. The mixture was then poured into water (10 mL). The solution was stirred at room temperature to evaporate THF. Subsequently, sulfhydrylated lactoferrin (Lf-SH) (500 μg/mL, 150 μL) was added into the above solution and stirred at room temperature for 12 h.

Preparation of $ONOO^-$ Activated NIR Afterglow Protheranostic Nanoparticles. Synthesis of **BDP-1/Cur-CL** NPs, **BDP-2/Cur-CL** NPs, **BDP-3/Cur-CLBDP-3/Cur-CL** NPs and **BDP-4/Cur-CL** NPs: A mixture of **BDP-1**, **BDP-2**, **BDP-3** or **BDP-4** (0.5 mg), **Cur-CL** (1.0 mg) and DSPE-PEG-MAL (3 mg) in THF (1 ml) was sonicated for 1 min. The mixture was then poured into water (10 mL). The solution was stirred at room temperature to evaporate THF. Subsequently, Lf-SH (500 μg/mL, 150 μL) was added into the above solution and stirred at room temperature for 12 h.

Superoxide anion radical ($O_2^{\bullet-}$) detection. An aqueous solution containing dihydrorhodamine 123 (DHR123) (10^{-5} M) was mixed with **BDP-1** NPs, **BDP-2** NPs, **BDP-3** NPs or **BDP-4** NPs (10^{-5} M). The solution was irradiated under 20 mW cm^{-2} white light for 6 min with a time interval of 1 min. The DHR123 fluorescence signal at 526 nm was continuously monitored at predesignated irradiation times. The generation of $O_2^{\bullet-}$ was indirectly characterized by the ratio of the enhancement of DHR123 fluorescence intensity after light irradiation.

Hydroxyl radical (•OH) **detection.** An aqueous solution containing hydroxyphenyl fluorescein (HPF) (10^{-5} M) was mixed with **BDP-1** NPs, **BDP-2** NPs, **BDP-3** NPs or **BDP-4** NPs (10^{-5} M). The solution was irradiated under 20 mW cm^{-2} white light for 9 min with a time interval of 3 min. The HPF fluorescence signal at 516 nm was continuously monitored at predesignated irradiation times. The generation of •OH was indirectly characterized by the ratio of the enhancement of HPF fluorescence intensity after light irradiation.

Preparation of Reactive Oxygen and Nitrogen Species (RONS) Solutions. The RONS solutions containing H_2O_2, TBHP and ClO^-, respectively, were purchased and diluted to the experimental concentration (200 μM) by 1×PBS (pH 7.4).[2] In brief, •OH was produced by addition of ferrous chloride (0.1 M, 1 mL) into H_2O_2 solution (1.0 M, 1 mL) through a Fenton reaction. Accordingly, the concentration of •OH is the same as that of Fe^{2+} (50 mM). $O_2^{\bullet-}$ was generated from KO_2 (35.5 mg), which was directly added into dimethyl sulfoxide (10 mL) at a final concentration of 50 mM. $ONOO^-$ was prepared by addition of sodium hydroxide (1.5 M) into mixtures of sodium nitrite (0.6 M), hydrogen peroxide (0.7 M) and hydrochloric acid (0.6 M) at 0 °C, followed by purification through a short column of manganese dioxide to remove excess hydrogen peroxide. The concentration of $ONOO^-$ was determined by measurement of the absorption at 302 nm. $C[ONOO^-] = Abs_{302nm}/1.67$ (mM). $^1O_2$ was produced by addition of ClO^- solution (100 mM, 1 mL) into H_2O_2 solution (200 mM, 1 mL). According to this reaction, the concentration of $^1O_2$ is the same to that of ClO^- (50 mM). NO_2^- was generated from $NaNO_2$ (35 mg), which was directly added into deionized water (10 mL) at a final concentration of 50 mM.

Afterglow and Fluorescence Imaging *In Vitro*. For *in vitro* afterglow imaging, including determinations of RONS selectivity, afterglow luminescence spectra, and $ONOO^-$ activated sensitivity, etc., the concentration of **BDP-4/Cur-CL** NPs was 100 μg/mL (based on **Cur-CL**).

Before use, **BDP-4/Cur-CL** NPs were pre-irradiated by white light (0.25 W cm^{-2}) for 10 min. The afterglow imaging was carried out with an Xenogen IVIS Lumina II system in a bioluminescent mode (exposure time for 60 s) post RONS (200 μM) addition, unless otherwise specified. Tests were every 20 nm/step by the different filters. The environmental temperature for *in vitro* afterglow imaging was kept at 37 °C. The fluorescence spectra of **BDP-4/Cur-CL** NPs were measured by the same IVIS instrument in a fluorescent mode with excitation at 500 ± 10 nm and emission from 697 ± 10 nm (exposure time for 1 s). The fluorescence and afterglow luminescence images were analyzed by ROI analysis using the Living Image 4.2 Software.

Calculation of FRET efficiency. FRET efficiency was calculated according to the equation:

$$E := 1 - \frac{I}{I_0} \qquad (1)$$

where I and I_0 are the afterglow luminescence intensities of **Cur-CL** NPs and **BDP-4/Cur-CL** NPs, respectively.[3] To reduce the error, the afterglow luminescence intensities (≈ 550 nm) of **Cur-CL** NPs (1236.2, 1068.3 and 1147.9) and **BDP-4/Cur-CL** NPs (13.6, 73.4 and 94.35) were measured in three parallel experiments. According to the equation (1) the energy transfer efficiency between **AGL** (structure shown in manuscript Figure 1) and **BDP-4** in the presence of $ONOO^-$ was calculated to be 94.6%.

Curcumin Release Experiment *In Vitro*. $ONOO^-$ solution (200 μM) was added to **BDP-4/Cur-CL** NPs (100 μg/mL, based on **Cur-CL**) and held at 37 °C. 200 μL of the reaction solution was taken at certain intervals for UPLC analysis. The chromatographic separation was accomplished with the mobile phase consisting of acetonitrile and water (containing 0.05% trifluoroacetic acid, v/v) in gradient elution ranging from 70:30 to 95:5 (acetonitrile/water, v/v) for the first 8 minutes and isocratic elution (95:5) for the next 14 minutes. The flow rate of the mobile phase was set at 1.0 mL min^{-1} and the injection volume was 10 μL.

Cell Culture. The HT22 cells were regularly checked for mycoplasma contamination. HT22 cells were cultured in Dulbecco's Modified Eagle's Medium (DMEM) (Gibco®, Grand Island, NY, USA) supplemented with 10% fetal bovine serum (FBS) (Gibco®), penicillin (100 U/mL), and streptomycin (100 μg/mL) and the cultures were maintained at 37 °C in a humidified atmosphere containing 5% CO_2. An *in vitro* model OGD/R of ischemia–reperfusion was built on HT22 cells, derived from mouse hippocampal neurons.

***In vitro* model of OGD/R**. 100 μL of HT22 cell suspension were added into each well of a 96-well plate at a density of 5×10^4 cells mL^{-1}. Cells were cultured in a hypoxic incubator in glucose-free DMEM without FBS used as cell media for 1, 2, and 3 h at 37 °C to establish the optimal OGD time. Hypoxic conditions were 5% CO_2, 94% N_2, and 1% O_2. The HT22 cells were then reoxygenated under conditions of 95% air and 5% CO_2 for 24 h at 37 °C. Controls were cells that were not exposed to OGD/R. The CCK-8 solution (Kumamoto, Kyushu, Japan) was added for 2 h. The absorbance at 450 nm was recorded using a microplate reader (Multiskan GO 1510, USA). Measurements were done in triplicate.

Assessment of Cell Viability and Cytotoxicity. The complete culture medium for cell culture included basal DMEM high glucose, 10% FBS and 1% penicillin/streptomycin. The incubator environment was maintained at 37 °C, 95% air plus 5% CO_2. After incubating with different concentrations of NPs for 24 h, a CCK-8 assay was used to detect cell viability. When implementing the OGD/R model, the complete medium was first replaced with DMEM medium without D-glucose, and cells were transferred to a closed container filled with 5% CO_2, 94% N_2, and 1% O_2 for 3 h. Then the cells were placed in a normal culture environment, and the medium was replaced back to the complete medium. The control group was cultured normally under standard conditions.

Chemiluminescence imaging in living cells. HT22 cells were seeded in 96-well Corning clear bottom plates for 24 h before treatment. Then **BDP-4/Cur-CL** NPs (50 μM and 100 μM) were added into the cell culture medium separately. After exposure to OGD/R, the cell culture medium including the **BDP-4/Cur-CL** NPs was pre-irradiated by white light (0.25 W cm^{-2}) for 10 min. The afterglow imaging was carried out with an Xenogen IVIS Lumina II system in a bioluminescent mode (exposure time for 60 s) unless otherwise specified. The fluorescence spectra of **BDP-4/Cur-CL** NPs were measured by the same IVIS instrument in a fluorescent mode with excitation at 500 ± 10 nm and emission from 697 ± 10 nm (exposure time for 1 s). The control group was cultured normally under standard conditions. Experiments was performed in triplicate.

Cell uptake by confocal laser scanning microscopy (CLSM). HT22 cells were seeded in 6-well culture plates at a density of $5{\times}10^4$ cells per well for 24 h. Then the **BDP-4** NPs and **BDP-4/Cur-CL** NPs (20 μM) were added into the cell culture medium separately. They were incubated for 1 h, 2 h and 4 h. Subsequently, 4′,6-diamidino-2-phenylindole (DAPI) was used to stain the cell nuclei. Finally, the outcomes were observed by CLSM with excitation at 488 nm.

Enzyme-Linked Immunosorbent Assay. The supernatants were collected from the different treatment groups of HT22 cells, and the concentrations of the inflammatory factors, namely brain necrosis factor (TNF)-α, interleukin (IL)-1β, and IL-6 were measured using an enzyme-linked immunosorbent assay (ELISA) kit. Each experiment was performed independently three times.

***In Vitro* Intracellular ROS and RONS Evaluation**. The reactive oxygen species (ROS) and RONS were imaged using specific agents, namely, 2’7’-dichlorofluorescin diacetate (DCFH-DA) for ROS, dihydroethidium (DHE) for $O_2^{\bullet-}$, hydroxyphenyl fluorescin (HPF) for •OH/$ONOO^-$, and diaminofluorescin-FM diacetate (DAF-FM DA) for •NO. HT22 cells (5×10^5 cells/well) were pretreated with four concentrations of **BDP-4/Cur-CL** NPs for 24 h before exposure to OGD/R

for an additional 24 h, washed with phosphate buffered saline (PBS) three times, and then were incubated with DCFH-DA (10 μM), DHE (10 μM), DAF-FM DA (5 μM), and HPF (5 μM) for 30 min at 37 °C. Subsequently, residual agents were removed using PBS. The images were obtained using a confocal microscope (LSM 800 Zeiss, Germany). Each experiment was performed independently three times.

Animals and Group Assignments. Male C57 BL/6J mice (8-10 weeks old) were purchased from Beijing Vital River Laboratory Animal Technology Co., Ltd. The mice were maintained in a regular inverse 12 h:12 h light/dark cycle at temperatures between 20 and 23 °C and fed with a regular diet. All animal procedures were approved by the local government and the Ethics Committee of Shenzhen University. Animal experimental groups were assigned blindly and the mice focal ischemic stroke model was employed. The mice were blind to treatment with sham, PBS or **BDP-4/Cur-CL** NPs. Neurological scores were evaluated by Y.Q. Liao who was unaware of the experimental conditions. Stroke outcomes were unblinded after termination of the studies.

Focal Ischemic Stroke. Transient focal ischemic stroke was established in male mice as previously described. In general, the mice were anesthetized and the body temperature was kept at 37 °C using a heating pad control system (ALC-HTP homeothermic system, Alcott Biotech, Shanghai, China) during the whole surgery process. Middle cerebral artery regional cerebral blood flow (CBF) was monitored by laser Doppler flowmetry and a silicone-coated filament (RWD, Shenzhen, China) was inserted via the common carotid artery into the left internal carotid artery. CBF showed a reduction of >80% and flowed back to >70% after 30 min of reperfusion and hence the ischemic stroke model was set up. The filament was removed after 1 h occlusion and the skin incision was sutured. Mice were then returned to home cages. Mice with intracranial hemorrhage and those who did not meet the CBF requirements were excluded.

Laser Speckle Contrast Imaging. Mice (n = 8 for MCAO stroke) were subjected to 1-1.5% isoflurane. The hair was shaved, the skull was exposed, and CBF was detected using the laser speckle contrast imaging system (RWD, Shenzhen, China). The images and videos were analyzed using RWD software.

Drug-delivery process. Before use, **BDP-4/Cur-CL** NPs were pre-irradiated by white light (0.25 W cm^{-2}) for 10 min. Pre-irradiated **BDP-4/Cur-CL** NPs (5 mg mL^{-1}, 10 μL, based on curcumin) or saline (10 μL) preparation were administered into the striatum (+2.0 mm anterior/posterior, +1.0 mm medial/lateral, and +2.0/2.5 mm dorsal/ventral relative to bregma) using a stereotaxic device (RWD, Life Science, Shenzhen, China) and a Hamilton syringe (10 μL Hamilton, Cat. No.80300 USA).

***In vivo* imaging of BDP-4/Cur-CL NPs**. The mice were subjected to cerebral ischemia for 30 min followed by 30 min of reperfusion. Then pre-irradiated **BDP-4/Cur-CL** NPs (5 mg mL^{-1}, 10 μL, based on curcumin) were injected into the ischemic brain sites of the mice. 10 μL of either drug or 10 μL saline preparation were administered into the striatum (+2.0 mm anterior/posterior, +1.0 mm medial/lateral, and +2.0/2.5 mm dorsal/ventral relative to bregma) using a stereotaxic device (RWD, Life Science, Shenzhen, China) and a Hamilton syringe (10 μL Hamilton, Cat. No.80300 USA). Fluorescence imaging was taken at 0 h, 2 h, 4 h, 8 h, 12 h and 24 h post injection (exposure time for 1 s, excitation at 500 ± 10 nm and emission at 697 ± 10 nm). Afterglow images were acquired at 0 h, 2 h, 4 h, 8 h, 12 h and 24 h post irradiation (exposure time for 60 s, emission with an open filter).

The curcumin release in the ischemic stroke microenvironment. The mice in each group were sacrificed at 2 h, 4 h and 8 h after sham or MCAO, and 100 mg of brain tissue was dried on filter paper and cut into centrifuge tubes. PBS (500 μL) was then added and the sample was

homogenized using a Bioprep-24 Homogenizer. Next, methanol (1 mL) was added to the centrifuge tube and the tube was shaken on a shaker for 30 min to fully extract the curcumin from the brain tissue. The extracted tissue homogenate was centrifuged at 12,000 rpm for 15 minutes at 4 °C and the supernatant was collected. The methanol was evaporated using a SpeedVac Concentrator and the water was removed by a vacuum freeze dryer. The residue was dissolved in anhydrous methanol (1 mL) on an ultrasonic cleaner. The water-soluble protein, as well as other impurities or salts, were further removed by centrifuging at 12,000 rpm for 15 min. The supernatant was collected and passed through a 220 nm organic phase filtration membrane before further quantification of curcumin in each sample by UPLC-MS (Waters APGC/ UPLC Xevo TQ-S, ACQUITY UPLC BEH C18 column (1.7 µm, 2.1 mm × 100 mm) at 50 ˚C). The chromatographic separation was accomplished with the mobile phase consisting of acetonitrile and water (containing 0.1 % formic acid, v/v) in gradient elution ranging from 0-5 min 5:95 to 5-10 min 95:5 to 10-12 min 100:0 (acetonitrile/water, v/v). The flow rate of the mobile phase was set at 0.3 mL min^{-1} and the injection volumes were 10 µL. MS detector settings: *m/z* 234.7 at the voltage of 50 V and *m/z* 264.5 at the voltage of 28 V (369 and 2432 are specific ionic fragments of curcumin).

Infarct Volume. Mice were killed 72 h after MCAO (n = 10). The brains were harvested and 2,3,5-triphenyltetrazolium chloride (TTC) was used to stain the brain slices. In the survival tissue, TTC was a red formazan product, while the infarct areas were pale. The infarct area of each section (1 mm) was calculated by subtracting the non-infarcted area in the ipsilateral hemisphere from the total area of the contralateral hemisphere. Lastly, the total infarct volume was obtained by summing the infarct areas in all sections and multiplying by the section thickness.

Neurological Scores. The modified neurological severity scores (mNSS) test, consisting of a set of tests, was used to evaluate many aspects of neurological functions, including motor function

and sensory functional reflexes. (A translocator protein 18 kDa agonist protects against cerebral ischemia/reperfusion injury). The score was graded from 0 to 18. One point was given if a mouse failed to perform a test. A higher score indicates more severe impairment. The rating scale was as follows: a score of 13-18 indicates severe injury; 7-12 indicates moderate injury; 1-6 indicates mild injury. Two investigators blinded to the mouse groups performed the behavior tests to evaluate the neurological deficits at 1, 3, 7, 14 and 28 days after MCAO.

Open field test. The open-field test consisted of a square field (30 cm), with the floor divided into twelve parts. For a 5-min period, each animal was placed in the center of the arena, and we recorded the number of lines it crossed with its four paws. All tests were taped with a video recorder. After each test, the apparatus was carefully cleaned with 10% ethanol solution.

Elevated plus maze test. The elevated plus-maze plexiglass apparatus consisted of two open (30 × 5 cm) and two enclosed (30 × 5 × 25 cm) arms, with a central platform (5 × 5 cm). The open arms were surrounded by lateral bars (0.25 cm in height) to prevent the animal from falling. The apparatus was raised 38.5 cm above ground level. Each animal was placed on the central platform facing one of the enclosed arms. For 5 min, frequencies of entries into either open or enclosed arms and the time spent in each arm type were recorded (in seconds). The number of entries into the enclosed arms was used as an index of general activity. All tests were taped with a video recorder. After each test, the apparatus was carefully cleaned with 10% ethanol solution.

Immunohistochemistry. The mice were sacrificed and the brain tissues were excised at designed time points according to different *in vivo* experiments for subsequent immunofluorescence staining analysis. The harvested tissues were fixed using 4% paraformaldehyde solution at 4 °C for 24 h. After that, the fixed tissues were subjected to programmed dehydration using 5%, 15% and 30% sucrose solutions for three days, respectively (each sucrose solution for one day). Subsequently,

the samples were embedded in an optimum cutting temperature (OCT)-compound, sliced at a thickness of 20 μm with a freezing microtome (Leica CM1950) for further immunofluorescence staining by relevant antibodies as follows.

20 μm thickness mouse (n = 6 per group) brain slices were used. Immune staining was performed with anti-NeuN, anti-GFAP, anti-Iba1, and anti-CD31 antibodies. Nuclei were stained with 4′,6-diamidino-2-phenylindole dihydrochloride. Images were acquired using a confocal laser microscope (LSM 800 Zeiss, Germany)

For immunofluorescence staining analysis of infiltrating neutrophils in the injury region, the brain tissue slices from pre-designated time intervals post **BDP-4/Cur-CL** NPs treatment were washed three times in 1×PBS (each time for 5 min), which were then incubated with a commercial blocking buffer derived from goat serum for 30 min in a humidor at room temperature. Afterward, the blocking buffer was removed carefully from the slices and 50 μL of 1×PBS solution containing APC anti-mouse Ly-6G/Ly-6C (Gr-1) antibody (108412, BioLegend) (1:200) was added on the surface of each slice. After incubation in a humidor at 4 °C for 24 h, the brain slices were washed by 1×PBS three times (each time for 5 min) and further stained with DAPI to visualize cell nuclei. Finally, the fluorescence signals were collected with CLSM and analyzed with ImageJ software.

***In Vivo* Side Toxicity Evaluation**. The mice were sacrificed at days 3 and 28, and major organs and the brain were collected and fixed in 4% paraformaldehyde. Then they were embedded into paraffin and sliced at a thickness of 5 μm. Slices were stained with hematoxylin and eosin (H&E) and imaged by optical microscopy.

Statistical Analysis. All data are expressed as mean ± standard deviation (SD). GraphPad Prism 8 (GraphPad Software Inc., San Diego, CA, USA) was used for the statistical analysis. The Shapiro-Wilk test was used to verify whether the data conformed to a normal distribution. The

Brown-Forsythe test was used to test the homogeneity of the variance. Variables were compared between the groups using one- or two-way analyses of variance (ANOVA). $P < 0.05$ was considered statistically significant.

General information and methods

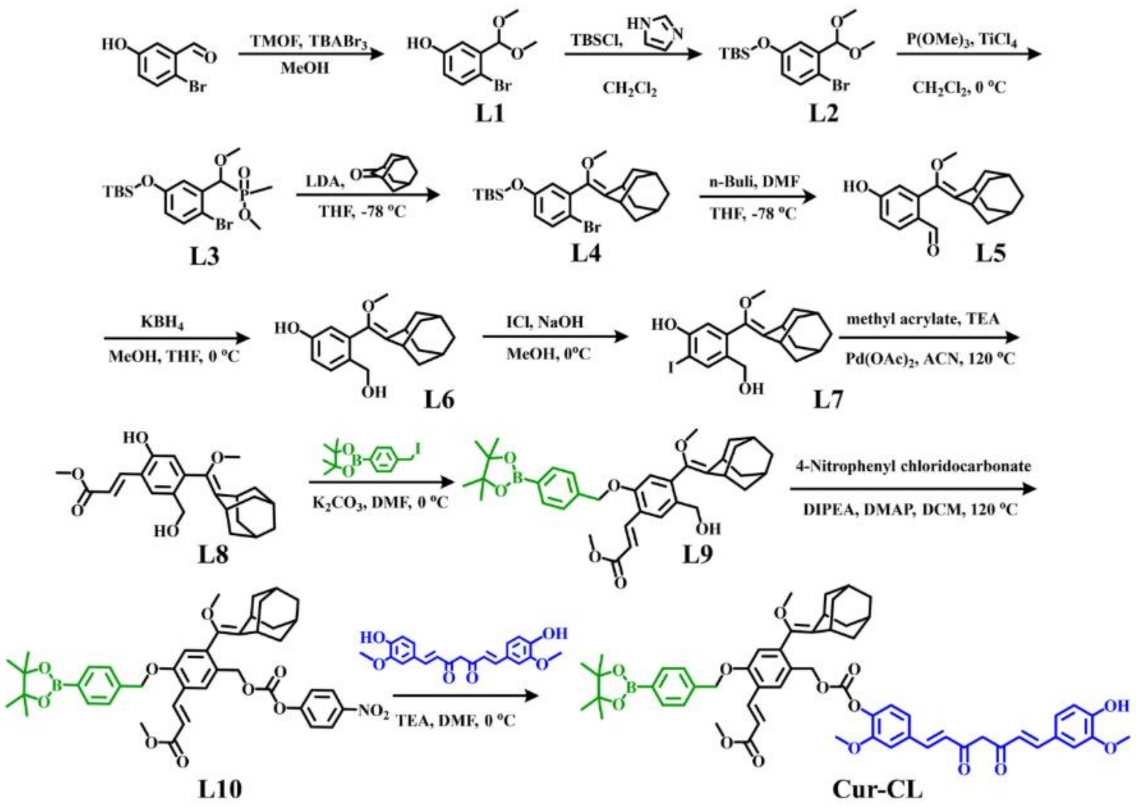

Scheme S1. Synthetic route to **Cur-CL**.

Synthesis of **L1**. A mixture of 2-bromo-5-hydroxybenzaldehyde (1.2 g, 6 mmol), trimethyl orthoformate (0.95 g, 9 mmol) and tetrabutylammonium tribromide (0.14 g, 0.3 mmol) in CH_3OH (100 mL) was stirred at ambient temperature for 24 h. The reaction was monitored by thin layer chromatography (TLC). After completion, it was extracted with EtOAc and washed with water. The EtOAc layer was separated and dried over Na_2SO_4. Then, the filtrate was removed by evaporation, the crude product was purified by column chromatography on silica gel using petroleum ether/ethyl acetate (v/v 20:1) as eluent to afford **L1** (1.32 g, 91.6 % yield). ^{1}H NMR

(400 MHz, $CDCl_3$, δ [ppm]): 7.36 (d, J = 8.6 Hz, 1H), 7.27 (d, J = 9.0 Hz, 1H), 7.13 (d, J = 3.1 Hz, 1H), 6.69 (dd, J = 8.6, 3.1 Hz, 1H), 5.53 (s, 1H,), 3.41 (s, 6H).

Synthesis of **L2**. A solution of compound **L1** (1.23 g, 5 mmol) and imidazole (0.68 g, 10 mmol) in DCM (60 mL) was stirred at ambient temperature for 5 min. Then, *tert*-butyl dimethyl silyl chloride (0.90 g, 6 mmol) was added and the mixture was stirred for 1 h. The reaction was monitored by TLC. After completion, it was extracted with DCM and washed with water. The DCM layer was separated and dried over Na_2SO_4. Subsequently, the filtrate was removed by evaporation, the crude product was purified by column chromatography on silica gel using petroleum ether/ethyl acetate (v/v 100:1) as eluent to afford **L2** (1.73 g, 96.1 % yield). ^{1}H NMR (400 MHz, $CDCl_3$, δ [ppm]): 7.38 (d, J = 6.5 Hz, 1H), 7.09 (d, J = 2.2 Hz, 1H), 6.69 (dd, J = 6.4, 2.3 Hz, 1H), 5.48 (s, 1H), 3.37 (s, 6H), 0.96 (s, 9H), 0.19 (s, 6H).

Synthesis of **L3**. Trimethylphosphite (0.60 mL, 5.85 mmol) was slowly added to a stirred solution of **L2** (1.62 g, 4.5 mmol) in DCM (30 mL) at 0 °C under argon. After the reaction mixture was stirred for 5 min, $TiCl_4$ (0.59 mL, 5.4 mmol) was injected and the mixture was stirred for another 1 h. The reaction was monitored by TLC. After completion, it was extracted with DCM and washed with water. The DCM layer was separated and dried over Na_2SO_4. Then the filtrate was removed by evaporation, the crude product was purified by column chromatography on silica gel using petroleum ether/ethyl acetate (v/v 50:1) as eluent to afford **L3** (1.62 g, yield 82.0%). ^{1}H NMR (400 MHz, $CDCl_3$, δ [ppm]): 7.38 (d, J = 8.7 Hz, 1H), 7.14 (t, J = 2.7 Hz, 1H), 6.70 (d, J = 8.7 Hz, 1H), 5.03 (d, J = 9.6 Hz, 1H), 3.77 (d, J = 9.6 Hz, 3H), 3.63 (d, J = 8.6 Hz, 3H), 3.33 (s, 3H), 0.95 (s, 9H), 0.19 (d, J = 4.2 Hz, 6H).

Synthesis of **L4**. Lithium diisopropylamide (1.8 mL, 2.0 M in THF/*n*-heptane/ethylbenzene, 3.6 mmol) was dropwise added to the solution of **L3** (1.32 g, 3 mmol) in dry THF (30 mL) at -78 °C

under argon. After the reaction mixture was stirred for 1 h, 2-adamantanone (0.56 g, 3.9 mmol) dissolved in dry THF (10 mL) was injected and the mixture was stirred for another 30 min. Then the mixture was stirred at ambient temperature for 1 h. The reaction was monitored by TLC. After completion, it was extracted with EtOAc and washed with water. The EtOAc layer was separated and dried over Na_2SO_4. Subsequently, the filtrate was removed by evaporation, the crude product was purified by column chromatography on silica gel using petroleum ether/ethyl acetate (v/v 200:1) as eluent to afford **L4** (1.12 g, yield 81.2%). ^{1}H NMR (400 MHz, $CDCl_3$, δ [ppm]): 7.43 (d, *J* = 8.6 Hz, 1H), 6.73 (d, *J* = 2.9 Hz, 1H), 6.69 (dd, *J* = 8.6, 2.9 Hz, 1H), 3.32 (s, 3H), 3.26 (s, 1H), 2.08 (s, 1H), 1.98 – 1.68 (m, 12H), 0.97 (s, 9H), 0.18 (s, 6H).

Synthesis of **L5**. To the solution of **L4** (1.11 g, 2.4 mmol) in dry THF (30 mL), *n*-BuLi (2 mL, 2.4 M in Hexane, 4.8 mmol) was added dropwise at -78 °C under argon. After the reaction mixture had been stirred for 1 h, dry DMF (1 mL) was injected and stirring was continued for another 30 min. Then the mixture was stirred at ambient temperature for 1 h. The reaction was monitored by TLC. After completion, it was extracted with EtOAc and washed with water. The EtOAc layer was separated and dried over Na_2SO_4. Subsequently, the filtrate was removed by evaporation, the crude product was purified by column chromatography on silica gel using petroleum ether/ethyl acetate (v/v 10:1) as eluent to afford **L5** (0.51 g, yield 71.3%). ^{1}H NMR (400 MHz, DMSO-d_6, δ [ppm]): 9.41 (s, 1H), 7.16 (t, J = 7.7 Hz, 1H), 6.77 – 6.58 (m, 3H), 3.20 (s, 3H), 3.16 (s, 1H), 2.57 (s, 1H), 1.96 – 1.61 (m, 12H).

Synthesis of **L6**. Potassium borohydride (0.14 g, 2.52 mmol) was added to a stirred solution of **L5** (0.5 g, 1.68 mmol) in THF:MeOH mixture (3:2 mL) at 0 °C for 20 min. The reaction was monitored by TLC. After completion, it was extracted with DCM and washed with water. The DCM layer was separated and dried over Na_2SO_4. Then, the filtrate was removed by evaporation

and the crude product was purified by column chromatography on silica gel using petroleum ether/ethyl acetate (v/v 2:1) as eluent to afford **L6** (0.46 g, yield 91.3%). ^{1}H NMR (400 MHz, $CDCl_3$, δ [ppm]): 7.40 (s, 1H), 7.22 (d, J = 8.2 Hz, 1H), 6.73 (dd, J = 8.1, 2.6 Hz, 1H), 6.69 (d, J = 2.6 Hz, 1H), 4.53 (d, J = 8.1 Hz, 2H), 3.28 (s, 3H), 3.24 (s, 1H), 2.26 (s, 1H), 1.98 – 1.64 (m, 12H).

Synthesis of **L7**. To a solution of **L6** (0.2 g, 0.67 mmol) in MeOH (20 mL), NaOH (27 mg, 0.67 mmol) was added slowly at 0 °C under argon. After the reaction mixture was stirred for 10 min, ICl (0.11 g, 0.68 mmol) dissolved in MeOH (10 mL) was injected and the mixture was stirred for another 30 min. The reaction was monitored by TLC. After completion, it was extracted with DCM and washed with water. The DCM layer was separated and dried over Na_2SO_4. Then the filtrate was removed by evaporation and the crude product was purified by column chromatography on silica gel using petroleum ether/ethyl acetate (v/v 1:1) as eluent to afford **L7** (0.23 g, yield 80.7%). ^{1}H NMR (400 MHz, $CDCl_3$, δ [ppm]): 7.74 (s, 1H), 6.81 (s, 1H), 5.47 (s, 1H), 4.48 (d, J = 9.6 Hz, 2H), 3.30 (s, 3H), 3.24 (s, 1H), 2.25 (s, 1H), 1.989 – 1.75 (m, 12H).

Synthesis of **L8**. A 35-mL thick-walled pressure bottle, was charged with **L7** (0.20 g, 0.469 mmol), methyl acrylate (0.12 g, 127 μL, 1.408 mmol), $Pd(OAc)_2$ (0.02g, 0.09 mmol), Et_3N (0.28 g, 390 μL, 2.81 mmol) and ACN (10 mL). The mixture was stirred at 120 °C under argon for 2 h. The reaction was monitored by TLC. After completion, it was extracted with EtOAc and washed with water. The EtOAc layer was separated and dried over Na_2SO_4. Then the filtrate was removed by evaporation and the crude product was purified by column chromatography on silica gel using petroleum ether/ethyl acetate (v/v 2:1) as eluent to afford **L8** (0.15 g, yield 83.3%). ^{1}H NMR (400 MHz, $CDCl_3$, δ [ppm]): 7.97 (d, J = 9.6 Hz, 1H), 7.52 (s, 1H), 6.69 (s, 1H), 6.60 (d, J = 9.6 Hz, 1H), 4.54 (s, 2H), 3.81 (s, 3H), 3.29 (s, 3H), 3.24 (s, 1H), 2.25 (s, 1H), 1.95 (d, J = 9.0 Hz, 4H), 1.85 – 1.74 (m, 8H).

Synthesis of **L9**. **L8** (0.1 g, 0.26 mmol) and K_2CO_3 (43 mg, 0.312 mmol) in DMF (5 mL) were stirred at 0 °C for 10 min. Then 4-(iodomethyl)benzeneboronic acid pinacol ester (0.98 g, 0.286 mmol) dissolved in DMF (1 mL) was added to the solution and the mixture was stirred for 10 min. Subsequently, the mixture was heated at ambient temperature for 1 h and monitored by TLC. After completion, it was extracted with EtOAc and washed with water. The EtOAc layer was separated and dried over Na_2SO_4. Then the filtrate was removed by evaporation and the crude product was purified by column chromatography on silica gel using petroleum ether/ethyl acetate (v/v 4:1) as eluent to afford **L9** (0.12 g, yield 76.9%). ^{1}H NMR (400 MHz, $CDCl_3$, δ [ppm]): 8.05 (d, *J* = 8.6 Hz, 1H), 7.80 (d, *J* = 8.1 Hz, 2H), 7.60 (s, 1H), 7.38 (d, *J* = 8.0 Hz, 2H), 6.68 (s, 1H), 6.58 (d, *J* = 9.6Hz, 1H), 5.20 (s, 2H), 4.55 (s, 2H), 3.80 (s, 3H), 3.23 (s, 4H), 2.07 (s, 1H), 1.99 – 1.70 (m, 12H), 1.34 (s, 12H).

Synthesis of **L10**. A mixture of the **L9** (0.12 g, 0.20 mmol) and 4-nitrophenyl chloroformate (0.06 g, 0.3 mmol) in dry DCM (20 mL) was stirred at 0 °C for 5 min. Subsequently, DIPEA (0.05 g, 70 μL, 0.4 mmol) and DMAP (1.2 mg, 0.01 mmol) were added into the above solution and the mixture was stirred at ambient temperature for 1 h and monitored by TLC. After completion, it was extracted with DCM and washed with water. The DCM layer was separated and dried over Na_2SO_4. Then the filtrate was removed by evaporation and the crude product was purified by column chromatography on silica gel using petroleum ether/ethyl acetate (v/v 6:1) as eluent to afford **L10** (0.11 g, yield 71.9%). ^{1}H NMR (400 MHz, $CDCl_3$, δ [ppm]): 8.28 (d, J = 9.2 Hz, 2H), 8.06 (d, J = 8.6 Hz, 1H), 7.82 (d, J = 8.1 Hz, 2H), 7.66 (s, 1H), 7.39 (t, J = 7.9 Hz, 4H), 6.77 (s, 1H), 6.61 (d, J = 9.6 Hz, 1H), 5.30 (s, 2H), 5.23 (s, 2H), 3.84 (s, 3H), 3.25 (d, J = 8.6 Hz, 4H), 2.11 (s, 1H), 1.96 – 1.69 (m, 12H), 1.35 (d, J = 3.4 Hz, 12H).

Synthesis of **Cur-CL**. **L10** (0.15 g, 0.20 mmol) and curcumin (0.37 g, 1 mmol) in dry DMF (20 mL) was stirred at 0 °C for 10 min. Triethylamine (0.04 g, 55 μL, 0.4 mmol) was added into the above solution and the mixture was stirred at ambient temperature for 24 h. After completion, it was extracted with DCM and washed with water. The DCM layer was separated and dried over Na_2SO_4. Subsequently, the filtrate was removed by evaporation, the crude product was purified by column chromatography on silica gel using petroleum ether/ethyl acetate (v/v 5:1) as eluent to afford **Cur-CL** (0.125 g, yield 62.9%). ^{1}H NMR (400 MHz, $CDCl_3$, δ [ppm]): 15.94 (s, 1H), 8.15 (d, J = 9.0 Hz, 1H), 8.06 (d, J = 8.6 Hz, 1H), 7.82 (d, J = 8.0 Hz, 2H), 7.67 (s, 1H), 7.60 (dd, J = 8.6, 6.6 Hz, 2H), 7.40 (d, J = 8.0 Hz, 2H), 7.13 (d, J = 9.6 Hz, 4H), 7.05 (s, 1H), 6.95 – 6.87 (m, 2H), 6.75 (s, 1H), 6.67 – 6.31 (m, 4H), 5.84 (s, 1H), 5.24 (d, J = 9.6 Hz, 5H), 3.86 (s, 3H), 3.82 (s, 3H), 3.24 (s, 1H), 3.21 (s, 3H), 2.09 (s, 1H), 1.92 (d, J = 8.6 Hz, 4H), 1.83 – 1.64 (m, 8H), 1.35 (s, 12H). ^{13}C NMR (151 MHz, $CDCl_3$, δ [ppm]): 184.61, 181.68, 167.81, 156.49, 152.94, 151.45, 148.01, 146.80, 141.45, 141.19, 140.51, 139.41, 139.28, 139.23, 137.84, 135.15, 134.27, 132.92, 129.48, 127.54, 127.11, 126.28, 126.18, 124.39, 123.50, 123.07, 122.80, 121.77, 120.88, 119.01, 115.62, 115.35, 114.85, 111.55, 109.64, 101.55, 83.86, 70.42, 67.80, 60.44, 57.19, 55.96, 51.71, 37.01, 32.53, 29.71, 28.19, 24.83, 21.05, 14.18. ESI-MS: m/z 993.64 $[M-H]^-$ (calcd: 994.43), Anal. Calcd. for $C_{58}H_{63}BO_{14}$: C 70.02, H; 6.38; Found C 70.01, H 6.36.

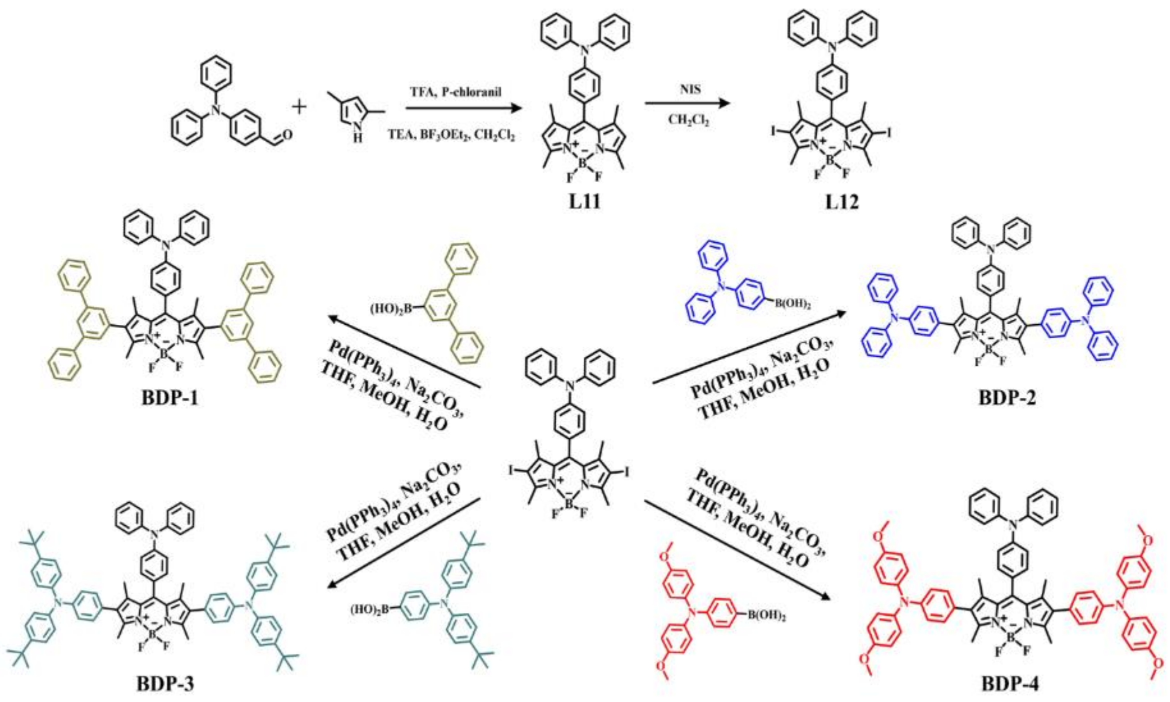

Scheme S2. Synthetic routes to **BDP-1**, **BDP-3** and **BDP-4**.

Synthesis of **L11**. A mixture of 4-(diphenylamino)benzaldehyde (0.54 g, 2 mmol), 1,4-dimethylpyrrole (0.39 g, 4.2 mmol) and two drops of trifluoroacetic acid in dry DCM (150 mL) was stirred at ambient temperature under argon in the dark for 24 h. Para-chloranil (0.49 g, 2 mmol) was injected and the mixture was stirred for another 1 h. Subsequently, triethylamine (10 mL) and boron trifluoride ether (10 mL) were added dropwise into the solution at 0 °C during 15 min. Then, the mixture was stirred at ambient temperature under argon for 4 h. After completion, it was extracted with DCM and washed with water. The DCM layer was separated and dried over Na_2SO_4. Subsequently, the filtrate was removed by evaporation and the crude product was purified by column chromatography on silica gel using petroleum ether/ethyl acetate (v/v 2:1) as eluent to afford **L11** (0.499 g, yield 50.9%). ^{1}H NMR (400 MHz, $CDCl_3$, δ [ppm]): 7.29 (t, J = 7.8 Hz, 4H), 7.17 (d, J = 8.5 Hz, 2H), 7.15 – 6.99 (m, 8H), 6.01 (s, 2H), 2.56 (s, 6H), 1.59 (s, 6H).

Synthesis of **L12**. A mixture of **L11** (0.49 g, 1 mmol) and *N*-iodosuccinimide (NIS) (0.9 g, 2.5 mmol) in dry DCM (100 mL) was stirred at ambient temperature for 30 min. After completion, it was extracted with DCM and washed with water. The DCM layer was separated and dried over Na_2SO_4. Subsequently, the filtrate was removed by evaporation and the crude product was purified by column chromatography on silica gel using petroleum ether/ethyl acetate (v/v 100:1) as eluent to afford **L12** (0.63 g, yield 84.8%). ^{1}H NMR (400 MHz, $CDCl_3$, δ [ppm]): 7.25 – 7.18 (m, 4H), 7.11 (d, J = 8.3 Hz, 2H), 7.08 – 6.89 (m, 8H), 2.57 (s, 6H), 1.55 (s, 6H).

Synthesis of **BDP-1**. **L12** (0.37 g, 0.5 mmol), (5'-phenyl-[1,1':3',1''-terphenyl]-4-yl)boronic acid (0.42 g, 1.2 mmol), $Pd(PPh_3)_4$ (0.058 g, 0.05 mmol) and sodium carbonate solution (2 M, 10 mL) were dissolved in THF (40 mL) and CH_3OH (10 mL). The mixture was stirred at 80 °C for 24 h under a nitrogen atmosphere. After cooling the reaction mixture to ambient temperature, it was extracted with DCM and washed with water. The DCM layer was separated and dried over Na_2SO_4. After solvent evaporation, the crude product was purified by column chromatography on silica gel using petroleum ether/ethyl acetate (v/v 3:1) as eluent to afford **BDP-1** (0.22 g, yield 46.5%). ^{1}H NMR (400 MHz, $CDCl_3$, δ [ppm]): 7.78 (s), 7.67 (d, J = 7.4 Hz, 2H), 7.48 (t, J = 7.5 Hz, 9H), 7.43 – 7.36 (m, 9H), 7.25 – 7.17 (m, 8H), 7.10 – 6.97 (m, 6H), 2.65 (s, 6H), 1.64 (s, 6H). ^{13}C NMR (151 MHz, $CDCl_3$, δ [ppm]): 154.19, 148.76, 147.33, 141.90, 140.91, 139.33, 134.78, 133.61, 131.77, 129.48, 128.99, 128.90, 127.92, 127.60, 127.30, 124.91, 124.71, 123.58, 123.48, 13.60, 13.28. MS: (MALDI-TOF) [m/z]: 947.4 (calcd: 947.42). Anal. Calcd. for $C_{67}H_{52}BF_2N_3$: C 84.89, H 5.53, N 4.43; Found C 84.87, H 5.56, N 4.42.

Synthesis of **BDP-3**. **L12** (0.37 g, 0.5 mmol), (4-(bis(4-(tert-butyl)phenyl)amino)phenyl)boronic acid (0.48 g, 1.2 mmol), $Pd(PPh_3)_4$ (0.058g, 0.05 mmol) and sodium carbonate solution (2 M, 10 mL) was dissolved in THF (40 mL) and CH_3OH (10 mL). The mixture was stirred at 80 °C for 24

h under a nitrogen atmosphere. After cooling the reaction mixture to ambient temperature, it was extracted with DCM and washed with water. The DCM layer was separated and dried over Na_2SO_4. After solvent evaporation, the crude product was purified by column chromatography on silica gel using petroleum ether/ethyl acetate (v/v 3:1) as eluent to afford **BDP-3** (0.24 g, yield 39.9%). ^{1}H NMR (400 MHz, $CDCl_3$, δ [ppm]): 7.28 (d, J = 8.1 Hz, 11H), 7.20 (d, J = 1.9 Hz, 4H), 7.11 – 7.00 (m, 24H), 2.58 (s, 6H), 1.57 (s, 6H), 1.33 (s, 36H).^{13}C NMR (151 MHz, $CDCl_3$, δ [ppm]): 154.11, 148.58, 147.38, 147.01, 145.76, 145.52, 145.03, 144.93, 141.71, 138.72, 131.54, 130.71, 129.41, 129.06, 128.98, 127.02, 126.58, 126.04, 125.98, 124.53, 124.12, 123.85, 123.72, 123.32, 122.22, 60.37, 34.28, 31.43, 21.03, 14.19, 13.49, 13.14, 1.01. MS: (MALDI-TOF) [m/z]: 1201.7 (calcd: 1201.69). Anal. Calcd. for $C_{83}H_{86}BF_2N_5$: C 82.91, H 7.21, N 5.82. Found C 82.91, H 7.20, N 5.84.

Synthesis of **BDP-4**. **L12** (0.37 g, 0.5 mmol), (4-(bis(4-methoxyphenyl)amino)phenyl)boronic acid (0.42 g, 1.2 mmol), $Pd(PPh_3)_4$ (0.058g, 0.05 mmol) and sodium carbonate solution (2 M, 10 mL) was dissolved in THF (40 mL) and CH_3OH (10 mL). The mixture was stirred at 80 °C for 24 h under a nitrogen atmosphere. After cooling down the reaction mixture to ambient temperature, it was extracted with DCM and washed with water. The DCM layer was separated and dried over Na_2SO_4. After solvent evaporation, the crude product was purified by column chromatography on silica gel using petroleum ether/ethyl acetate (v/v 3:1) as eluent to afford **BDP-4** (0.25 g, yield 45.5%). ^{1}H NMR (400 MHz, $CDCl_3$, δ [ppm]): 7.25 – 7.20 (m, 4H), 7.16 (s, 4H), 7.11 – 6.98 (m, 14H), 6.93 (q, J = 8.7 Hz, 8H), 6.82 (d, J = 8.9 Hz, 8H), 3.78 (s, 12H), 2.53 (s, 6H), 1.52 (s, 6H). ^{13}C NMR (151 MHz, $CDCl_3$, δ [ppm]): 155.45, 148.16, 147.16, 146.93, 140.38, 130.18, 128.94, 128.60, 126.26, 124.85, 124.06, 123.22, 122.85, 119.33, 114.25, 55.02, 12.64. MS: (MALDI-TOF) [m/z]: 1097.5 (calcd: 1097.49). Anal. Calcd. for $C_{71}H_{62}BF_2N_5O_4$: C 77.66, H 5.69, N6.38; Found C 77.67, H 5.66, N6.41.

Note: Some of the ^{1}H NMR spectra contain peaks from chromatography solvent and silicone vacuum grease that could not be removed in purification.

Supplementary Figures

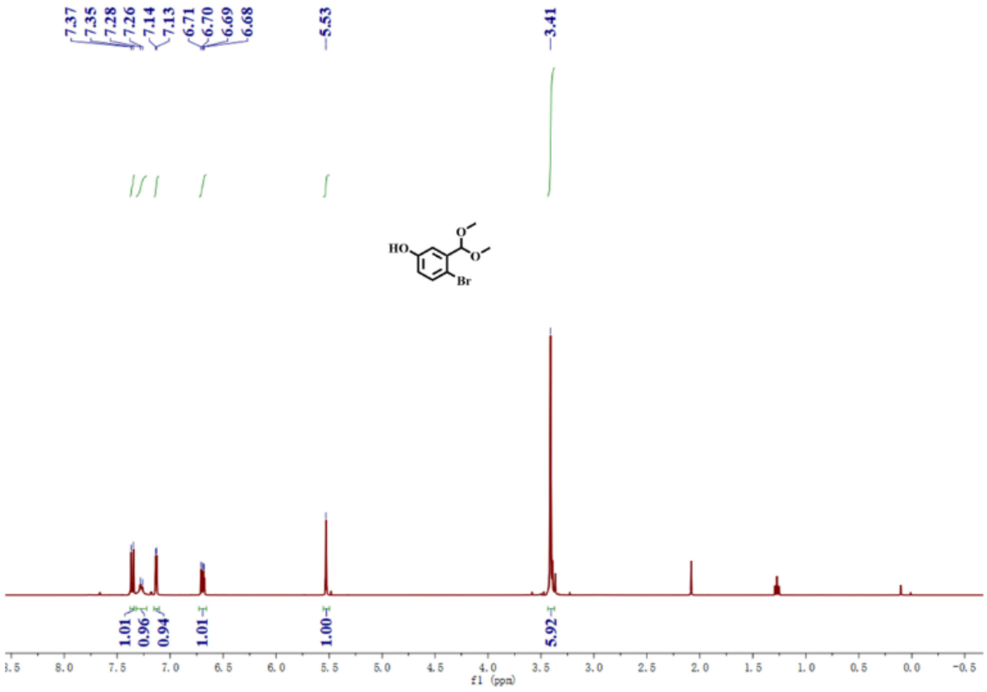

Figure S1. ^{1}H NMR spectrum of **L1** in $CDCl_3$.

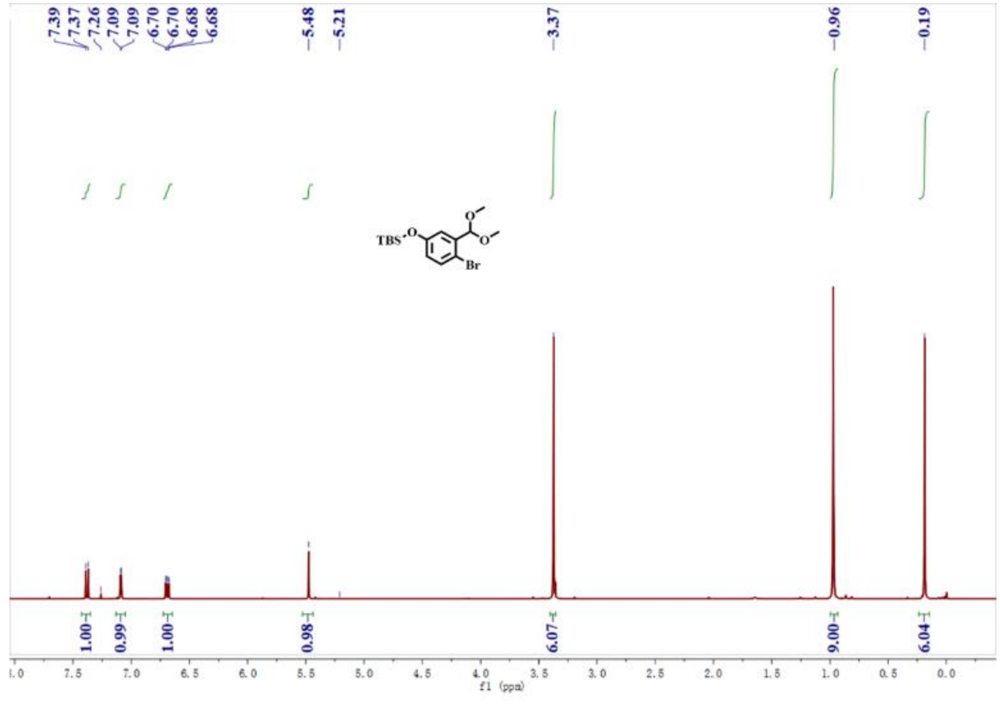

Figure S2. ^{1}H NMR spectrum of **L2** in $CDCl_3$.

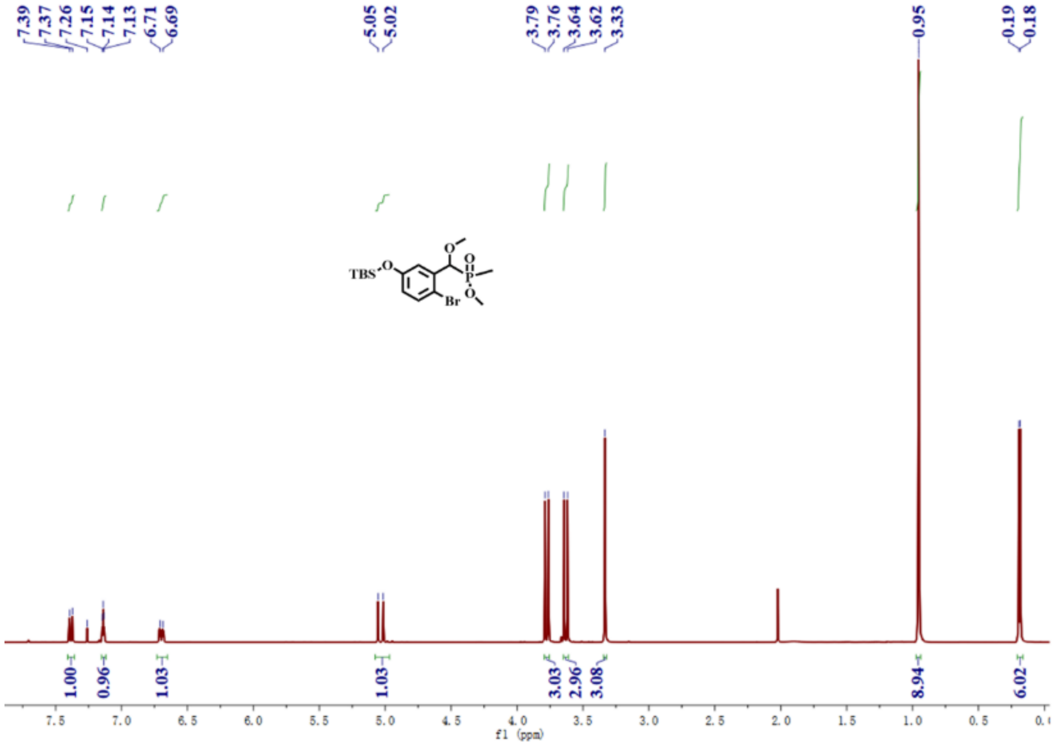

Figure S3. ^{1}H NMR spectrum of **L3** in $CDCl_3$.

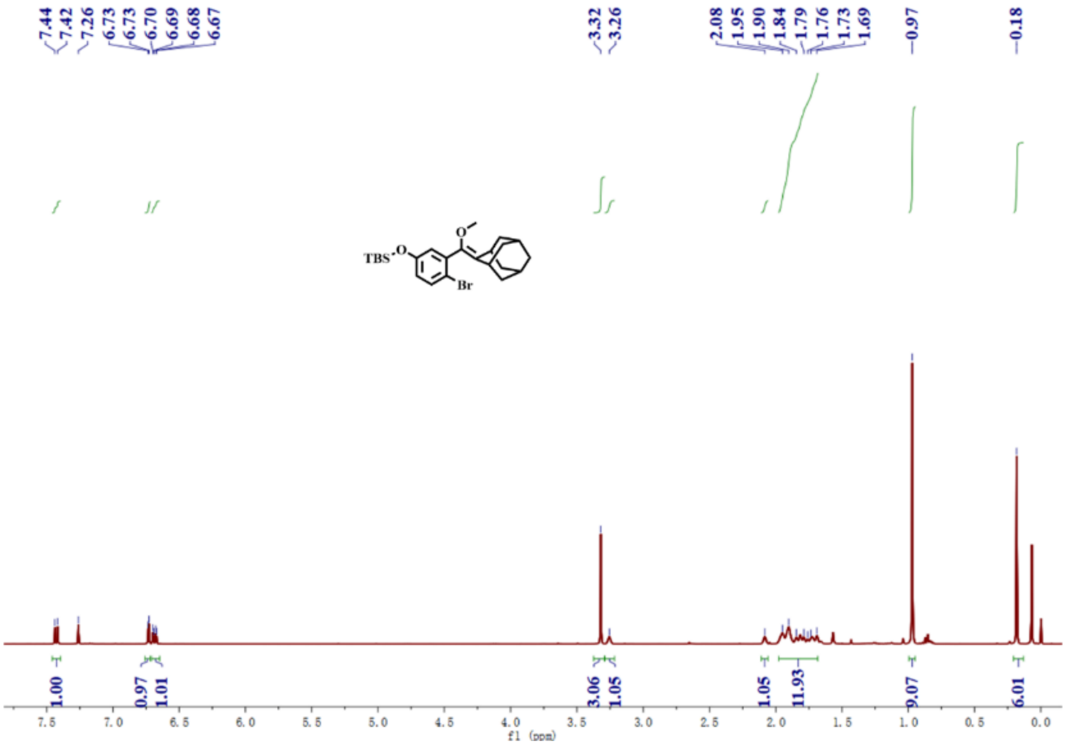

Figure S4. ^{1}H NMR spectrum of **L4** in $CDCl_3$.

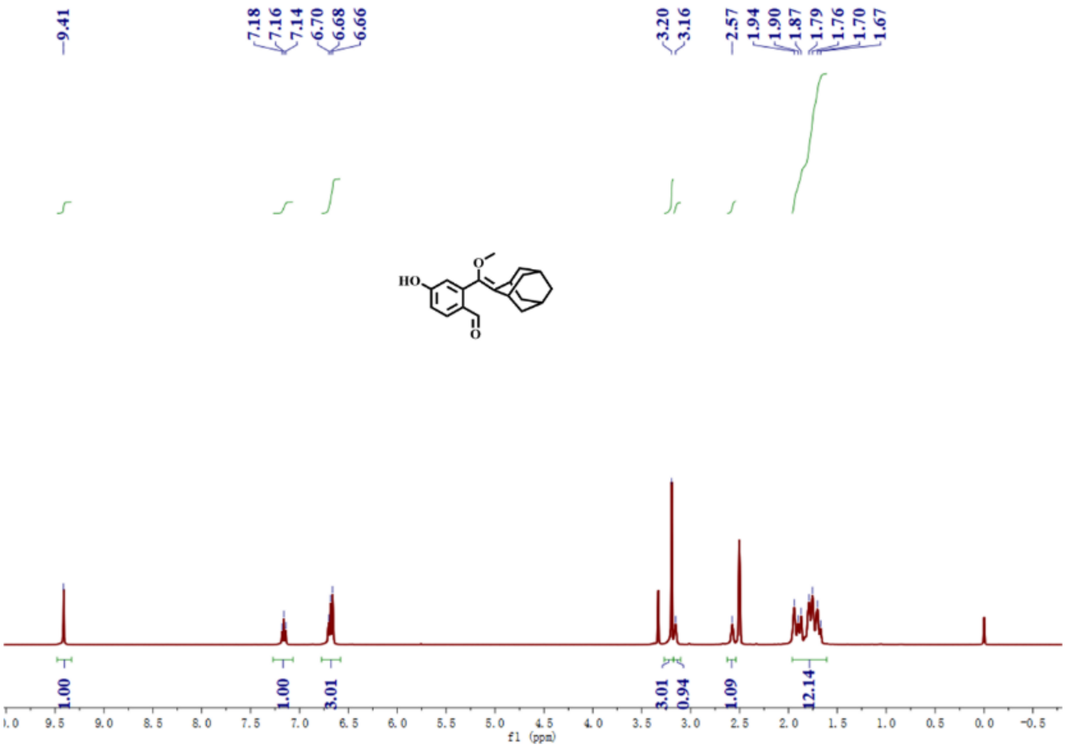

Figure S5. ^{1}H NMR spectrum of **L5** in DMSO-d_6.

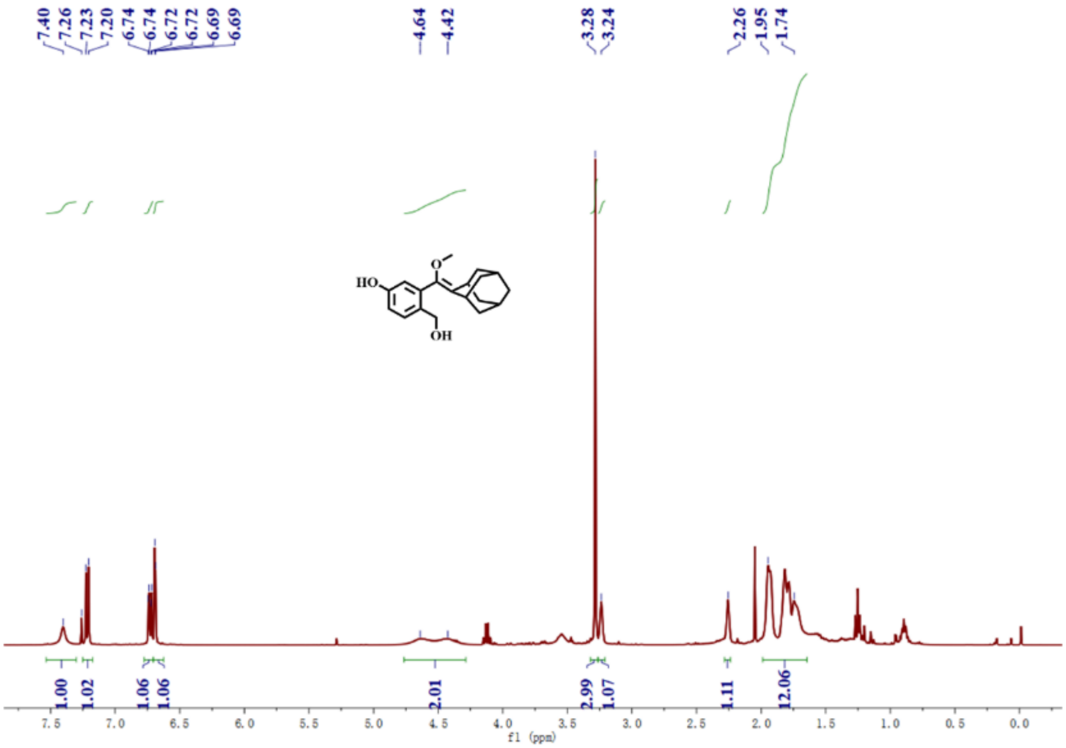

Figure S6. ^{1}H NMR spectrum of **L6** in $CDCl_3$.

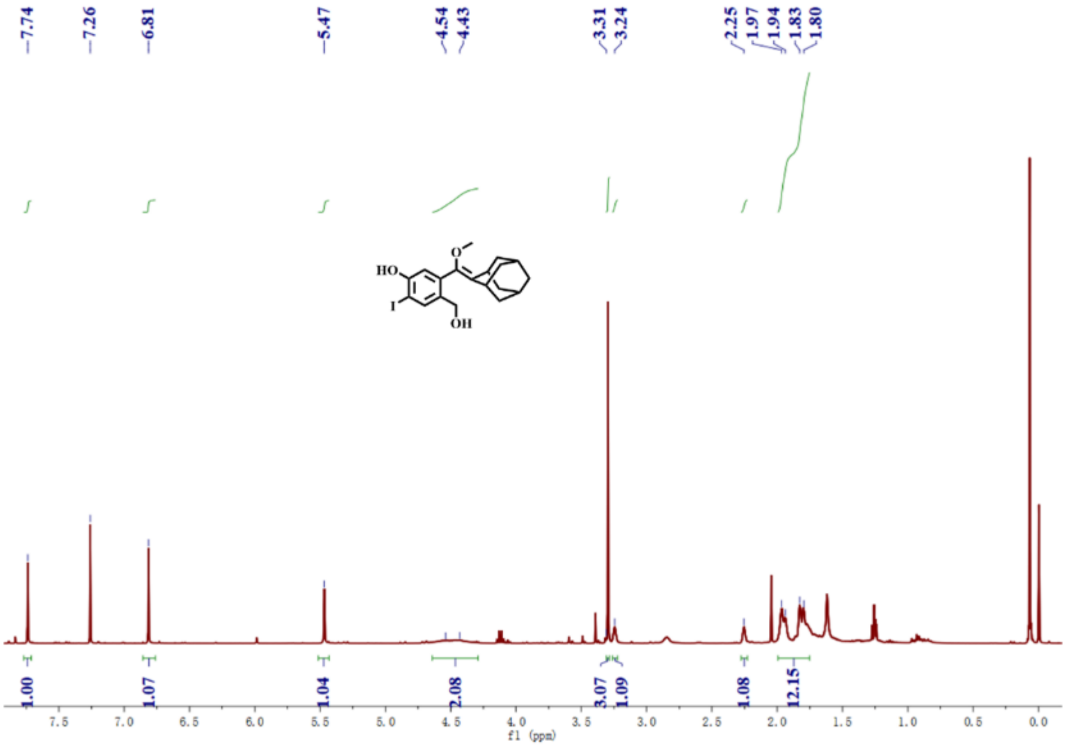

Figure S7. ^{1}H NMR spectrum of **L7** in $CDCl_3$.

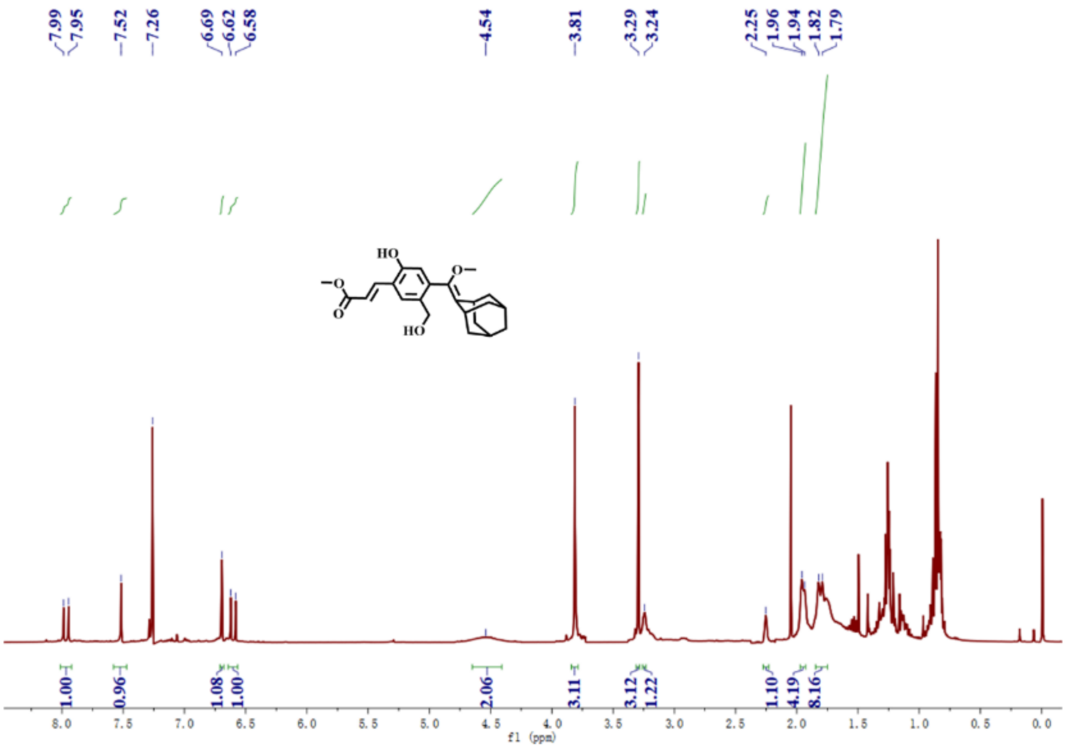

Figure S8. ^{1}H NMR spectrum of **L8** in $CDCl_3$.

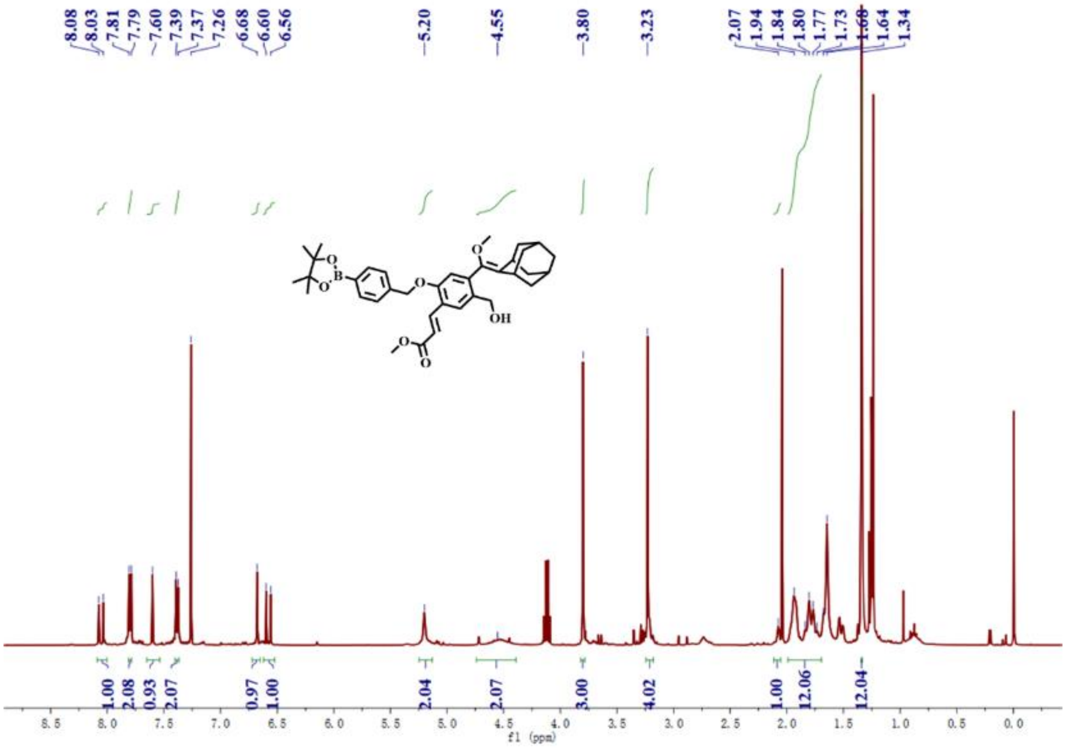

Figure S9. ^{1}H NMR spectrum of **L9** in $CDCl_3$.

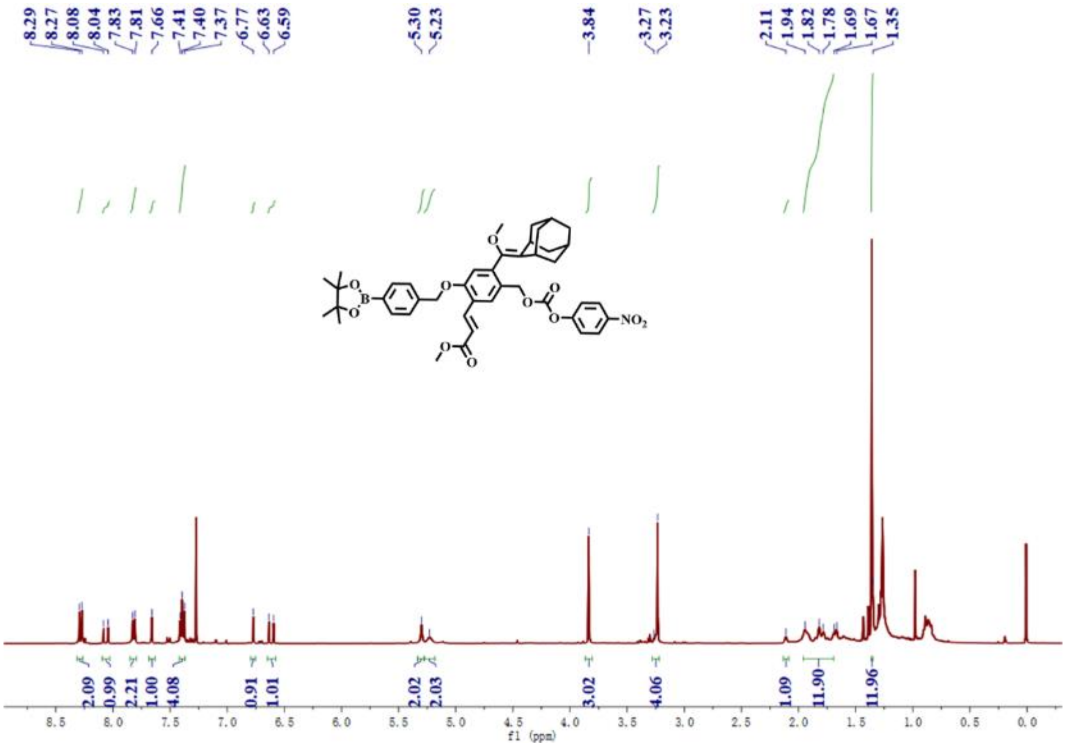

Figure S10. ^{1}H NMR spectrum of **L10** in $CDCl_3$.

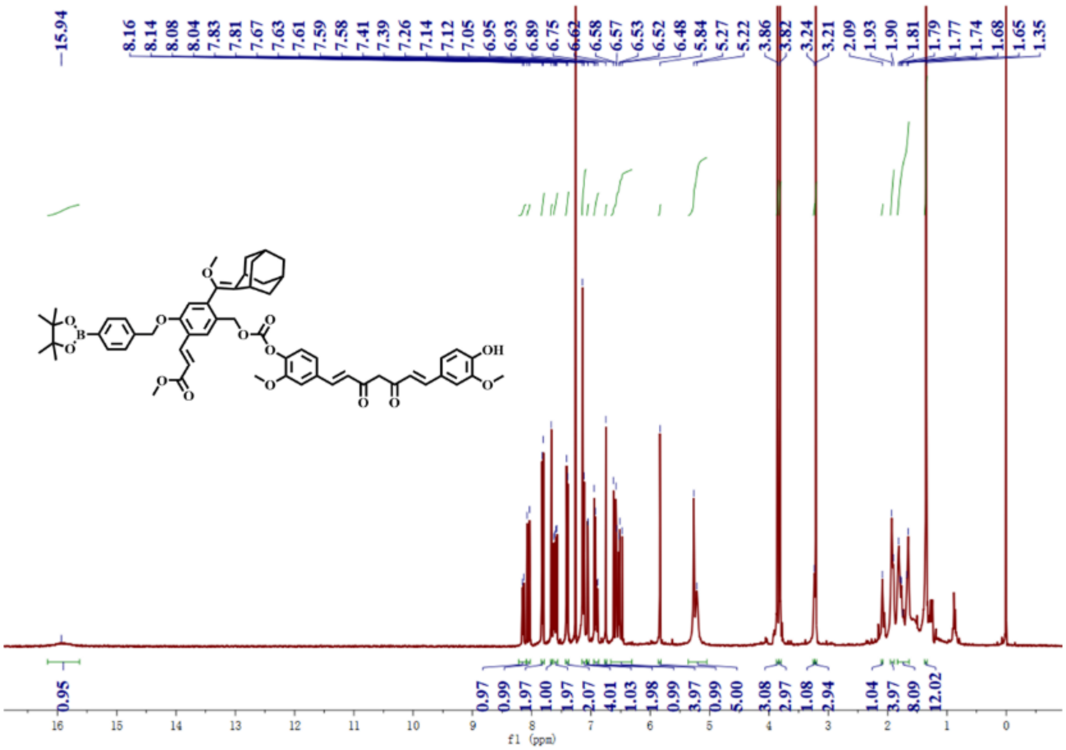

Figure S11. ^{1}H NMR spectrum of **Cur-CL** in $CDCl_3$.

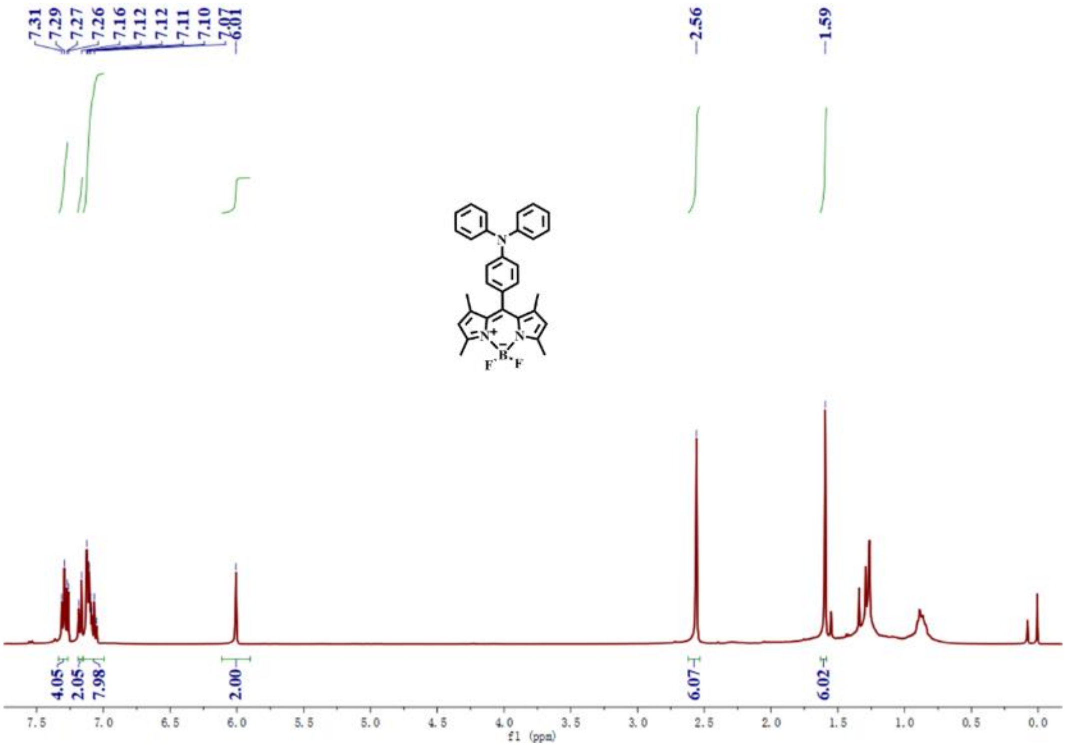

Figure S12. ^{1}H NMR spectrum of **L11** in $CDCl_3$.

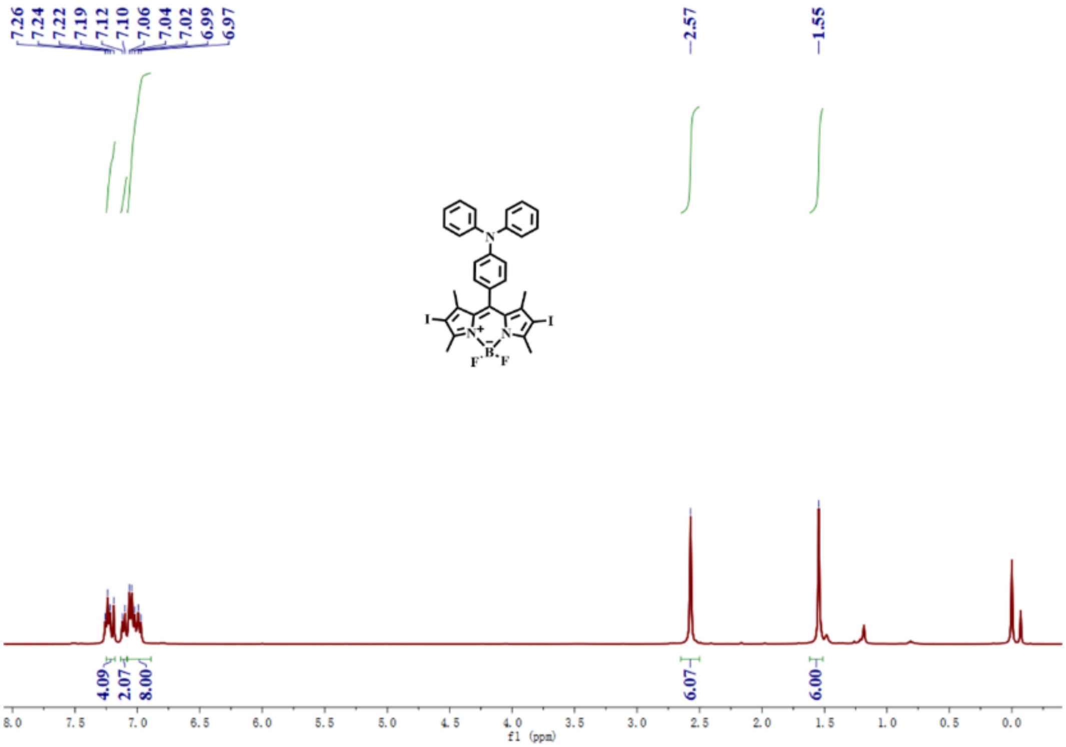

Figure S13. ^{1}H NMR spectrum of **L12** in $CDCl_3$.

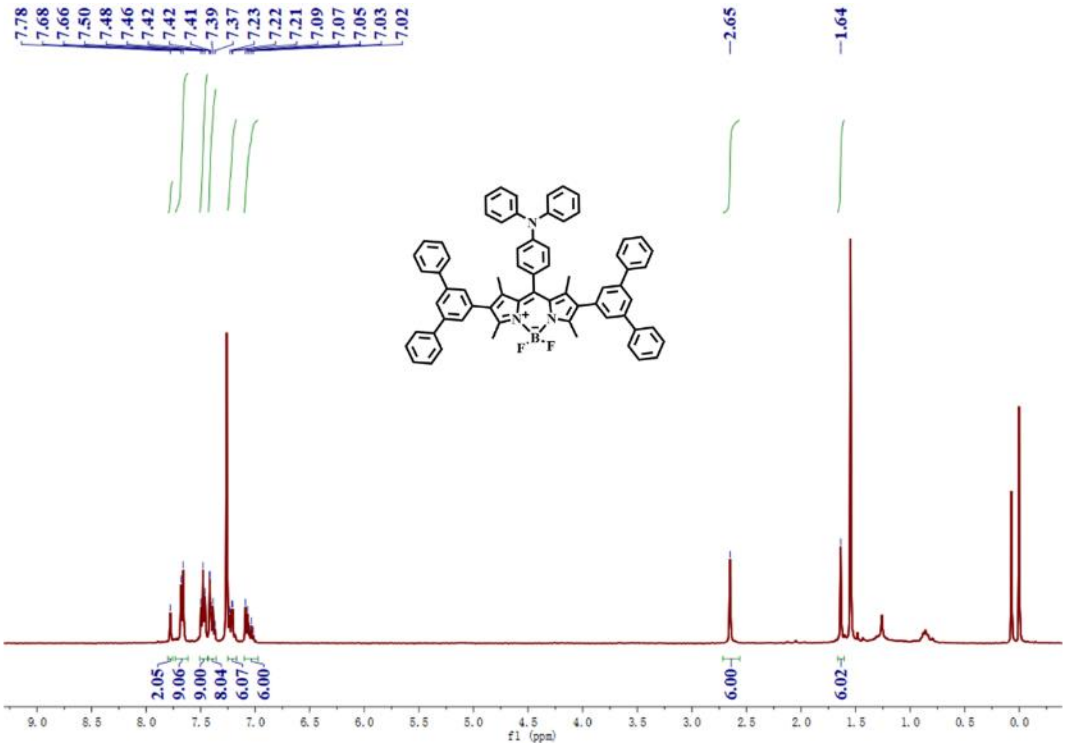

Figure S14. ^1H NMR spectrum of **BDP-1** in $CDCl_3$.

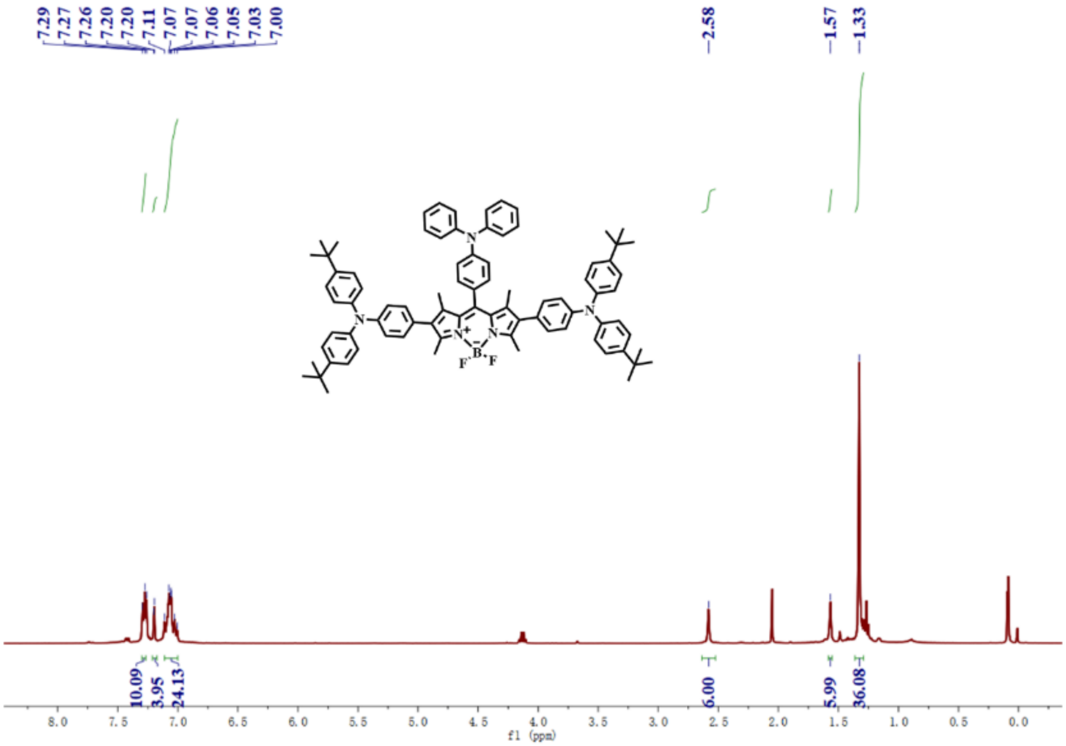

Figure S15. ^1H NMR spectrum of **BDP-3** in $CDCl_3$.

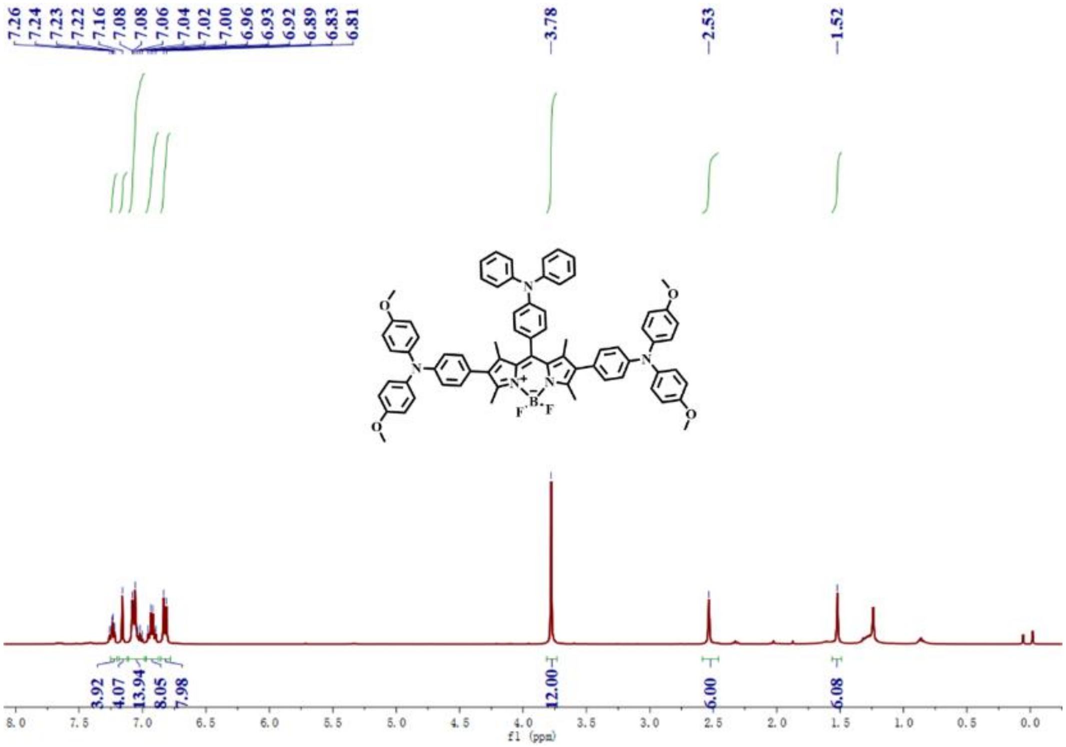

Figure S16. ^{1}H NMR spectrum of **BDP-4** in $CDCl_3$.

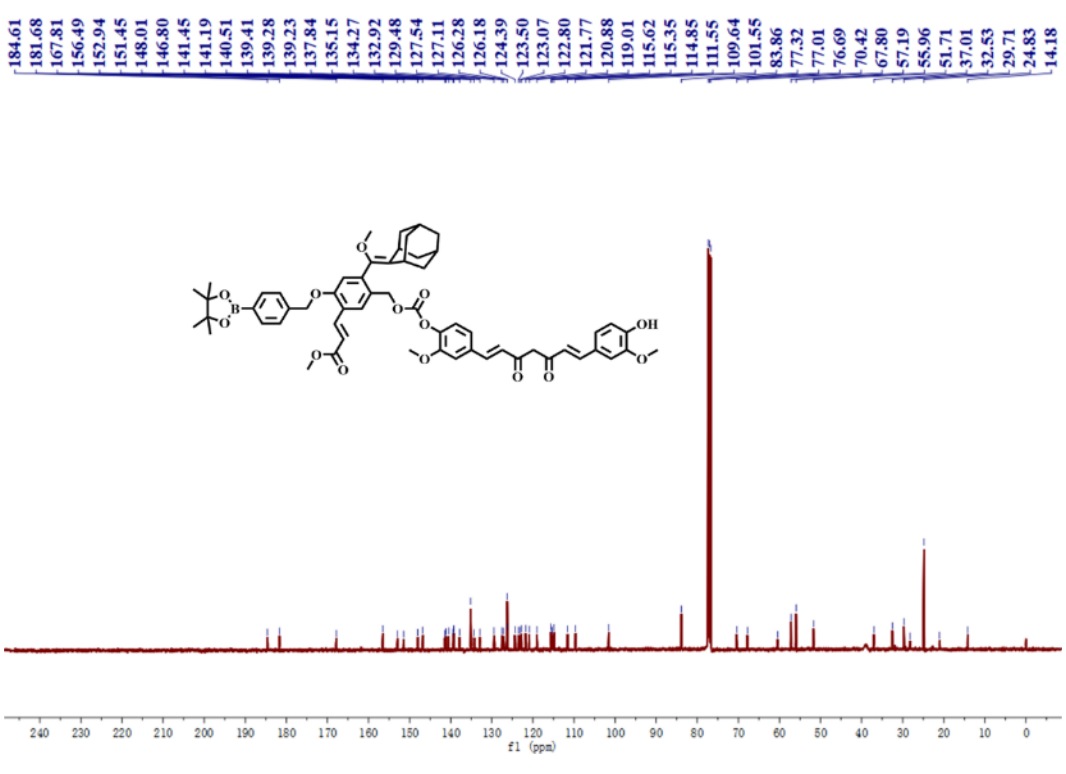

Figure S17. ^{13}C NMR spectrum of **Cur-CL** in $CDCl_3$.

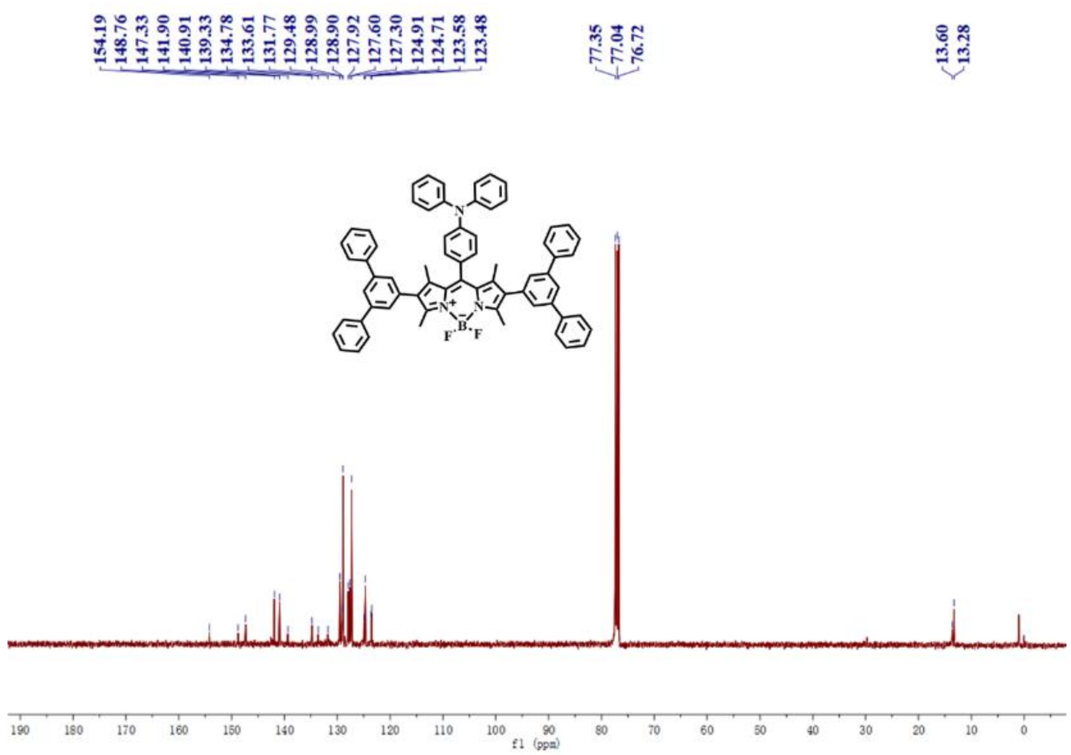

Figure S18. ^{13}C NMR spectrum of **BDP-1** in $CDCl_3$.

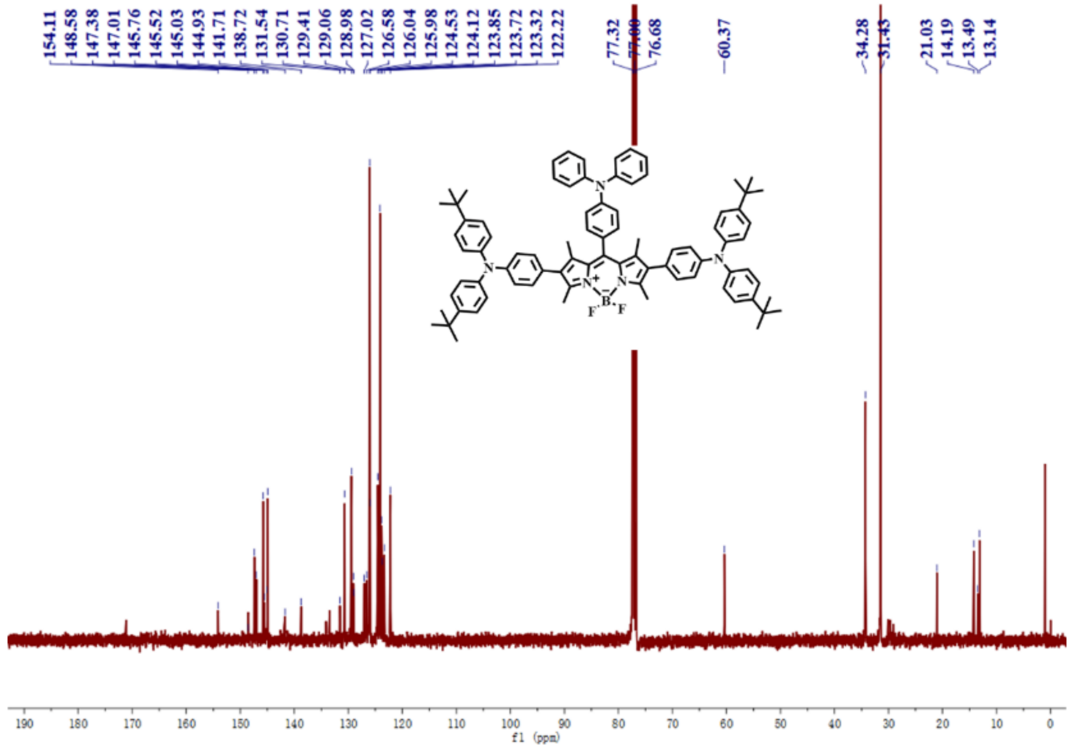

Figure S19. ^{13}C NMR spectrum of **BDP-3** in $CDCl_3$.

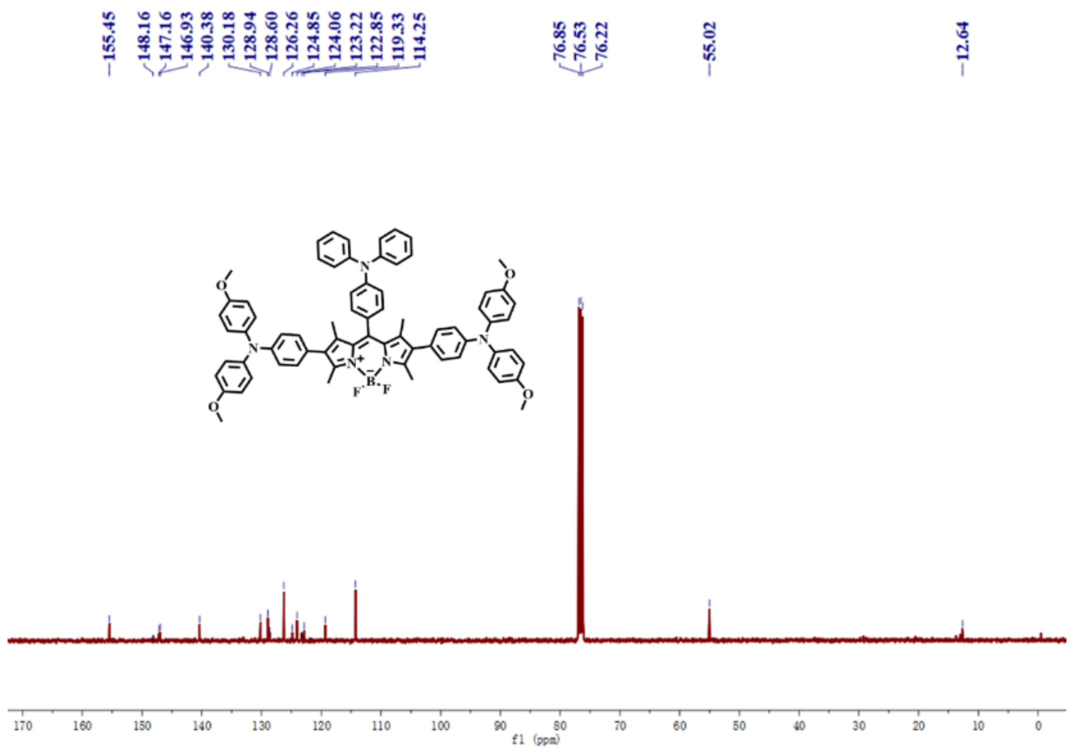

Figure S20. ^{13}C NMR spectrum of **BDP-4** in $CDCl_3$.

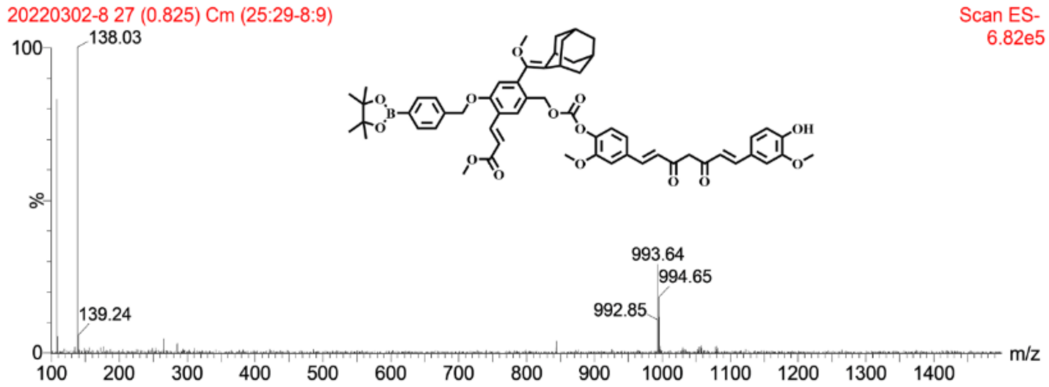

Figure S21. ESI mass spectrum of **Cur-CL**.

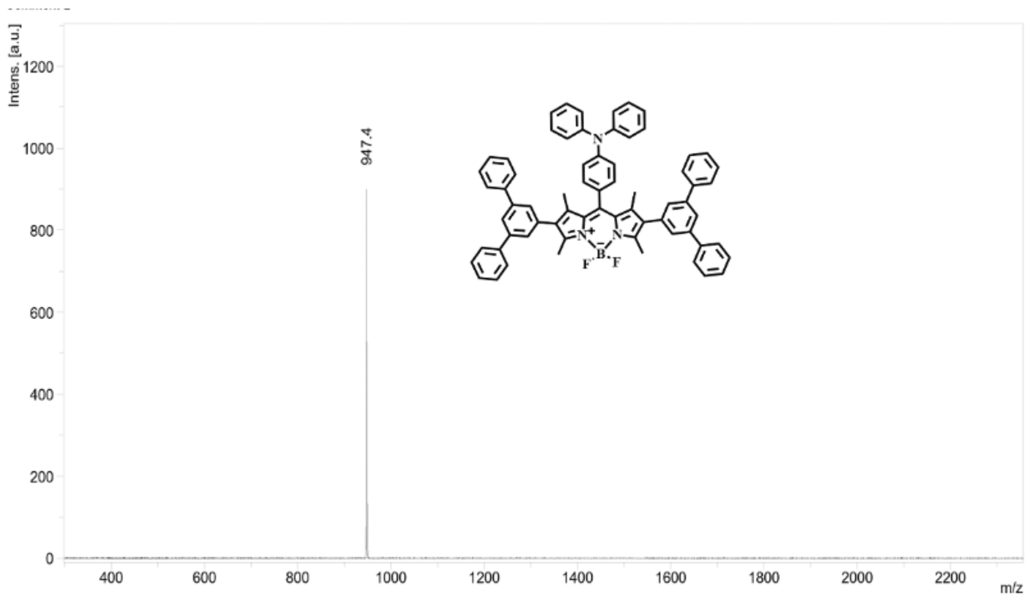

Figure S22. MALDI-TOF mass spectrum of **BDP-1**.

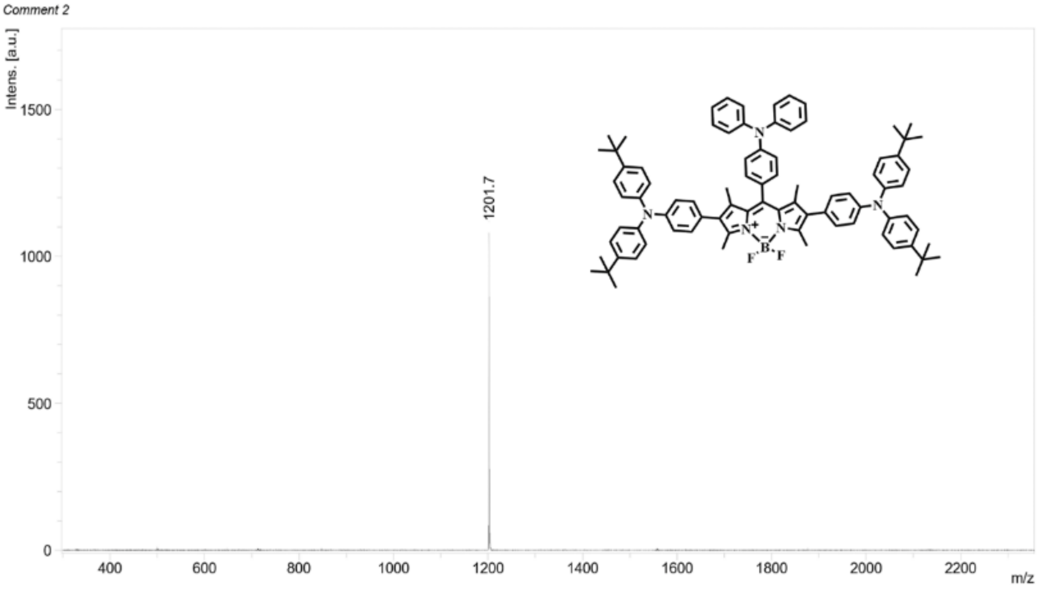

Figure S23. MALDI-TOF mass spectrum of **BDP-3**.

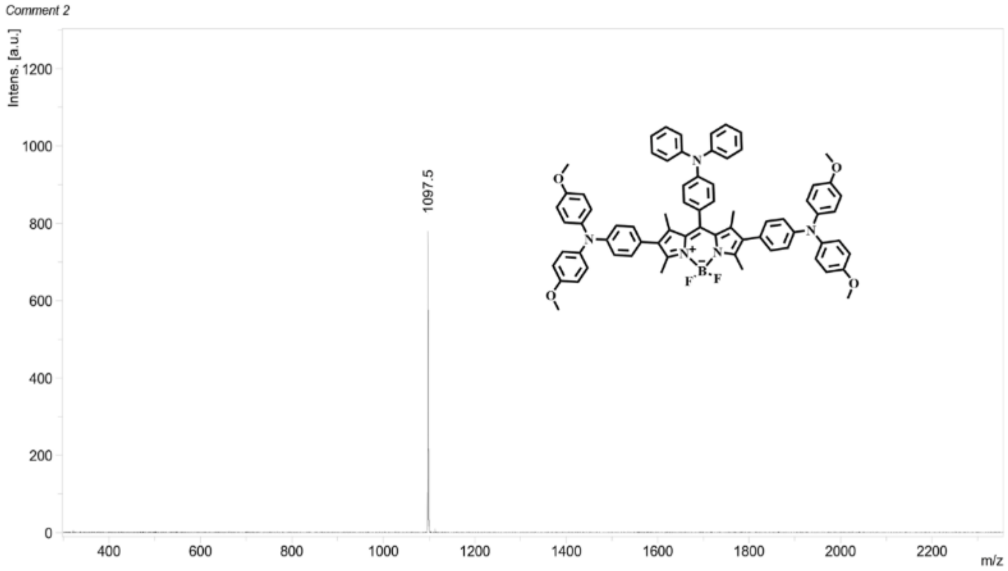

Figure S24. MALDI-TOF mass spectrum of **BDP-4**.

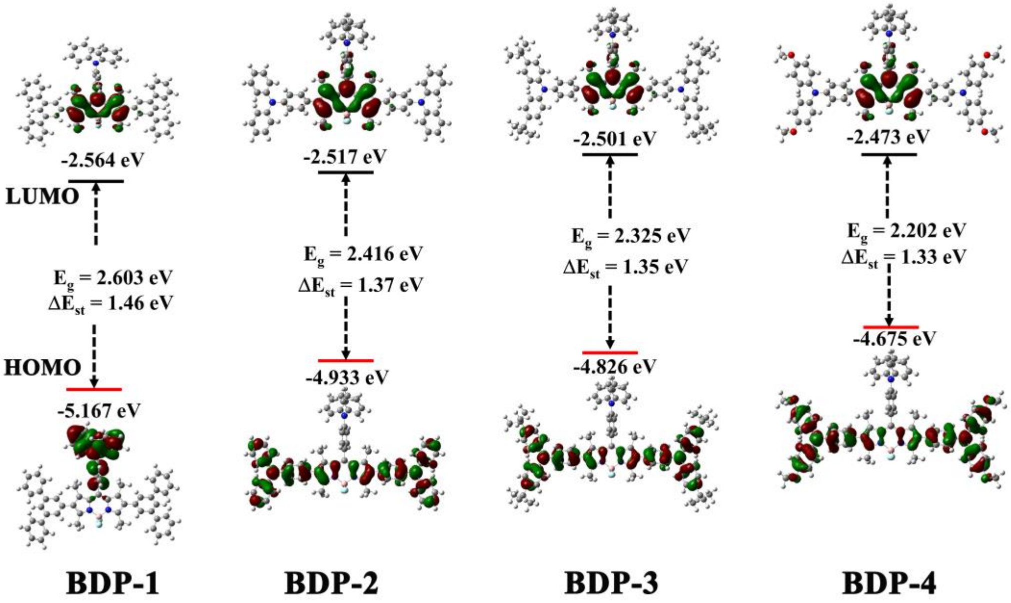

Figure S25. Optimized molecular geometry, HOMO–LUMO distributions and ΔE_{ST} values for **BDP-1**, **BDP-2**, **BDP-3** and **BDP-4** as calculated by DFT.

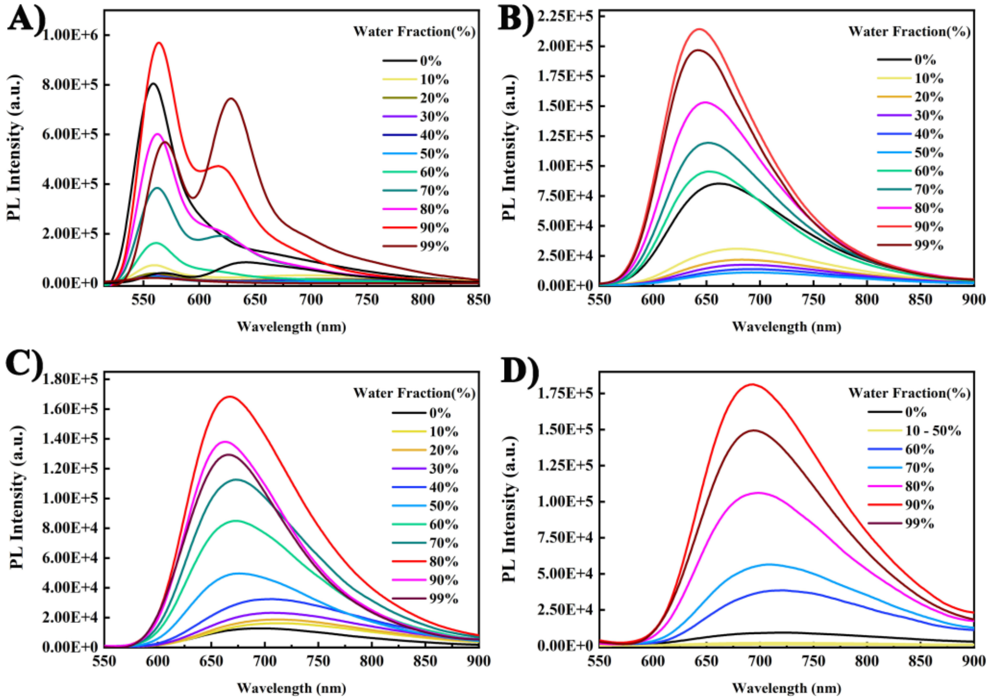

Figure S26. Emission spectra of A) **BDP-1**, B) **BDP-2**, C) **BDP-3** and D) **BDP-4** (10^{-5} M) in THF-water mixtures with different water fractions (0-99% v/v) at room temperature.

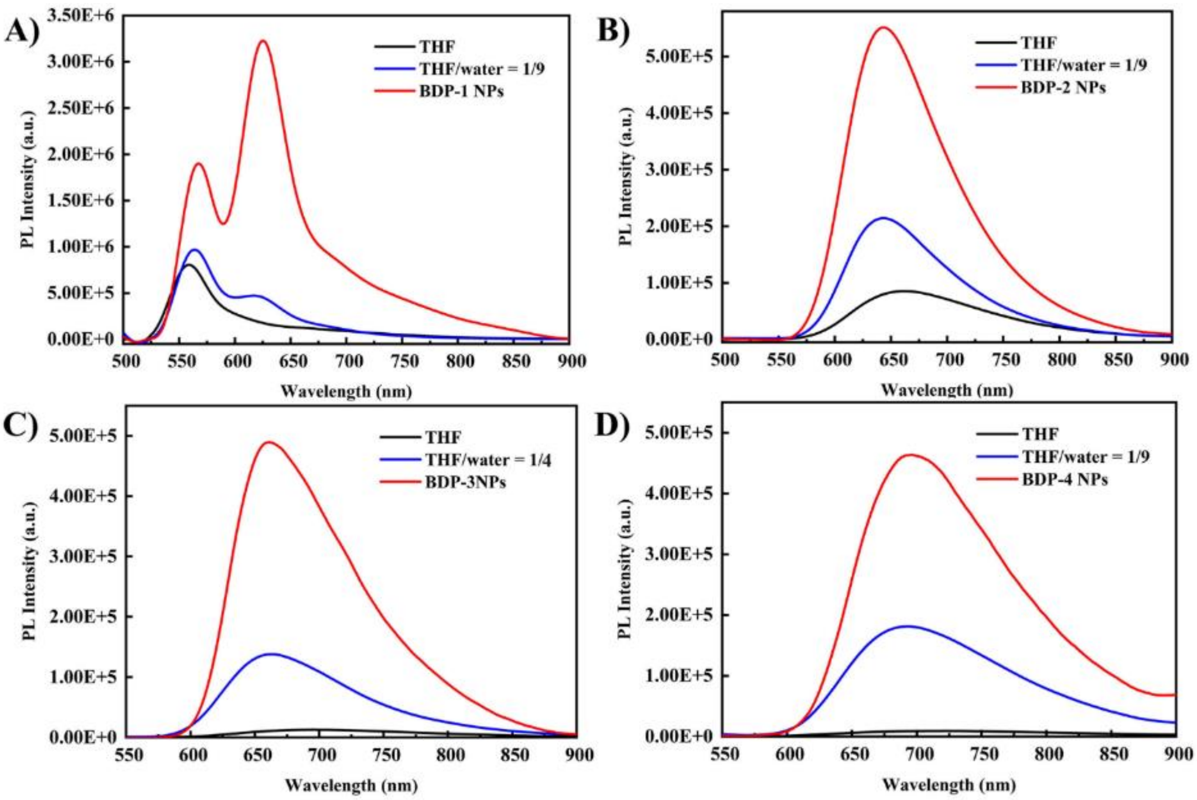

Figure S27. A) Emission spectra of **BDP-1** (10^{-5} M) in THF, THF/water (v:v) = 1/9, and **BDP-1** NPs in water. B) Emission spectra of **BDP-2** (10^{-5} M) in THF, THF/water (v:v) = 1/9, and **BDP-2** NPs in water. C) Emission spectra of **BDP-3** (10^{-5} M) in THF, THF/water (v:v) = 1/4, and **BDP-3** NPs in water. D) Emission spectra of **BDP-4** (10^{-5} M) in THF, THF/water (v:v) = 1/9, and **BDP-4** NPs in water.

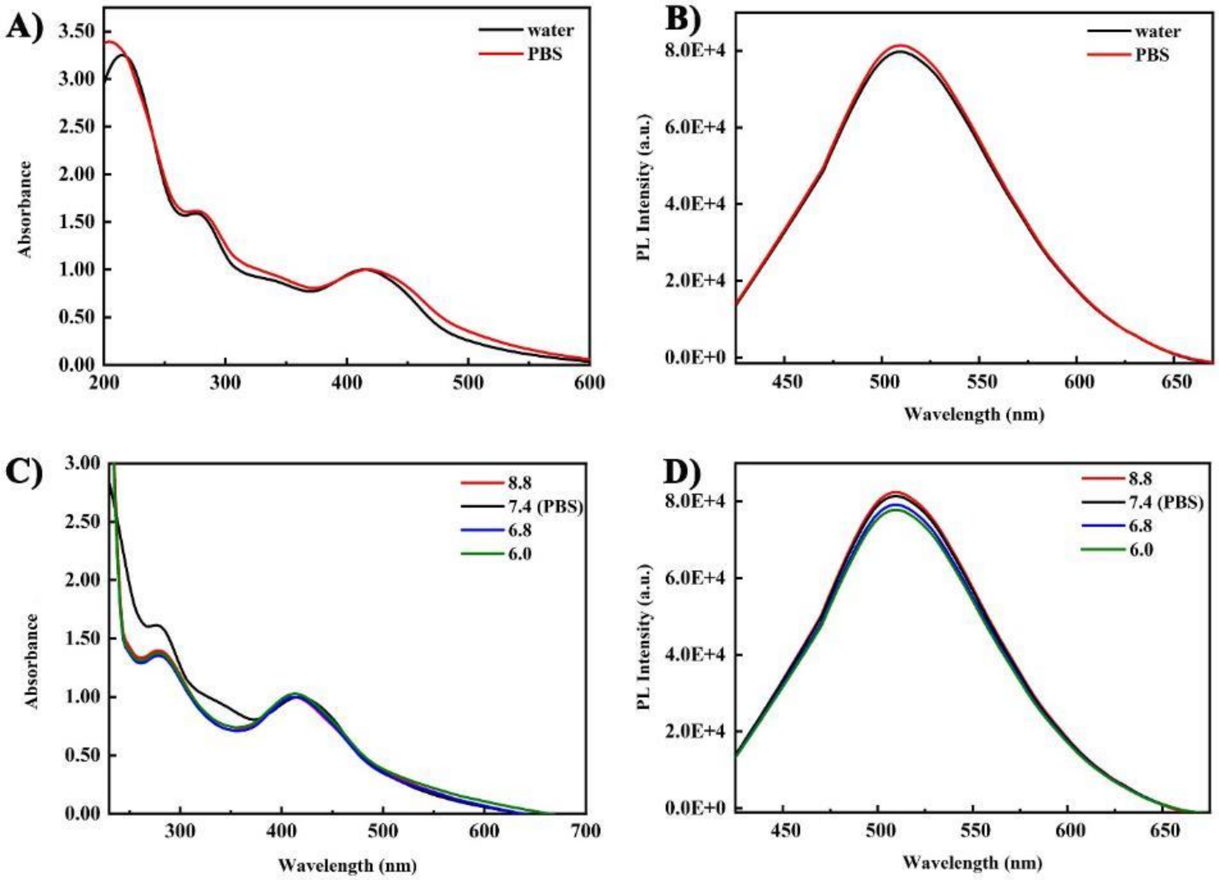

Figure S28. A) UV-vis absorption and B) emission spectra of **Cur-CL** NPs (10^{-5} M) in water and PBS at room temperature. C) UV-vis absorption and D) emission spectra of **Cur-CL** NPs (10^{-5} M) in PBS at different pH values (6.0 - 8.8).

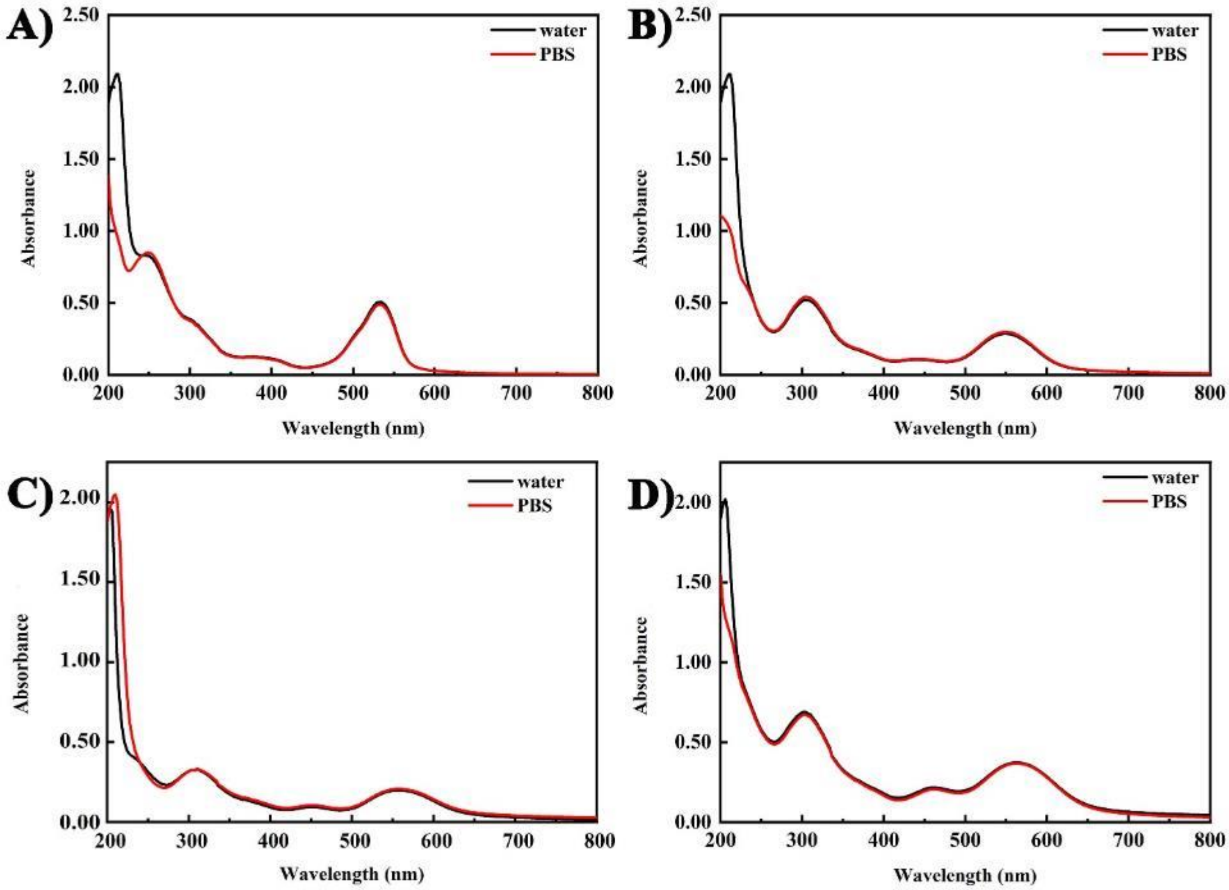

Figure S29. UV-vis absorption spectra of A) **BDP-1** NPs, B) **BDP-2** NPs, C) **BDP-3** NPs and D) **BDP-4** NPs (10^{-5} M) in water and PBS at room temperature.

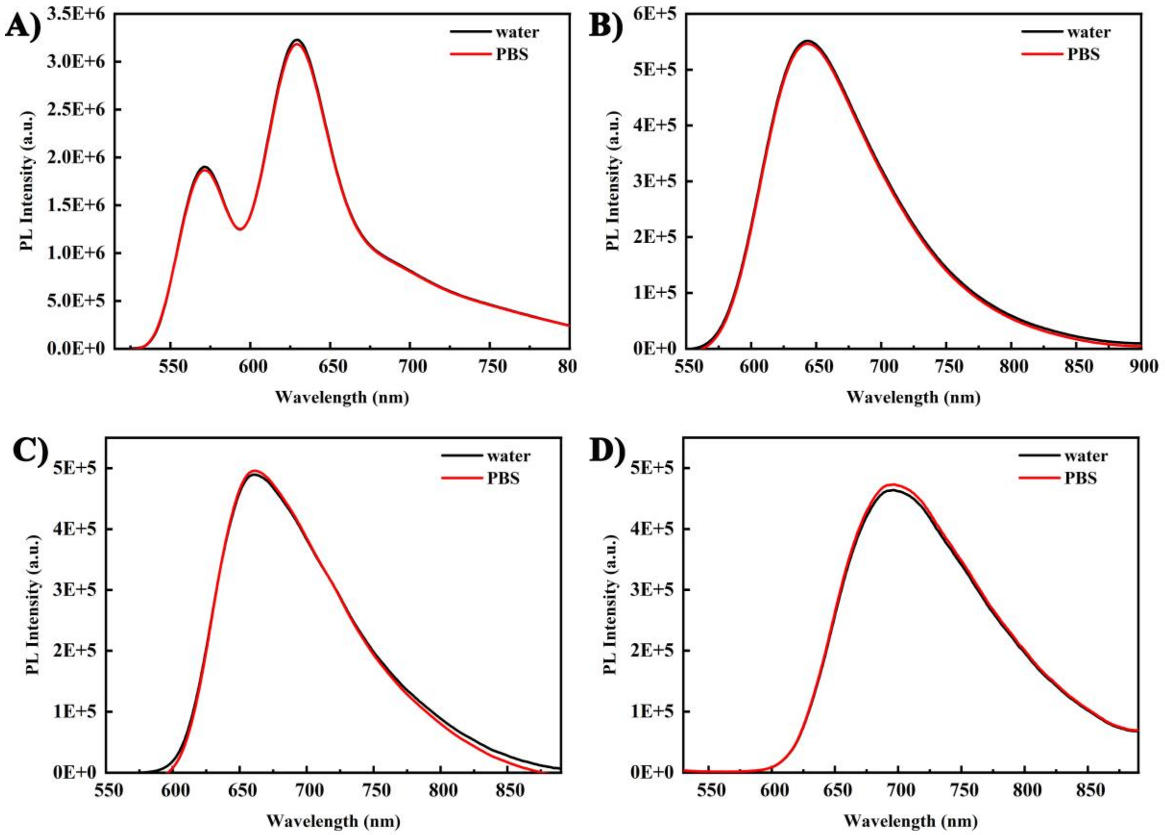

Figure S30. Emission spectra of A) **BDP-1** NPs, B) **BDP-2** NPs, C) **BDP-3** NPs and D) **BDP-4** NPs (10^{-5} M) in water and PBS at room temperature.

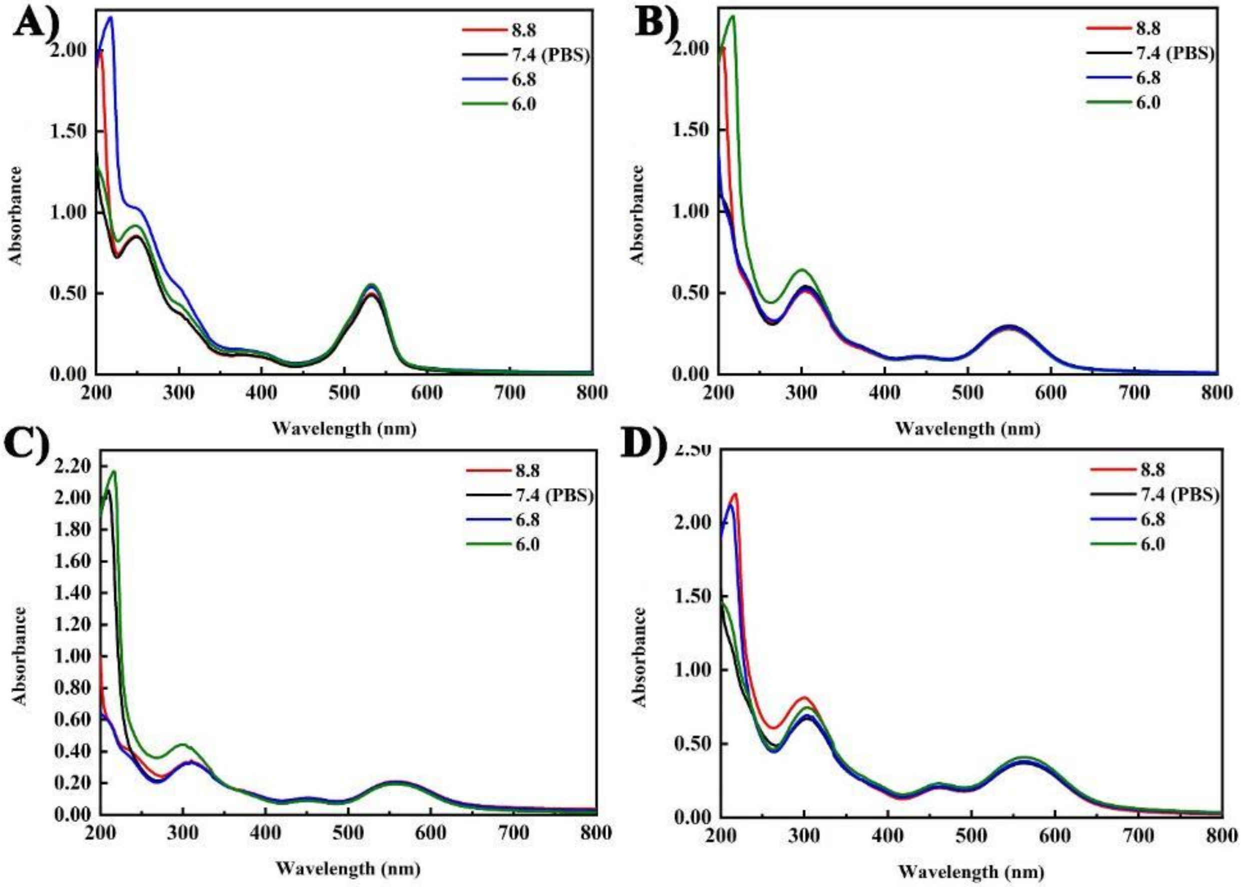

Figure S31. UV-vis absorption spectra of A) **BDP-1** NPs, B) **BDP-2** NPs, C) **BDP-3** NPs and D) **BDP-4** NPs (10^{-5} M) in PBS at different pH values (6.0 - 8.8).

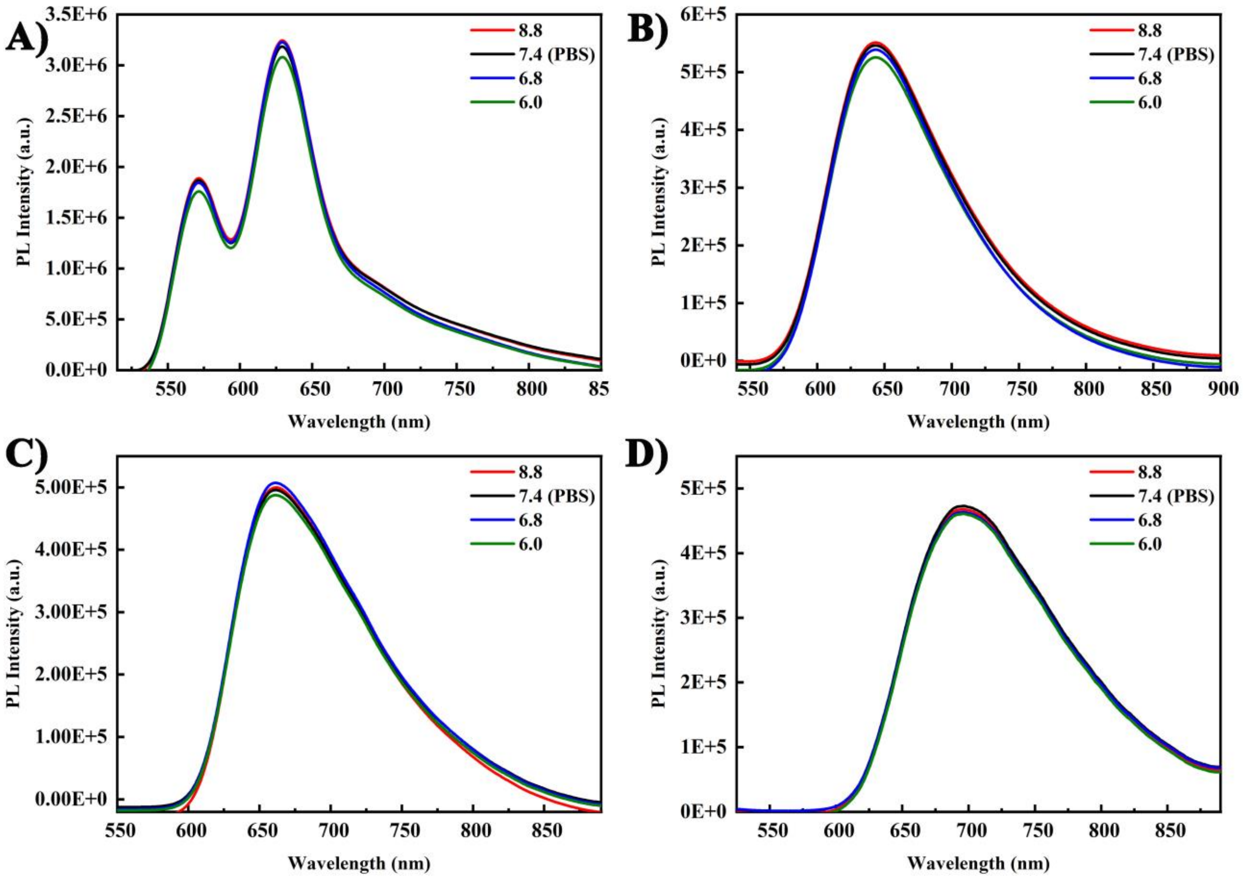

Figure S32. Emission spectra of A) **BDP-1** NPs, B) **BDP-2** NPs, C) **BDP-3** NPs and D) **BDP-4** NPs (10^{-5} M) in PBS at different pH values (6.0 - 8.8).

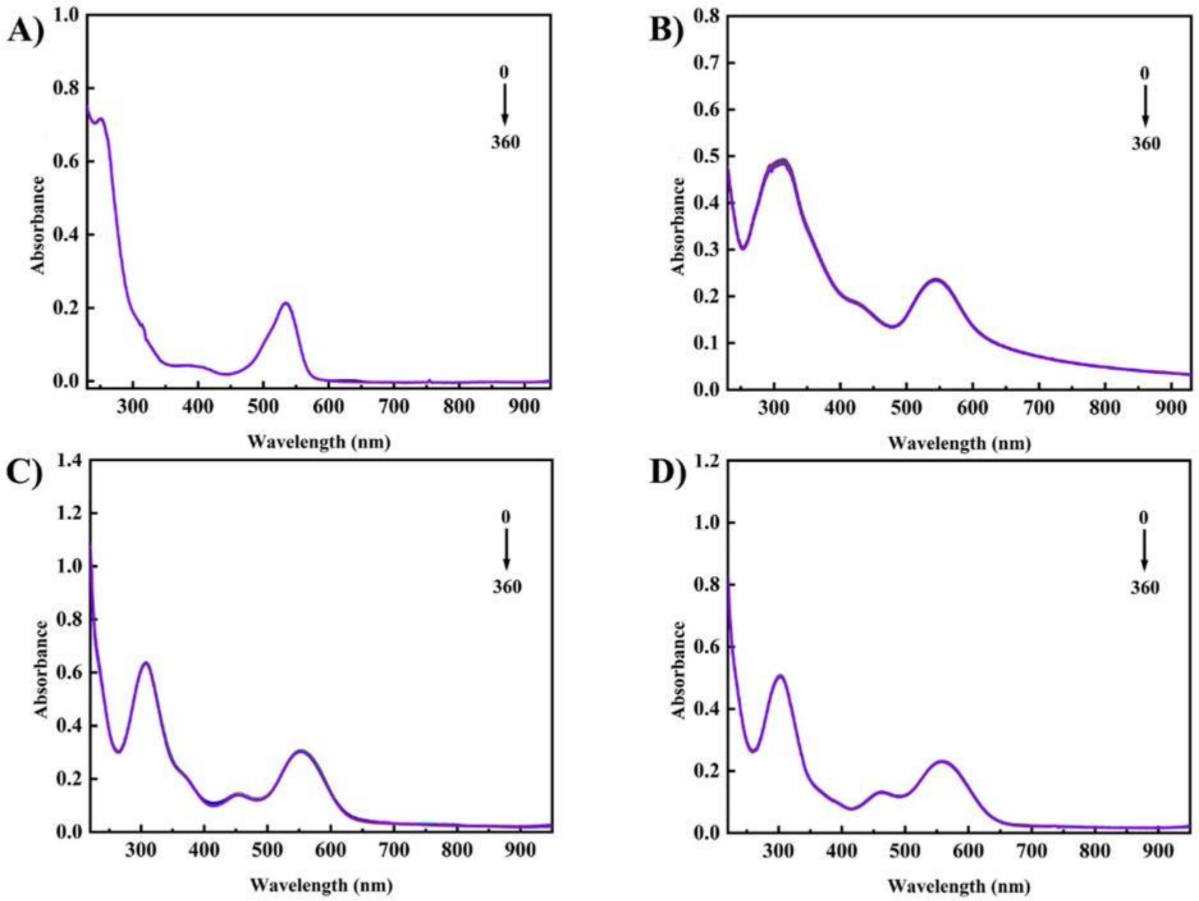

Figure S33. UV–vis absorption spectra changes of A) **BDP-1** NPs, B) **BDP-2** NPs, C) **BDP-3** NPs, D) **BDP-4** NPs (10^{-5} M) for different times (0-360 s) under white-light irradiation (20 mW cm^{-2}).

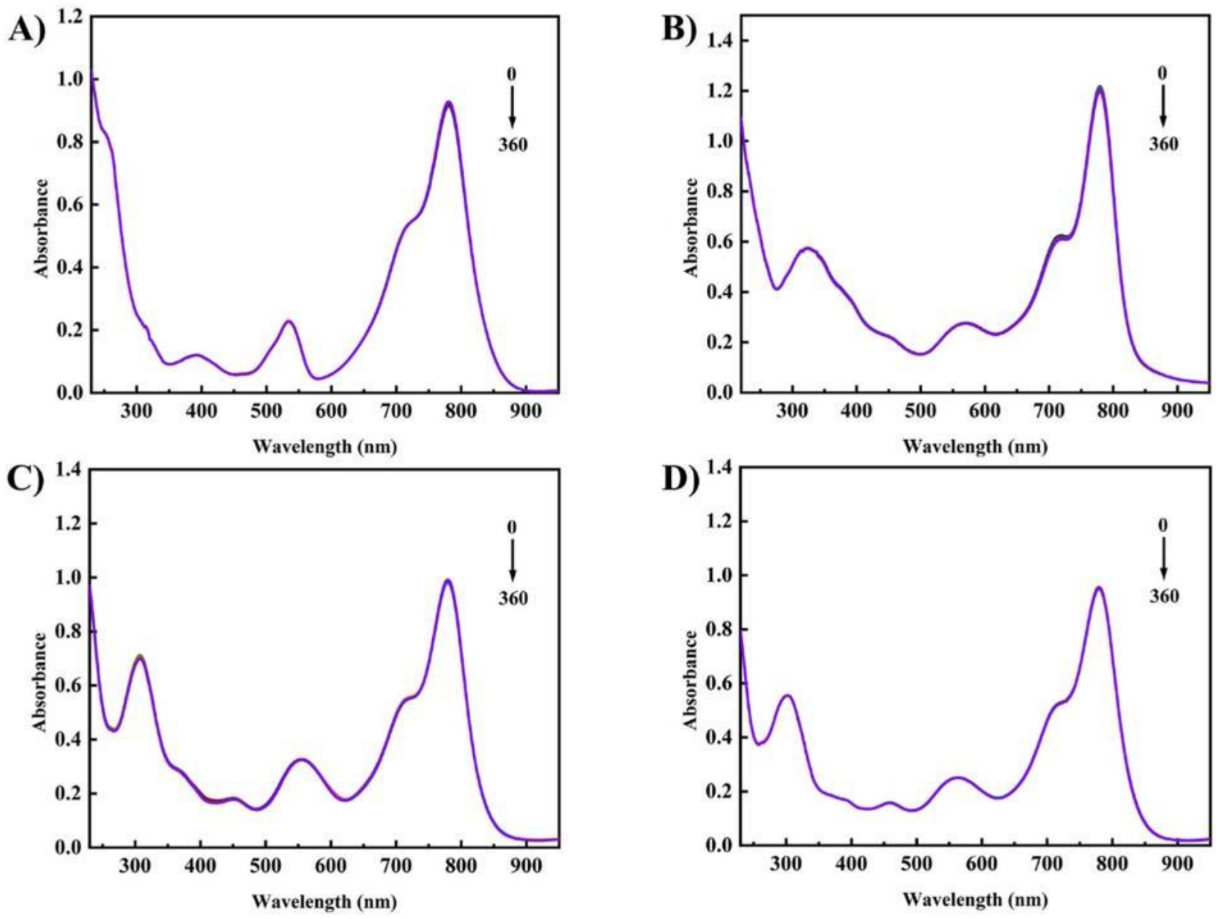

Figure S34. UV–vis absorption spectra changes of ICG (6.5×10^{-6} M, 790 nm) in the presence of A) **BDP-1** NPs, B) **BDP-2** NPs, C) **BDP-3** NPs, D) **BDP-4** NPs (10^{-5} M) for different times (0-360 s).

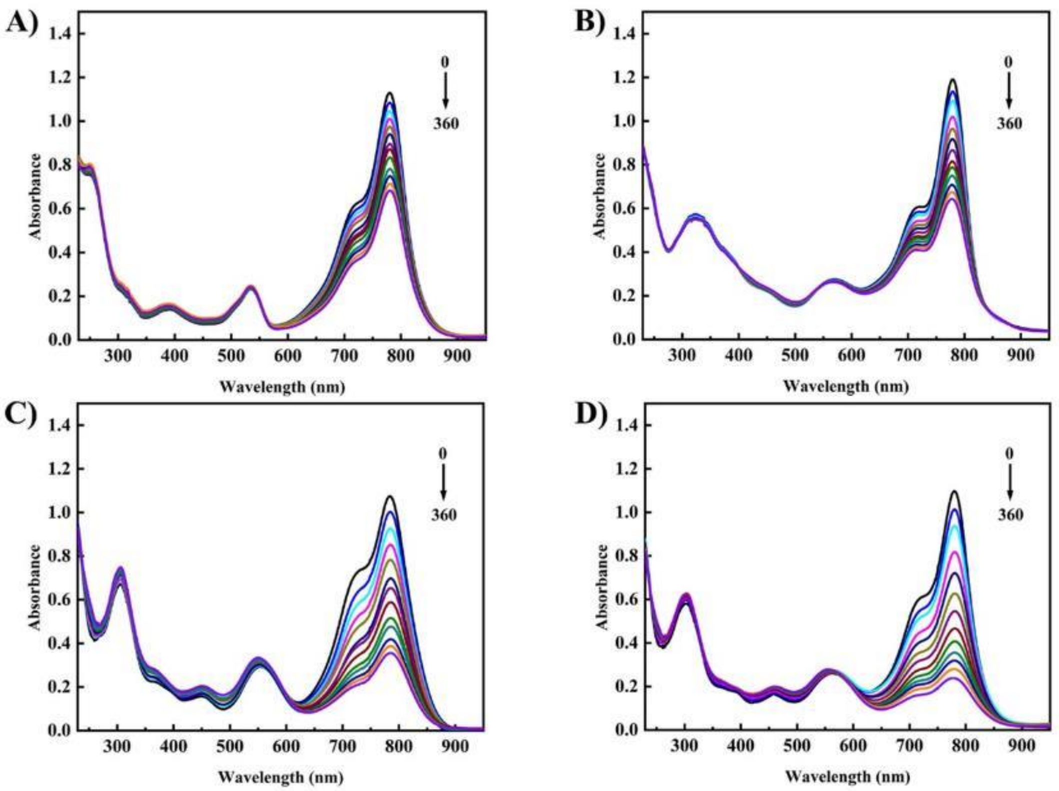

Figure S35. UV–vis absorption spectra changes of ICG (6.5×10^{-6} M, 790 nm) in the presence of A) **BDP-1** NPs, B) **BDP-2** NPs, C) **BDP-3** NPs, D) **BDP-4** NPs (10^{-5} M) for different times (0-360 s under white-light irradiation (20 mW cm^{-2}).

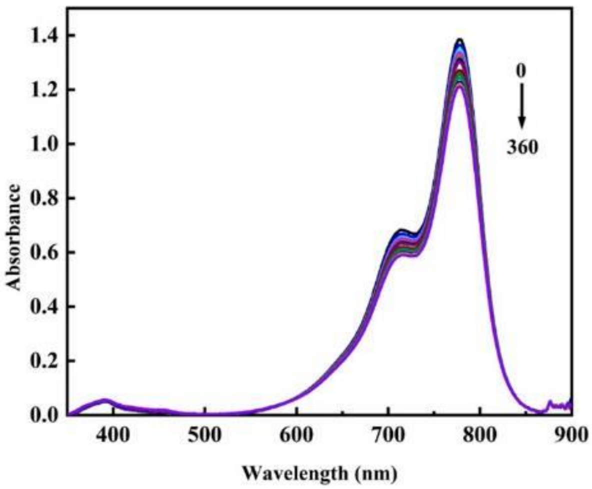

Figure S36. UV–vis absorption spectra of ICG (6.5×10^{-6} M, 790 nm) for different times (0-360 s) under white-light irradiation (20 mW cm^{-2}).

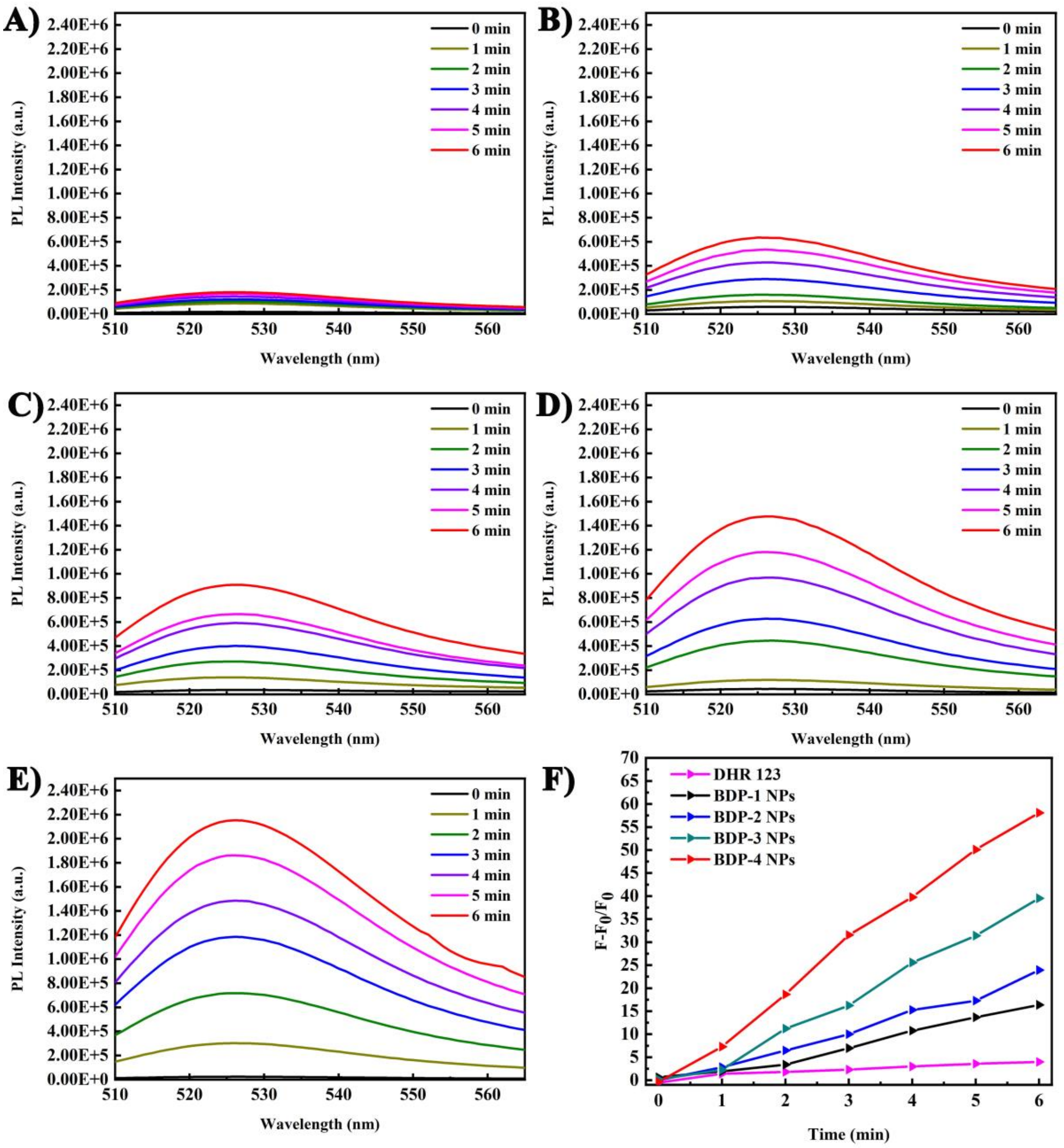

Figure S37. Emission spectral changes of DHR123 (10^{-5} M) in the presence of A) only DHR123, B) **BDP-1** NPs, C) **BDP-2** NPs, D) **BDP-3** NPs and E) **BDP-4** NPs (10^{-5} M) for different times (0-6 min) under white-light irradiation (20 mW cm^{-2}). F) Plot of the relative emission intensity (F-F_0/F_0) of DHR123 (10^{-5} M) solution containing different NPs (10^{-5} M) under white-light irradiation (20 mW cm^{-2}). F_0 = emission of DHR123 (526 nm) without irradiation. F = real-time emission of DHR123 (526 nm) with different irradiation times.

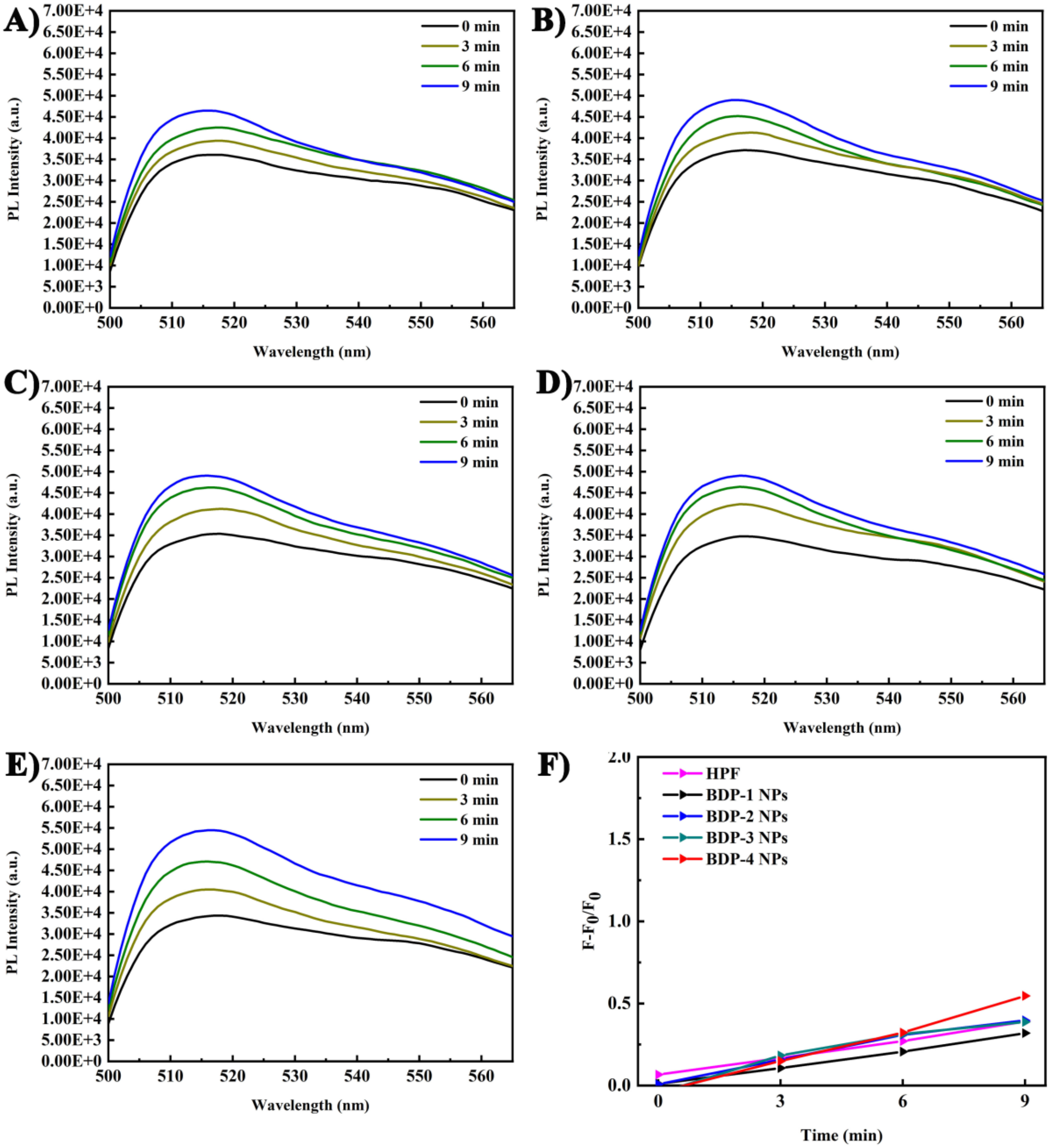

Figure S38. Emission spectral changes of HPF (10^{-5} M) in the presence of A) only HPF, B) **BDP-1** NPs, C) **BDP-2** NPs, D) **BDP-3** NPs and E) **BDP-4** NPs (10^{-5} M) for different times (0-9 min under white-light irradiation (20 mW cm^{-2}). F) Plot of the relative emission intensity (F-F_0/F_0) of DHR123 (10^{-5} M) solution containing different NPs (10^{-5} M) under white-light irradiation (20 mW cm^{-2}). F_0 = emission of HPF (516 nm) without irradiation. F = real-time emission of HPF (516 nm) with different irradiation times.

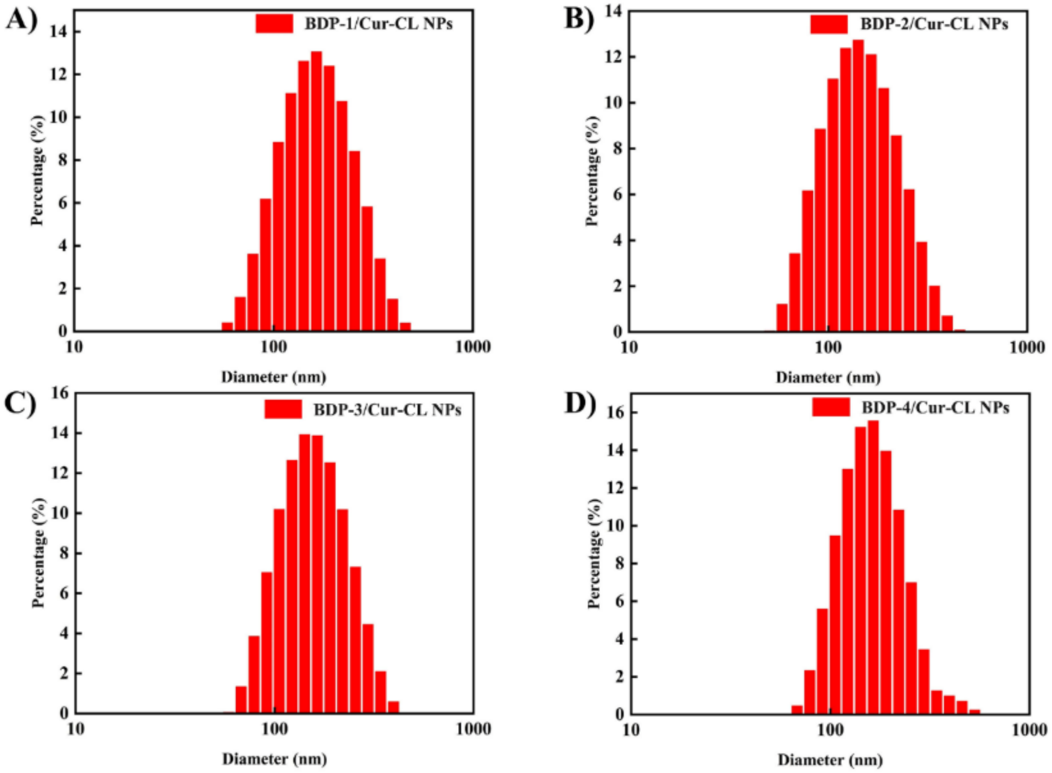

Figure S39. Dynamic laser scattering results of A) **BDP-1/Cur-CL** NPs, B) **BDP-2/Cur-CL** NPs, C) **BDP-3/Cur-CLBDP-3/Cur-CL** NPs, D) **BDP-4/Cur-CL** NPs (10^{-5} M) in water.

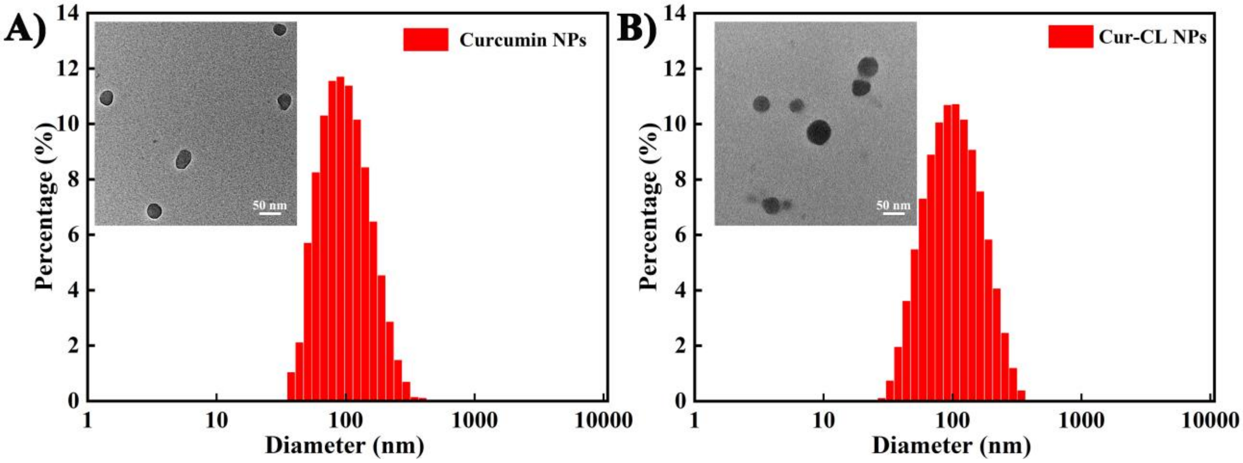

Figure S40. The TEM images and dynamic laser scattering results of A) Curcumin NPs , B) **Cur-CL** NPs (10^{-5} M) in water.

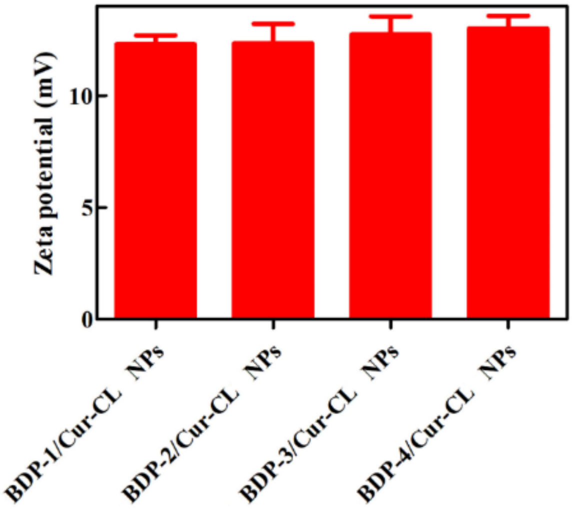

Figure S41. Zeta potential of different NPs (10^{-5} M) in water.

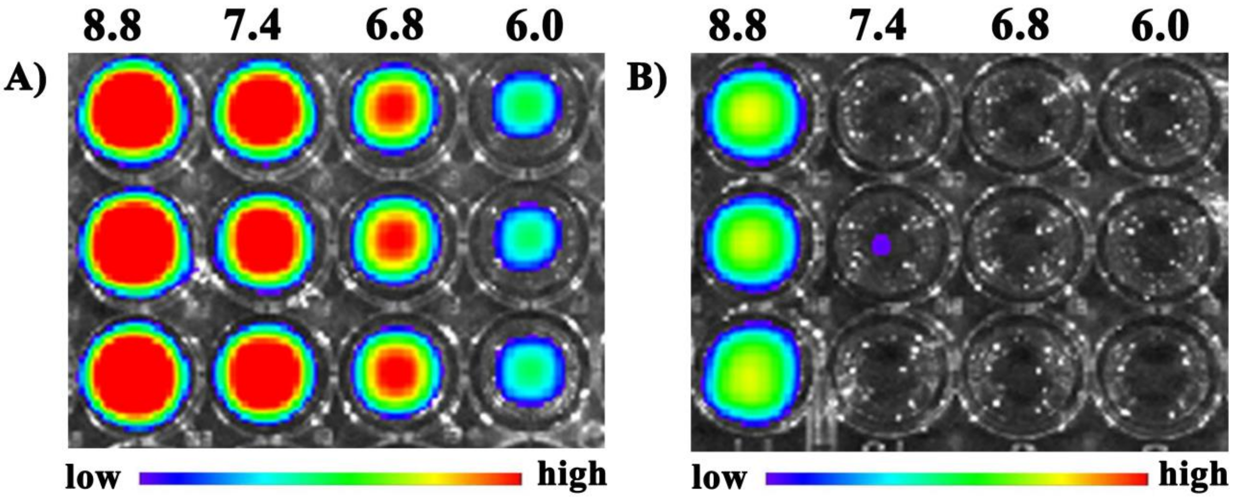

Figure S42. Afterglow luminescence images of pre-irradiated **BDP-4/Cur-CL** NPs (100 μg/mL based on **Cur-CL**) in the presence of A) $ONOO^-$ and B) H_2O_2 (200 μM) in PBS at different pH values (6.0 - 8.8).

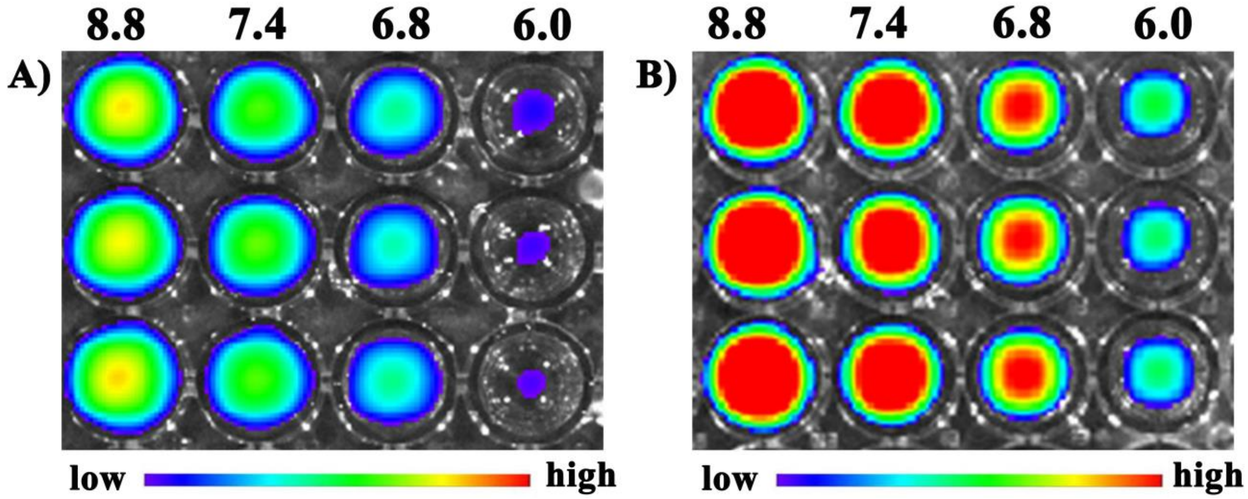

Figure S43. Afterglow luminescence images of pre-irradiated A) **Cur-CL** NPs and B) **BDP-4/Cur-CL** NPs (100 μg/mL based on **Cur-CL**) in the presence of $ONOO^-$ (200 μM) in PBS at different pH values (6.0 - 8.8).

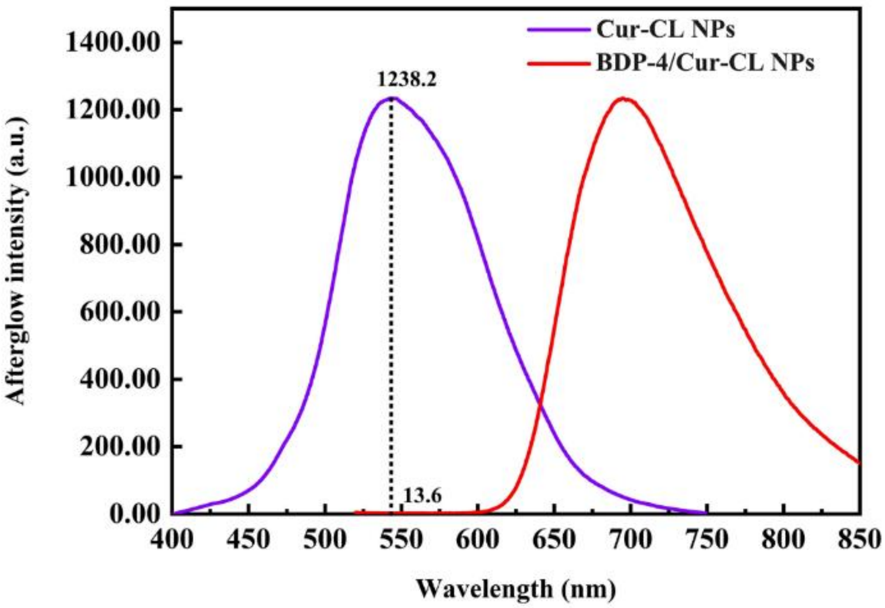

Figure S44. Chemiluminescence (CL) intensity curves of the **Cur-CL** NPs and **BDP-4/Cur-CL** NPs pre-irradiated (0.25 W cm^{-2}, 15 min) at 10 min post $ONOO^-$ (200 μM) addition. The afterglow emission curves were measured by a FS5 spectrofluorometer (Edinburgh Instrument) without excitation.

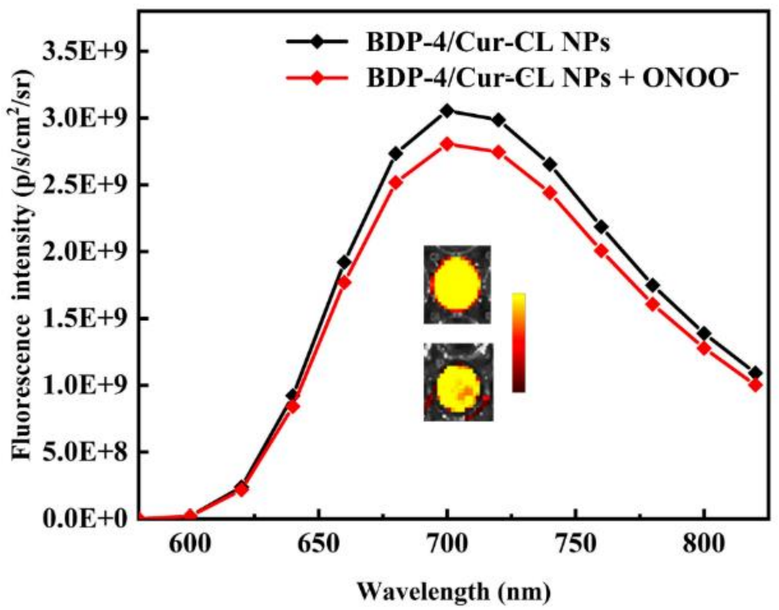

Figure S45. Fluorescence spectra of **BDP-4/Cur-CL** NPs (100 μg/mL based on **Cur-CL**) in the absence or presence of $ONOO^-$ (200 μM) in PBS solution (pH 7.4). Insets: the corresponding fluorescence images acquired on an IVIS imaging system.

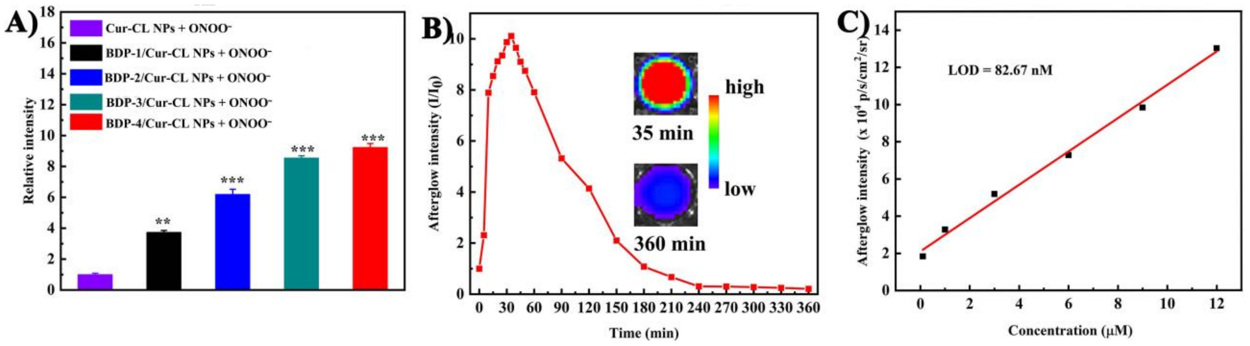

Figure S46. A) Afterglow luminescence intensities of the pre-irradiated **Cur-CL** NPs, **BDP-1/Cur-CL** NPs, **BDP-2/Cur-CL** NPs, **BDP-3/Cur-CL** NPs and **BDP-4/Cur-CL** NPs upon addition of $ONOO^-$ (200 μM) in PBS solution (pH 7.4). $^{*}p < 0.05$, $^{**}p < 0.01$, and $^{***}p < 0.001$ vs **Cur-CL** NPs treated group. B) Decay of afterglow luminescence intensity after addition of $ONOO^-$ (200 μM) to the pre-irradiated **BDP-4/Cur-CL** NPs in PBS (pH 7.4). C) Afterglow luminescence intensities of **BDP-4/Cur-CL** NPs in PBS solution (pH 7.4) after addition of $ONOO^-$.

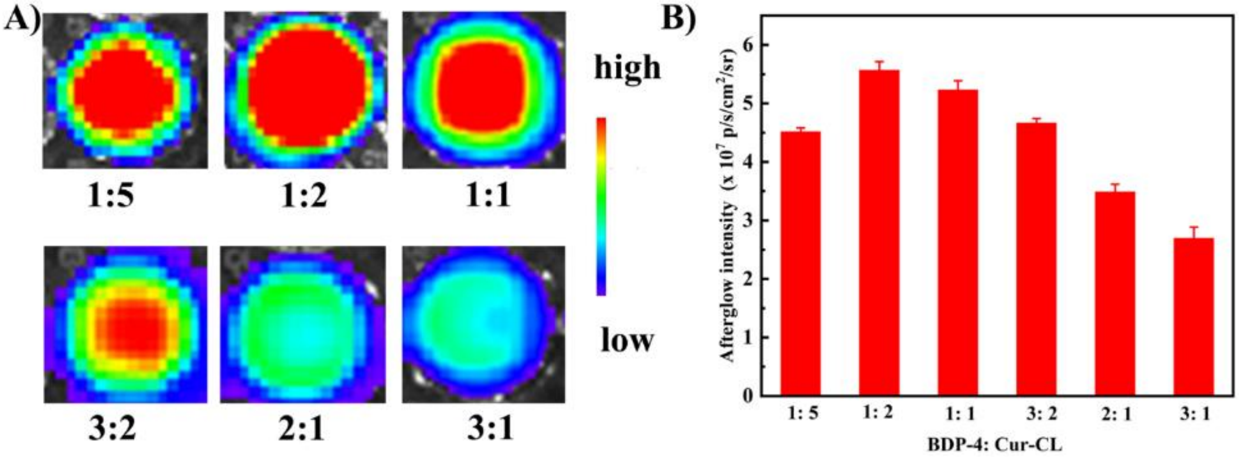

Figure S47. Optimization of the doping ratio for **BDP-4/Cur-CL** NPs. (A) Afterglow images and (B) quantitative analysis of the afterglow signals of the **BDP-4/Cur-CL** NPs (100 μg/mL based on **Cur-CL**) after light pre-irradiation (0.25 W/cm^2) for 15 min in 1×PBS containing $ONOO^−$ (200 μM). Error bars, mean ± SD (n = 3).

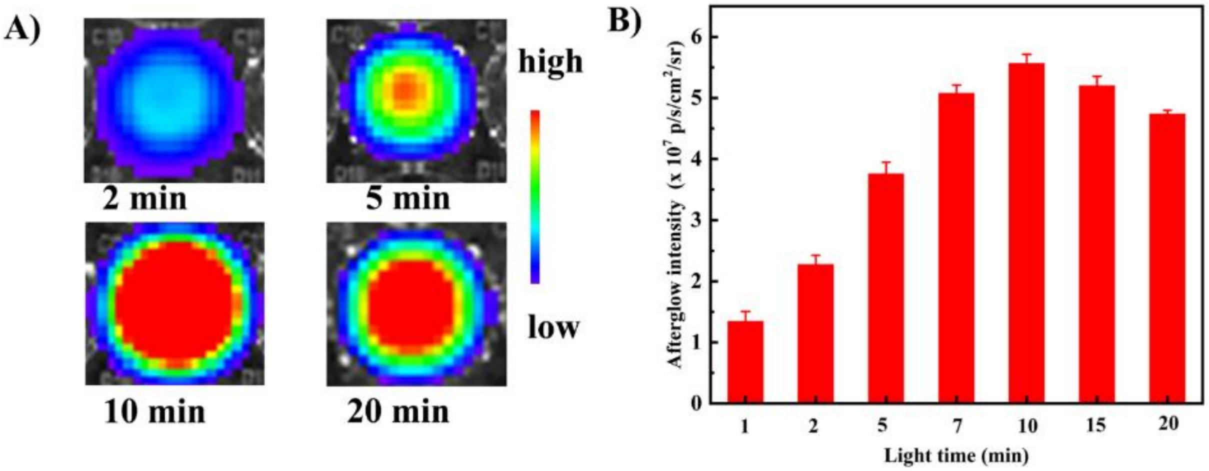

Figure S48. Optimization of the light pre-irradiation time for **BDP-4/Cur-CL** NPs. (A) Afterglow images and (B) quantitative analysis of the afterglow signals of the **BDP-4/Cur-CL** NPs (100 μg/mL based on **Cur-CL**) after light pre-irradiation (0.25 W/cm^2) for 15 min in 1×PBS containing $ONOO^−$ (200 μM). Error bars, mean ± SD (n = 3).

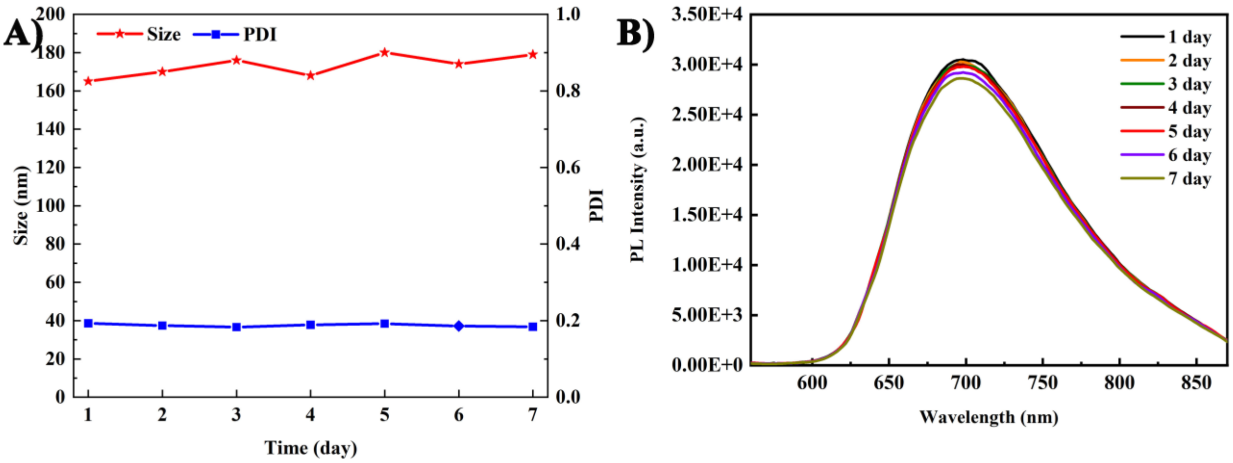

Figure S49. A) Size changes of pre-irradiated **BDP-4/Cur-CL NPs** (10^{-5} M) in PBS solution (pH 7.4) during 7 days. B) Fluorescence spectra of pre-irradiated **BDP-4/Cur-CL** NPs (10^{-5} M) in PBS solution (pH 7.4) during 7 days.

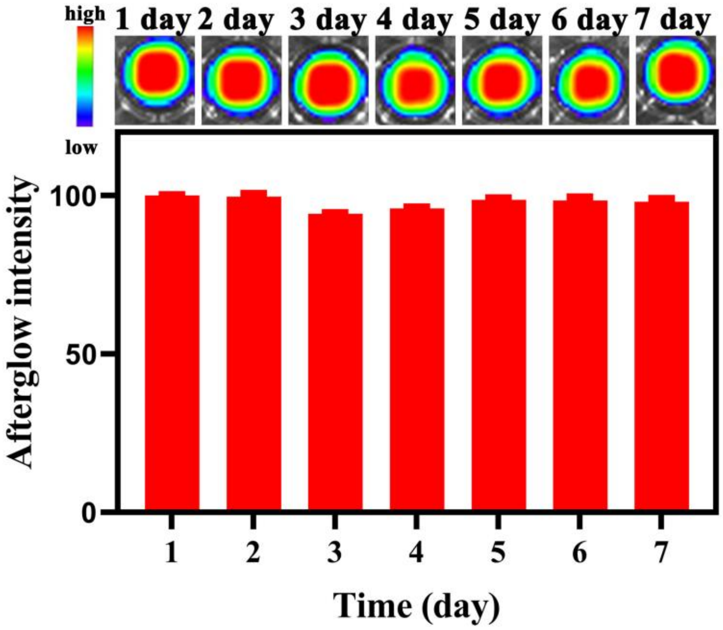

Figure S50. Afterglow luminescence imaging of pre-irradiated **BDP-4/Cur-CL** NPs (100 μg/mL based on **Cur-CL**) adds $ONOO^{-}$ (200 μM) at different time in PBS solution (pH 7.4).

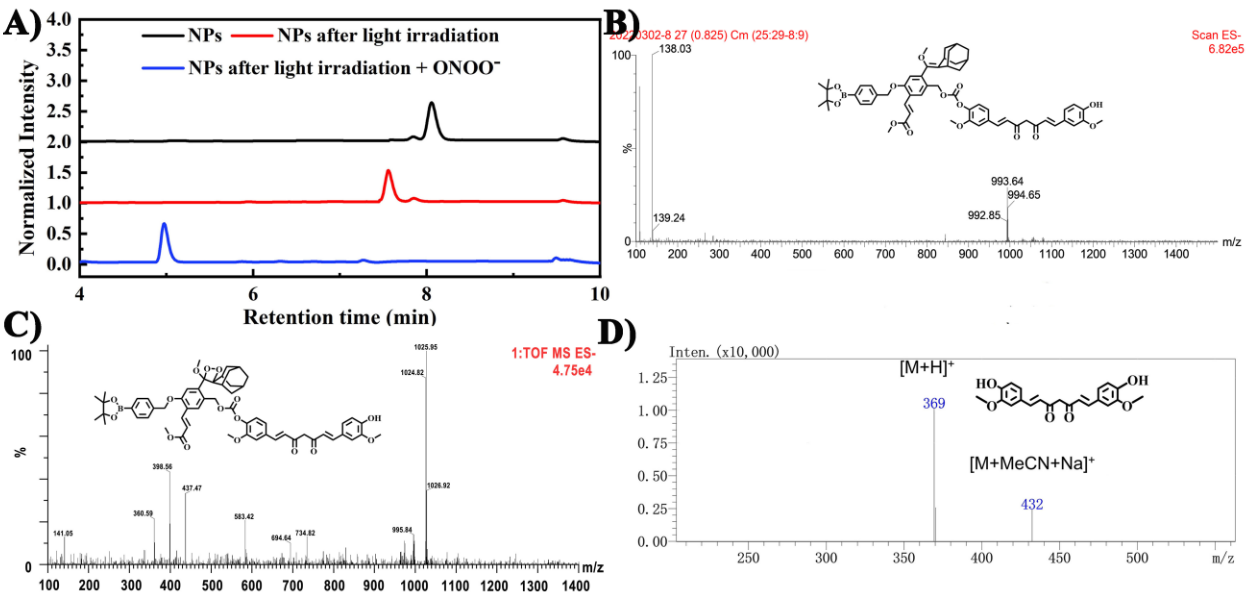

Figure S51. A) Ultra performance liquid chromatography (UPLC) results of **BDP-4/Cur-CL** NPs after irradiation and ONOO⁻ treatment, respectively. The corresponding HRMS spectrum: B) NPs, C) NPs after irradiation and D) NPs after irradiation and ONOO⁻ treatment.

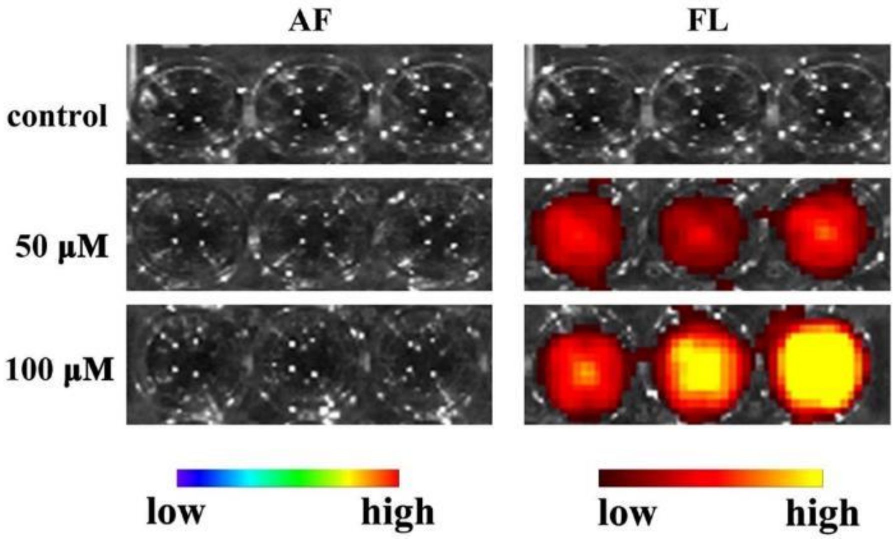

Figure S52. Afterglow (AF) and fluorescence (FL) luminescence cell imaging of **BDP-4/Cur-CL** NPs when present in HT22 culture.

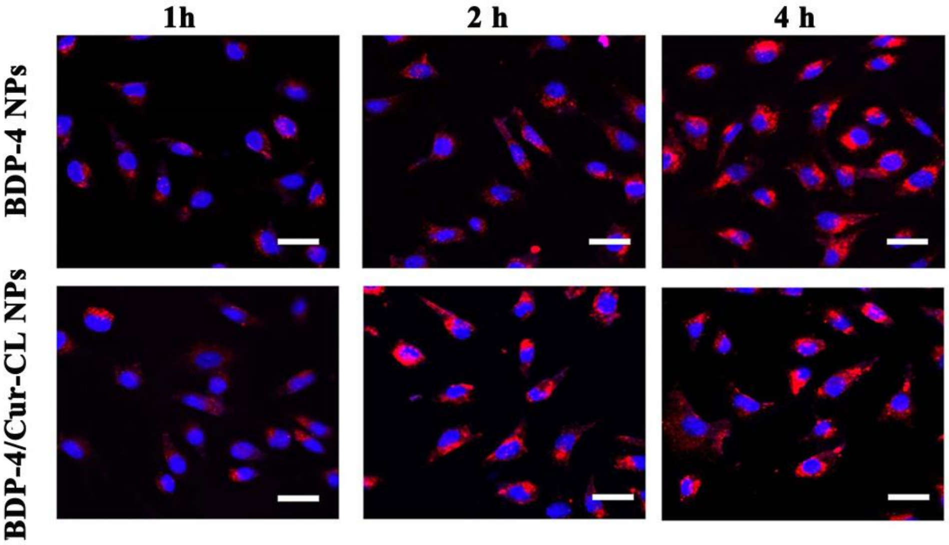

Figure S53. CLSM images of HT22 cells incubated with **BDP-4** NPs and **BDP-4/Cur-CL** NPs (20 μg mL^{-1}) for different times: the scale bars are 20 μm.

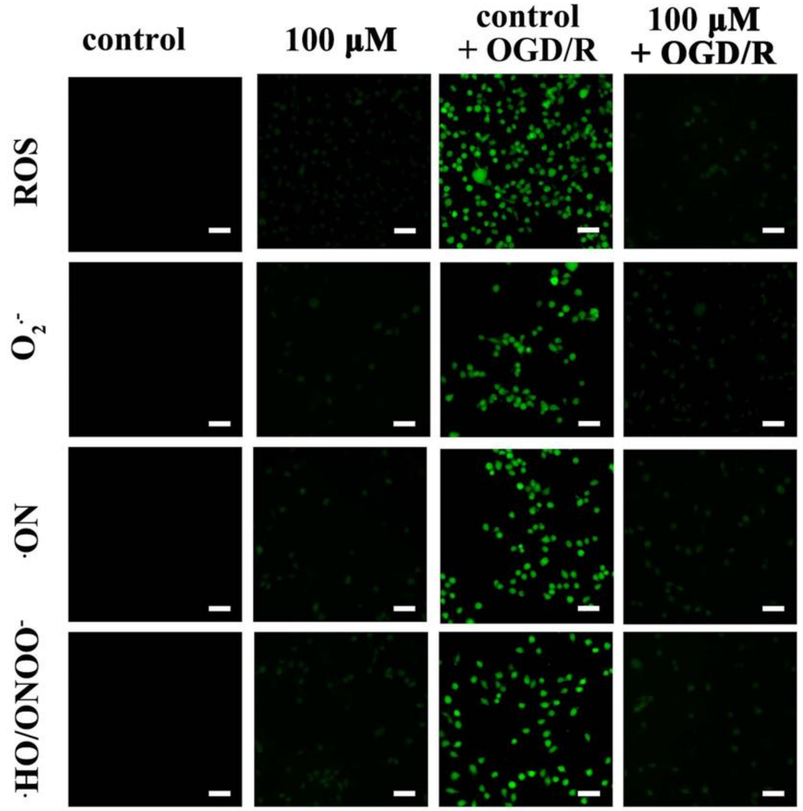

Figure S54. BDP-4/Cur-CL NPs decreased RONS activity induced by OGD/R-treated HT22 cells *in vitro*, the scale bars are 100 μm.

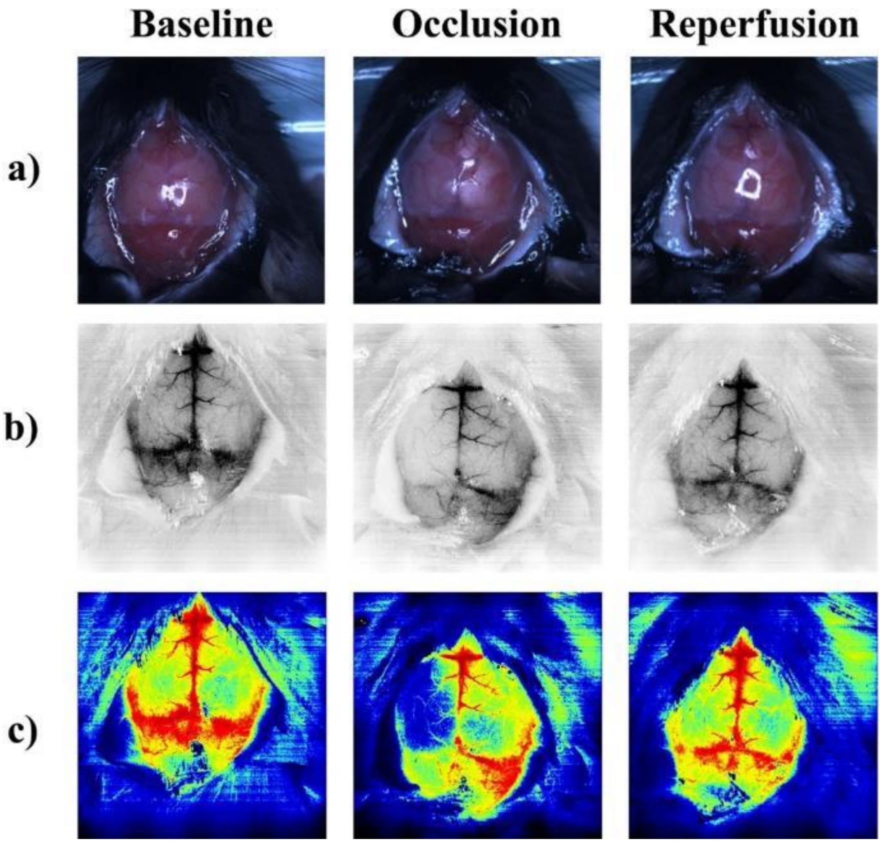

Figure S55. A) Representative images of mice, B) the white-light image of transparent bone window observation and C) laser speckle contrast images for CBF in MCAO stroke.

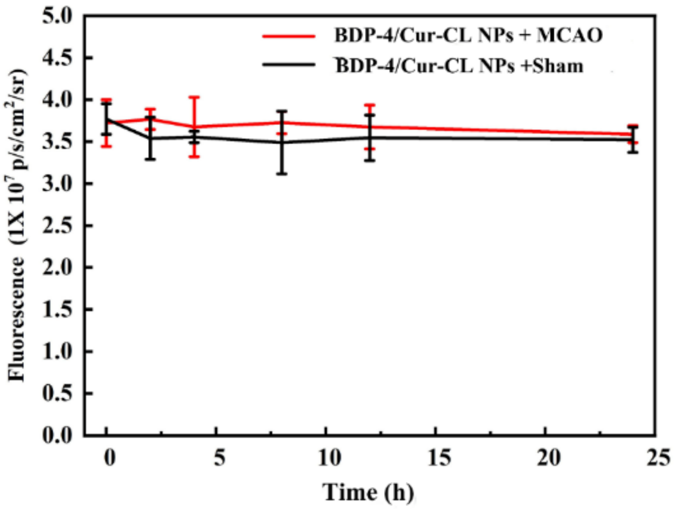

Figure S56. Fluorescent intensities for different groups mice after injection of the pre-irradiated **BDP-4/Cur-CL** NPs.

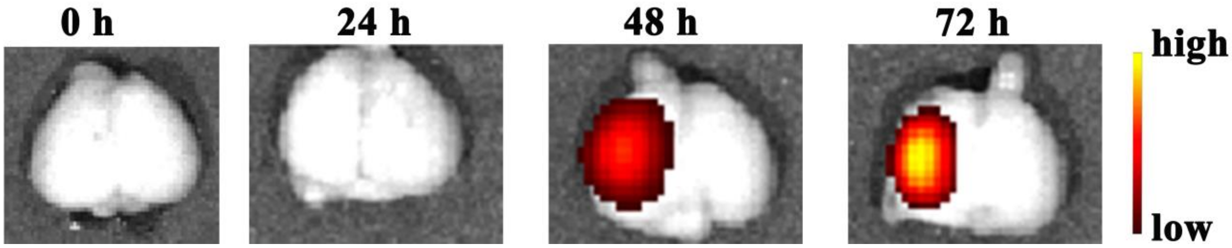

Figure S57. The fluorescence imaging data of **BDP-4/Cur-CL** NPs (2 mg mL^{-1}, 100 μL, based on curcumin) *in vivo* after intravenous injection. The images show that the nanoparticles could penetrate the blood-brain barrier (BBB) at the ischemia-reperfusion injury site after 48 h.

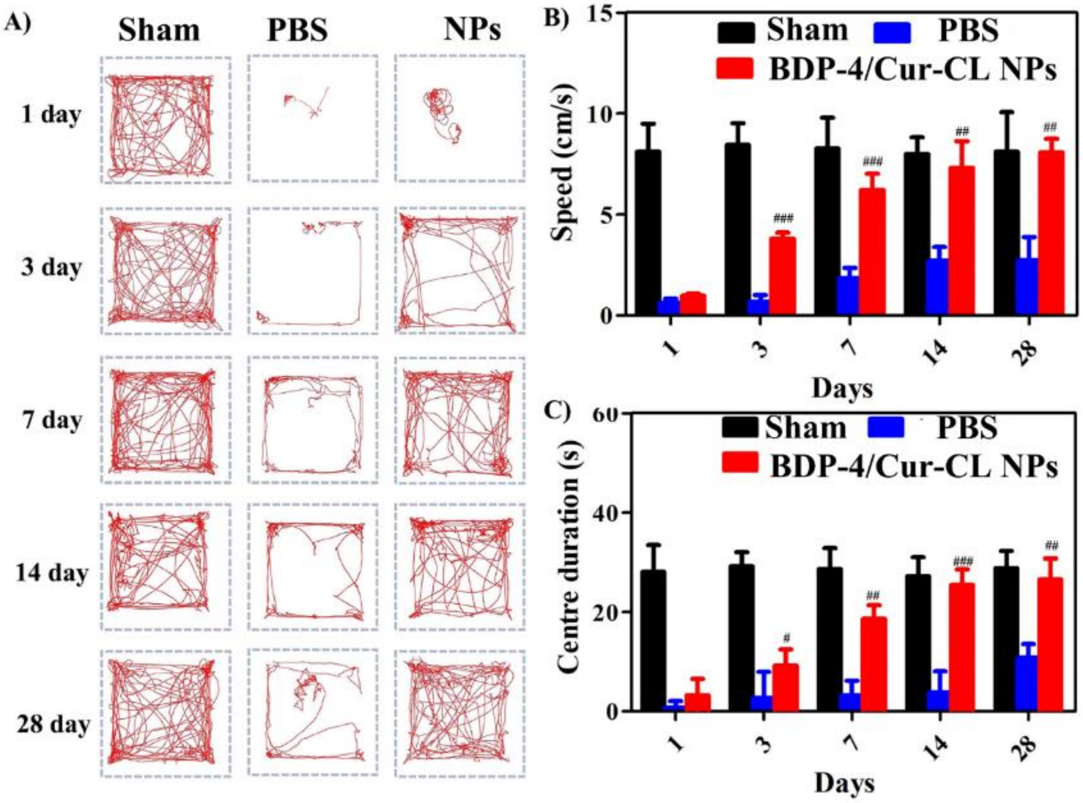

Figure S58. Open field test for different groups of mice at 1, 3, 7, 14 and 28 days. A) The movement trajectory of the mice. B) Average movement speed of the mice. C) Time of mice entry to the center. Error bars, mean ± SD (n = 10). $^{\#}p < 0.05$, $^{\#\#}p < 0.01$, and $^{\#\#\#}p < 0.001$ vs PBS treated group.

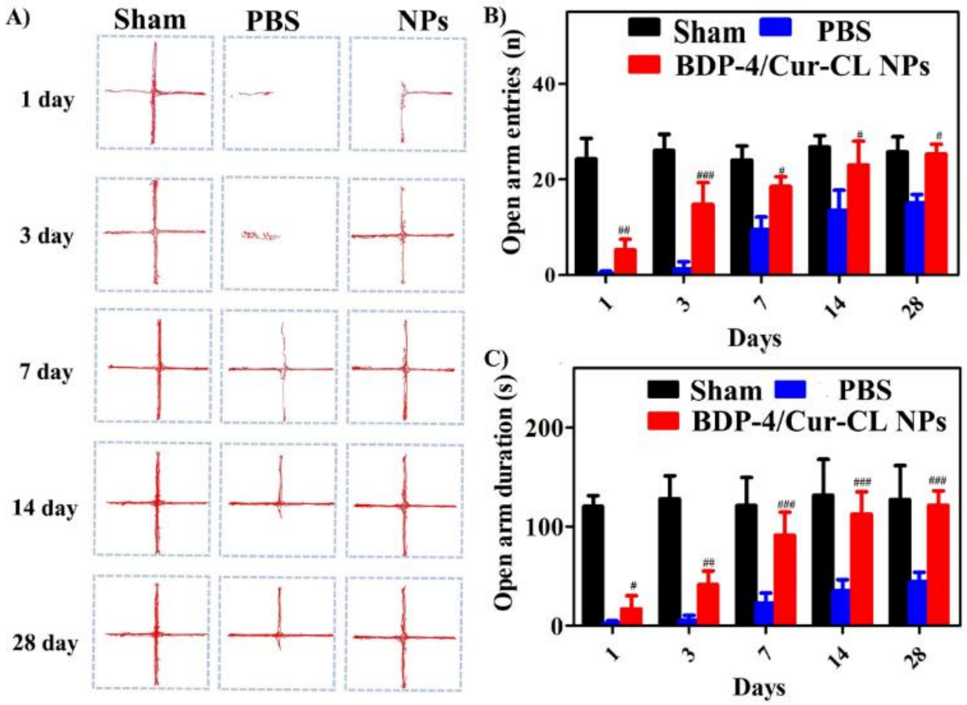

Figure S59. Elevated plus maze test in different groups of mice at 1, 3, 7, 14 and 28 days. A) The movement trajectory of the mice. B) Times of mice entry to open arm entry. C) Time of mice entry to open arm time. Error bars, mean ± SD (n = 10). $^{\#}p < 0.05$, $^{\#\#}p < 0.01$, and $^{\#\#\#}p < 0.001$ vs PBS treated group.

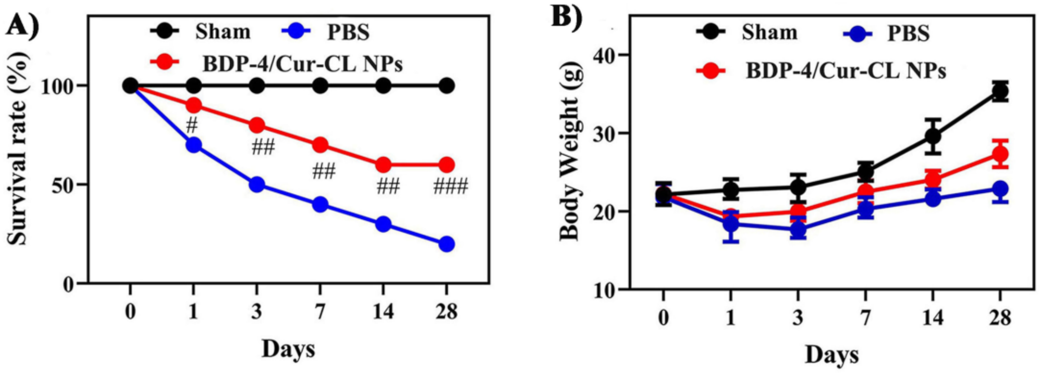

Figure S60. A) Survival rate for sham and IS groups at 1, 3, 7, 14 and 28 days. B) Body weights for different groups of mice. Error bars, mean ± SD (n = 6). $^{\#}p < 0.05$, $^{\#\#}p < 0.01$, and $^{\#\#\#}p < 0.001$ vs PBS treated group.

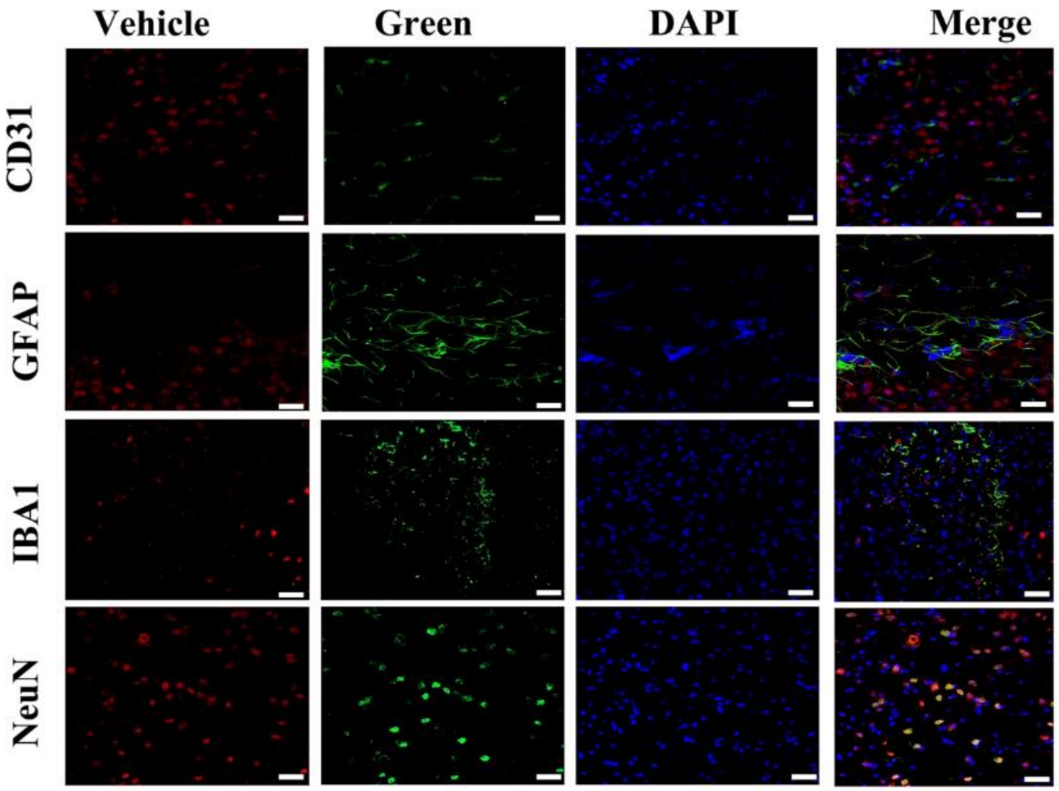

Figure S61. Targeting of **BDP-4/Cur-CL** NPs (red) in ischemic stroke. Mice were subjected to a 30 min occlusion. Brain sections were harvested at 72 h ischemic stroke and stained with CD31 (green), GFAP (green), IBA1 (green) and NeuN (green). Scale bar represents 100 μm (n = 6 per group).

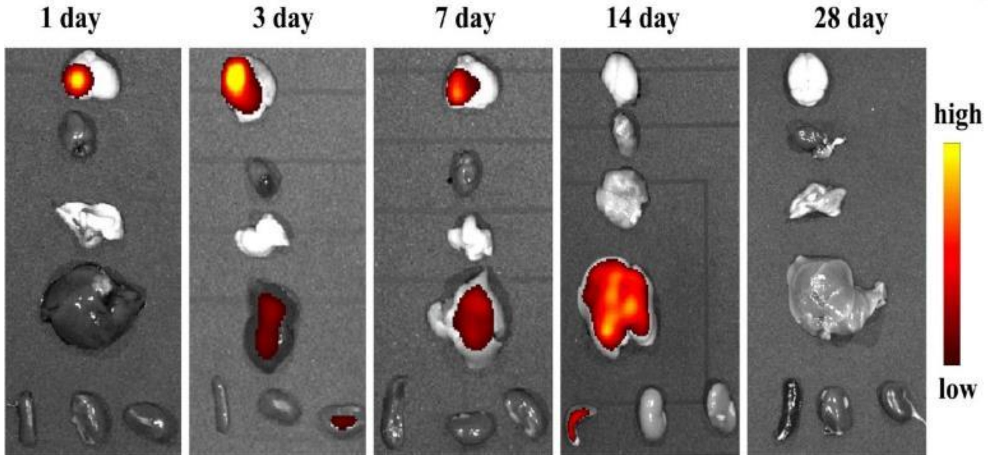

Figure S62. The fluorescence intensity of **BDP-4/Cur CL** NPs in MCAO stroke mice at different time in different organs (brain, heart, liver, spleen, kidney and lung).

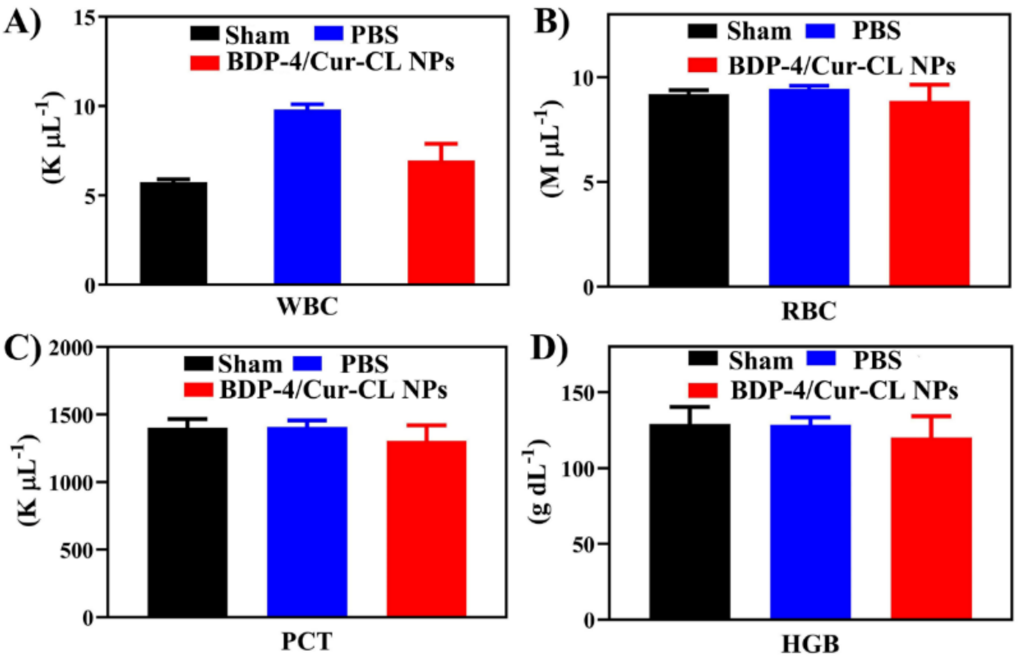

Figure S63. Blood test parameters regarding to A) WBC, B) RBC, C) PCT and D) HGB blood cell count of the mice with different treatment at 3 days. Error bars, mean ± SD (n = 3). (WBC = white blood cell; RBC = red blood cell; PCT = procalcitonin; HGB = haemoglobin).

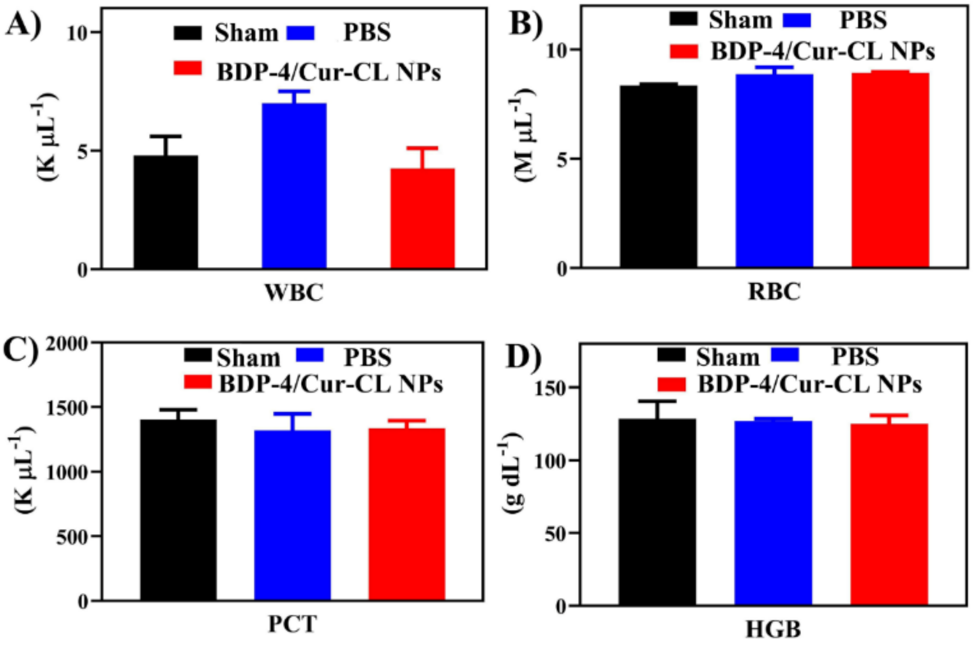

Figure S64. Blood test parameters regarding to A) WBC, B) RBC, C) PCT and D) HGB blood cell count of the mice with different treatment at 28 days. Error bars, mean ± SD (n = 3).

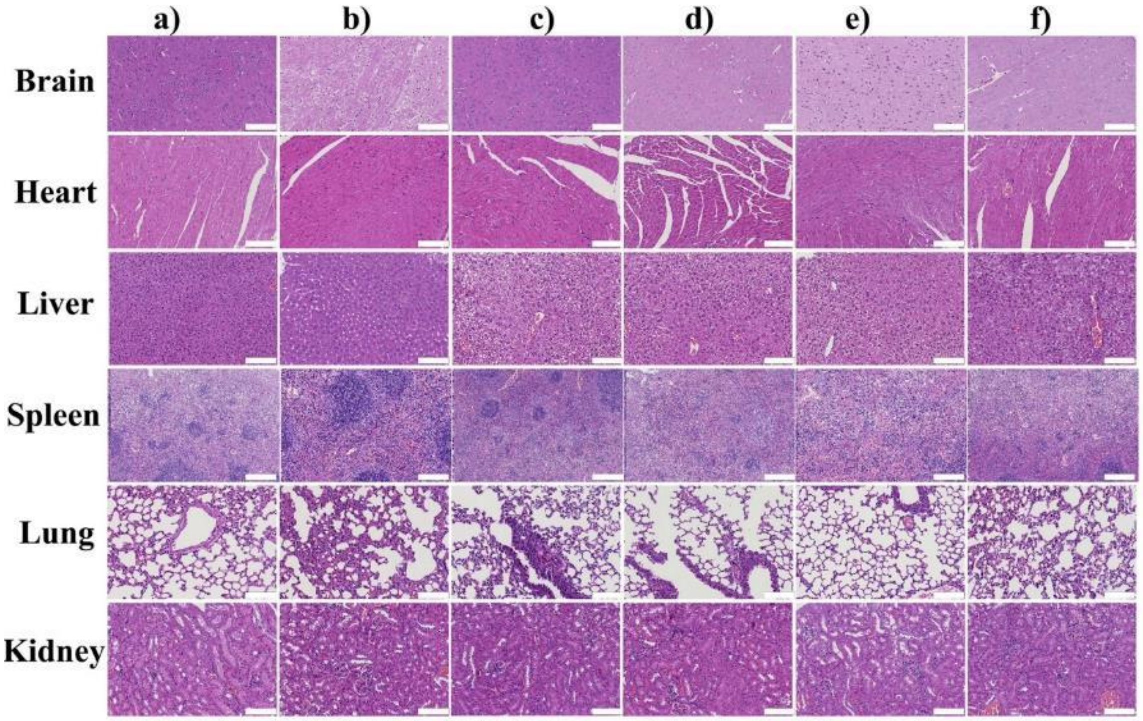

Figure S65. H&E staining of various organs from mice at the end of experiments after treatment a) with sham for 3 days, b) with MCAO for 3 days, c) with MCAO + **BDP-4/Cur-CL** NPs for 3 days, d) with sham for 28 days, e) with MCAO for 28 days, f) with MCAO + **BDP-4/Cur-CL** NPs for 28 days. Representative H&E-stained images from brain, heart, liver, spleen, kidney and lung slices from untreated and **BDP-4/Cur-CL** NPs-treated mice. Scale bars, 100 μm.

References

1. Frisch, M. J. et al., Gaussian09 (RevisionD.01), Gaussian,Inc., Wallingford, Connecticut, **2009**.

2. Chen, C.; Gao, H.; Ou, H.; Kwok, R. T. K.; Tang, Y.; Zheng, D.; Ding, D., Amplification of Activated near-Infrared Afterglow Luminescence by Introducing Twisted Molecular Geometry for Understanding Neutrophil-Involved Diseases. *J. Am. Chem. Soc.* **2022,** *144*, 3429-3441.

3. Yang, Y.; Wang, S.; Lu, L.; Zhang, Q.; Yu, P.; Fan, Y.; Zhang, F., NIR-II Chemiluminescence Molecular Sensor for in Vivo High-Contrast Inflammation Imaging. *Angew. Chem., Int. Ed.* **2020,** *59*, 18380-18385.
